# H4K79 and H4K91 histone lactylation, newly identified lactylation sites enriched in breast cancer

**DOI:** 10.1186/s13046-025-03512-6

**Published:** 2025-08-23

**Authors:** Jiena Liu, Liuying Zhao, Meisi Yan, Shengye Jin, Lingmin Shang, Jianyu Wang, Qin Wang, Shilu Zhao, Zibo Shen, Tong Liu, Hao Wu, Da Pang

**Affiliations:** 1 Department of Breast Surgery, Harbin Medical University Cancer Hospital, Harbin, Heilongjiang Province 150081 China; 2 Department of Pathology, Harbin Medical University, Harbin, Heilongjiang Province China; 3 Heilongjiang Clinical Research Center for Breast Cancer, Harbin Medical University Cancer Hospital, Harbin, Heilongjiang Province 150081 China; 4 Translational Medicine Research and Cooperation Center of Northern China, Heilongjiang Academy of Medical Sciences, Harbin, Heilongjiang Province China; 5 Department of Biomedical and Life Science Faculty, King’s College London, London, UK; 6 Department of Oncology Surgery, Harbin Medical University Cancer Hospital, Harbin, Heilongjiang Province 150081 China; 7 NHC Key Laboratory of Cell Transplantation, Harbin Medical University, Harbin, Heilongjiang Province China

**Keywords:** Breast cancer, Lactylation, Glycolysis, Post-translational modifications, Epigenetic modification

## Abstract

Metabolic reprogramming and epigenetic modification are two hallmarks of cancer. Protein lysine lactylation (Kla) is a novel type of glycolysis lactate-triggered posttranslational modification. However, the role of Kla in breast cancer (BC) remains largely unknown. Here, western blot, and immunohistochemical (IHC) staining of BC tissues revealed that global Kla levels were upregulated in BC tissues, and high levels of Kla were correlated with poor prognosis of patients with BC. A series of in vitro and in vivo assays demonstrated that interruption of glycolysis by lactate dehydrogenase (LDH) inhibitor or silencing LDHA and LDHB repressed the malignant behaviors of BC cells. Moreover, 4D label-free quantitative lactylproteomics analysis of BC tissues and cells revealed that lactylated proteins widely existed in several subcellular compartments and were closely associated with various cancer-related biological processes. Notably, two previously unresearched sites of histone lactylation, H4K79 lactylation (H4K79la) and H4K91 lactylation (H4K91la), were identified to be hyperlactylated in cancer tissues and cells. Glycolytic genes, such as lactate dehydrogenase A (LDHA), phosphoglycerate kinase 1 (PGK1), and hexokinase 1 (HK1) were identified to be the potential candidate genes epigenetically regulated by H4K79la and H4K91la by intersecting through chromatin immunoprecipitation sequencing (ChIP-seq), RNA sequencing (RNA-seq), and TCGA-BRCA database. Pharmacological inhibition of glycolysis downregulated H4K79 and H4K91 lactylation and suppressed the expression of glycolytic genes, whereas treatment with sodium lactate exhibited the opposite effects. Additionally, E1A-binding protein p300 (P300) acted as lysine lactyltransferase to regulate H4K79la and H4K91la, and control the transcription and expression of downstream glycolytic genes in BC cells. The results revealed an intriguing positive feedback loop formed by glycolysis/H4K79la/H4K91la/glycolytic genes in BC, highlighting the relationship between metabolic reprogramming and epigenetic regulation. These findings provide new therapeutic targets for patients with BC.

**Supplementary Information:**

The online version contains supplementary material available at 10.1186/s13046-025-03512-6.

## Graphical Abstract

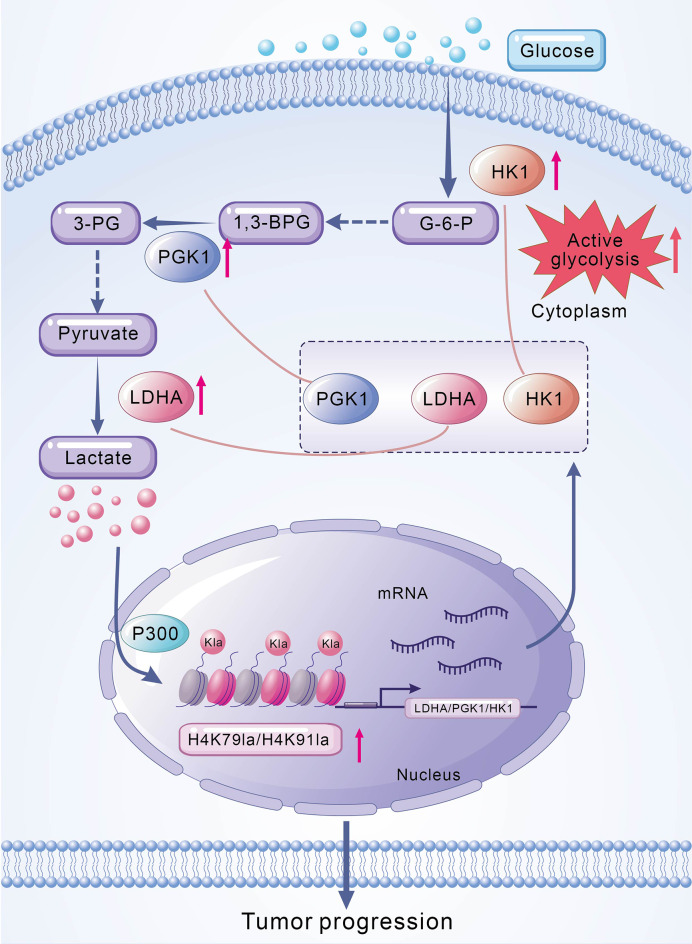

## Introduction

Metabolic reprogramming and epigenetic modification, two hallmarks of cancer, are closely linked and reciprocally regulate each other to support the proliferation and survival of cancer cells [1, 2]. Of note, With the development of high-performance liquid chromatography–tandem mass spectrometry (HPLC-MS), various cellular metabolites, such as acetyl-CoA, α-ketoglutarate (α-KG), nicotinamide adenine dinucleotide (NAD^+^), and S-adenosylmethionine (SAM), have been identified as crucial regulators of epigenetic modifications and gene expression [3–5]. Therefore, a deeper insight and further elucidation of the mechanisms underlying the interactions between metabolic reprogramming and epigenetic modifications in cancer can yield novel insights into tumorigenesis and provide effective treatment options for cancer.

Even in aerobic conditions, cancer cells rely on glycolysis rather than oxidative phosphorylation to provide energy for their high proliferation rate, a phenomenon known as the Warburg effect [2, 6, 7]. Therefore, lactate, the end-product of glycolysis that is produced via the reduction of pyruvate by lactate dehydrogenase (LDH), significantly accumulates in tumor cells compared to normal cells. It is widely known as an energy source and metabolic by-product [8, 9]. Furthermore, subsequent studies have revealed that lactate is a crucial oncometabolite regulating various biological processes, such as tumor proliferation, metastasis, therapeutic resistance, and immune evasion [7, 10–12]. Nevertheless, the non-metabolic functions of lactate in cancer cells remain largely unknown.

Recently, lactate-derived lactylation of protein lysine residue was identified as a novel post-translational modification (PTM), providing a new insight into the non-metabolic functions of lactate and unlocking the mysteries of the Warburg effect [13]. The protein lysine lactylation (Kla) was first reported on histones and functioned as an epigenetic modification that stimulates gene transcription from chromatin [13–17]. As research progressed, Kla was shown to occur ubiquitously in a diverse range of non-histone proteins, including enzymes, signaling proteins, and nucleoproteins [18–20]. Accumulating studies have shown that Kla participates in diverse physiological and pathological processes, encompassing stem cell differentiation [17], neuronal excitation [16, 21], macrophage polarization [22, 23], immune responses [15, 24, 25], signal transduction [26–28], and tumor proliferation [20, 27, 29]. Furthermore, lactylation has been linked to various diseases [30–35], especially, the development and progression of cancers [14, 19, 29, 36–40]. For instance, multi-omics analysis revealed that upregulation of Kla in oral squamous cell carcinoma can promote tumor progression [39]. H3K18 lactylation was found to support the immune escape of non-small cell lung cancer cells by enhancing MYC nuclear transport and the expression of PD-L1 [38]. The widespread distribution of Kla in hepatocellular carcinoma facilitates the proliferation and metastasis of tumor cells [20]. Considering that enhanced glycolysis and extensive lactate accumulation are the common characteristics of cancers, a comprehensive understanding of the mechanisms and roles of Kla can help develop novel therapeutic strategies for treating cancer. However, as a newly discovered PTM, protein Kla is still in its infancy. The lactylome profiles of many tumors still need to be clarified and further in-depth studies on the role of Kla in cancer development and progression are urgently needed.

Based on data from the World Health Organization, breast cancer (BC) is the most common type of cancer and the leading cause of cancer-related death among women worldwide [41, 42]. Although considerable advancements have been achieved in the treatment of BC over the past few decades, the prognosis of patients with BC still remain poor [43–48]. Therefore, unraveling the molecular alterations and the regulatory mechanisms in BC are crucial for devising innovative treatment plans. The lactate level was higher in BC, positively correlated with histological grade, and negatively associated with prognosis [49, 50]. Thus, lactate-derived protein Kla is highly likely to be aberrant in BC, which can serve as a potential target for treatment. However, the regulatory mechanism, downstream protein targets, and the biological roles of Kla remain largely unknown in BC.

In this study, we revealed that the Kla levels were significantly upregulated in BC patients and were correlated with poor prognosis. Furthermore, a comprehensive lactylome profile associated with the development of BC was characterized in this study. For the first time, among the global lactylome profile analysis of BC tissues and cells, we found that histone H4K79 and H4K91 were hyperlactylated. Further mechanistic studies showed that H4K79 lactylation (H4K79la) and H4K91 lactylation (H4K91la) functioned as epigenetic modification that stimulates the transcription of glycolysis-related genes, such as lactate dehydrogenase A (LDHA), phosphoglycerate kinase 1 (PGK1), and hexokinase 1 (HK1), thereby activating glycolysis, promoting the production of lactate, and upregulating lactylation levels. Pharmacological inhibition of glycolysis downregulated H4K79la and H4K91la, and suppressed the proliferation, migration, and invasion of BC cells. Taken together, our data revealed a metabolism-epigenetics-metabolism positive feedback loop in the progression of BC and uncovered the novel roles of protein lactylation, especially H4K79la and H4K91la, in BC. Targeting this glycolysis/H4K79la/H4K91la/glycolytic gene loop may provide a useful therapeutic strategy for patients with BC.

## Materials and methods

### Patients and tissue samples

In total, 234 BC tissues and 28 normal breast tissues were fixed in formalin and embedded in paraffin for immunohistochemistry. The clinical pathological features of these tissue specimens are shown in Table S1. Furthermore, 10 pairs of BC tissues and adjacent normal tissue were immediately frozen in liquid nitrogen and stored at -80℃ for protein and RNA extraction. All human tissues used in this study were collected from patients who underwent surgery at the Department of Breast Surgery, Harbin Medical University Cancer Hospital. The use of clinical samples with patient consent was approved by The Medical Ethics Committee of Harbin Medical University Cancer Hospital.

### Cell culture

Human normal mammary epithelial cell line MCF 10 A, mouse BC cell line 4T1, and human BC cell lines Hs578T, MDA-MB-468, BT-549, T-47D, UACC-812, MCF7, MDA-MB-231and MDA-MB-453 were obtained from the National Collection of Authenticated Cell Cultures (Shanghai, China). All cell lines used in this study were authenticated by STR profiling. MCF 10 A cell were cultured in a specific complete culture medium (CM-0525, Procell). 4T1, Hs578T, MDA-MB-468, UACC-812, MDA-MB-453, MCF7 and MDA-MB-231 cells were cultured in Dulbecco’s Modified Eagle Medium (DMEM; Gibco). BT-549, T-47D, and MDA-MB-453 cells were cultured in Roswell Park Medium Institute − 1640 medium (RPMI-1640; Gibco). Except for MCF 10 A cell, BC cells were cultured in a medium supplemented with 10% certified heat-inactivated fetal bovine serum (FBS; Gibco) and 1% penicillin/streptomycin (Gibco) at 37 °C in a humidified incubator with 5% CO_2_. 4T1, MCF7, MDA-MB-468, and T-47D were treated with different concentrations of (R)-GNE-140 (HY-100742 A, MCE, Shanghai, China) and Sodium lactate (L-7022, Sigma, USA).

### Western blot

Cells and tissues were lysed (1% Triton X-100, 1% protease inhibitor cocktail, 3µM trichostatin A and 50mM nicotinamide) at 4℃ for 30 min, followed by ultrasonic cracking on ice, and then centrifuged at 12,000 g and 4℃ for 10 min, the supernatant was collected and the protein concentration was determined with a bicinchoninic acid kit (P0010, Beyotime Biotechnology). Equal amounts of protein were separated by 8%-15% SDS-PAGE according to their molecular weight and transferred to nitrocellulose (NC) membrane (PA66485, Pall Life Sciences). After blocked with 5% non-fat milk in TBST (25 mM Tris, 150 mM NaCl, 0.1% Tween-20, pH 7.4) for 1 h at room temperature and incubated with appropriate specific primary antibody overnight at 4 °C, the membranes were incubated with corresponding secondary antibody coupled with horseradish peroxidase (HRP) or conjugated to a fluorescent tag for 1 h at room temperature. Then, membranes were visualized using the Odyssey Infrared Imaging System (LI-COR, USA) or Tanon 4600 (Tanon, Shanghai, China). The antibodies used in this study are as follows: pan anti-lactyllysine antibody (PTM-1401RM, PTM Bio), anti-H4K79 (PTM Bio), anti-H4K91 (PTM Bio), anti-LDHA (GTX101416, GeneTex), anti-LDHB (GTX101747, GeneTex), anti-HK1 (19662-1-AP, Proteintech), anti-PGK1 (17811-1-AP, Proteintech), anti-EP300 (14984, Abcam), anti-Histone H4 (HY-80178, MCE), anti-α-tubulin (WL02296, Wanleibio), Goat anti-Rabbit IgG (H + L) IRDye 800CW (926-32211, LI-COR), Goat Anti-Rabbit IgG(H + L) HRP (SA00001-2, Proteintech), Goat Anti-Mouse IgG(H + L) HRP (SA00001-2, Proteintech).

### Immunohistochemistry (IHC)

Paraffin-embedded human and mouse tumor tissues were heated at 65˚C for 3 h, deparaffinized by xylene, and rehydrated by decreased concentrations of ethanol. After antigen retrieval, the samples were treated with 3% H_2_O_2_ and blocked with 5% BSA. The tissue sections were incubated with pan anti-Kla (PTM-1401RM, PTM Bio) primary antibody at 4 °C overnight and then incubated with goat anti-rabbit IgG (PV-6001, ZSGB-BIO) secondary antibody at room temperature for 1 h. The color was developed with DAB (ZLI-9018, ZSGB-BIO) and counterstained with hematoxylin. Final IHC scores were calculated as the product of the extent and intensity of staining. The associations between pan-Kla levels and clinicopathological characteristics are listed in Table S1.

### Antibody production and purification

For the preparation of site-specific antibody used to analyze H4K79la and H4K91la, two modified peptides and one non-modified peptide for each site were designed following the protein sequence, H4K79: modified peptide 1 (CEHAKR-(lactyl)K-TVTAM); modified peptides 2 (CTEHAKR-(lactyl)K-TVTAMD); and non-modified peptide (CTEHAKRKTVTAMD). H4K91: modified peptide 1 (CDVVYAL-(lactyl)K-RQGR); modified peptides 2 (CMDVVYAL-(lactyl)K-RQGRT); and non-modified peptide (CMDVVYALKRQGRT). After validating these peptides by mass spectrometry, antibody production and purification were conducted by Jingjie PTM Bio (Hangzhou, China). Briefly, modified peptides were coupled with keyhole limpet hemocyanin (KLH) to immunize New Zealand rabbits. Anti-serum was collected after four doses of immunization and purified by protein A/G column, modified peptide column, and unmodified peptide column, respectively. The valence and specificity of the site-specific antibody were validated by dot blot and competitive ELISA with the modified peptides and corresponding unmodified peptide.

### RNA interference

The small interfering RNA (siRNA) targeting LDHA and LDHB were designed and synthesized by RiboBio (Guangzhou, China), the siRNA targeting P300 were purchased from GeneChem (Shanghai, China). Transfection of BC cells were conducted through jetPRIME reagent (Polyplus) as recommended. The siRNA sequences used are listed in Table S2.

### RNA sequencing (RNA-seq)

Transcriptome sequencing was conducted on untreated MDA-MB-468 cells as well as those treated with GNE-140 or Sodium lactate. The total RNA was extracted using TRIzol Reagent (15596-026, Invitrogen), after assessed the purity and integrity of total RNA by NanoDrop ND-1000 and Agarose gel electrophoresis, 1–2 µg total RNA was used for each sample to construct the RNA sequencing library at Aksomics Inc (Shanghai, China). Briefly, mRNA was purified using NEBNext Poly(A) mRNA Magnetic Isolation Module (New England Biolabs), the KAPA Stranded RNA-Seq Library Prep Kit (Roche) was used to prepare sequencing libraries and an Illumina NovaSeq 6000 platform was used for sequencing. The clean reads of sequencing were mapped to the human reference genome (GRCh38) using the Hisat2 tool and the mRNA levels of protein-coding genes were normalized and converted into fragments per kilobase million (FPKM). The screening criteria for identifying differential expressed genes (DEGs) included |log2Fold Change| >0.58 and *P* value < 0.05. Subsequently, GO and KEGG functional enrichment analyses were carried out using R software. The sequencing data has been deposited in NCBI BioProject PRJNA1215455.

### RNA extraction and real-time quantitative polymerase reaction (RT-qPCR)

The total RNA from BC tissues or cells were extracted using TRIzol Reagent (Invitrogen, 15596-026), which was then transcribed into cDNA using the Prime Script RT Reagent Kit (Vazyme, R333-01). The real-time qPCR reactions were performed using the SYBR Green Master kit (Roche, 04913914001) and an ABI Prism 7500 system (Applied Biosystems, CA, USA). The cycle threshold (CT) data were analyzed using the 2^−ΔΔct^ method and normalized to beta-actin. The primer sequences for PCR are listed in Table S2.

### Measurement of lactate levels

Lactate levels were measured by using a Lactate Colorimetric Assay Kit II (K627-100, Biovision) according to the manufacturer’s instructions. The absorbance was measured at 450 nm using a microplate reader (BioTek, USA).

### Cell proliferation and colony formation assays

CCK-8 assays were employed to analysis cell proliferation capability. Briefly, a total of 2000–4000 cells were seeded into 96-well plate and treated with corresponding processes. At the indicated time point (0 h, 24 h, 48 h, 72 h), 10µL of CCK-8 reagent (MA0218, meilunbio) was added to each well and incubated in darkness at 37℃ for 1–2 h. The absorbance was detected at 450 nm, and growth curves were generated to determine the growth rates.

For the colony formation assay, a total of 1000–2000 cells were seeded into 6-well plate and cultured for 10–14 days under various conditions or treatments. Subsequently, the cell colonies were fixed with 4% paraformaldehyde and stained with crystal violet solution (C0121, Beyotime).

### Transwell assay

24-well Transwell chambers (3422, Corning) coated with Matrigel (356234, Corning) were employed to measure cell invasion capability. 4.0–8.0 × 10^4^ cells were placed into the upper chamber with 200µL of FBS-free medium, and the lower chamber was filled with 800µL of medium containing 20% FBS. After culturing for 24–48 h, the cells that invaded the lower surface of the chamber were fixed with 4% paraformaldehyde and stained with crystal violet solution (C0121, Beyotime). The stained cells were photographed microscopically, and the number of invaded cells was quantified using Image J software.

### Wound healing assay

The wound-healing assay was used to measure cell migration ability. Cells were seeded in 6-well plate and cultured until form a confluent monolayer, and then a straight-line scratch generated using a sterile pipette tip. The same wound area was photographed by microscope at 0 h, 24 h and 48 h after scratching, the wound areas were quantified using Image J software.

### Mouse tumor model

Five-week-old female BALB/c mice were purchased from Vital River Laboratory Animal Technology (China) and housed under specific pathogen-free (SPF) conditions. All animal experiments were approved by the Ethics Committee and were conducted following Harbin Medical University institutional animal care regulations. Briefly, 5 × 10^4^ 4T1 cells, suspended in sterile PBS and mixed with Matrigel (356234, Corning), were injected subcutaneously into the axilla of mice. When the tumor volume was approximately 100 mm^3^, the mice were randomly assigned to the following groups (*n* = 6 per group): control, GNE140 low-dose group (2.5 mg/kg), and GNE140 high-dose group (5 mg/kg). The experimental mice were treated with the corresponding concentration of GNE140 (dissolved in the cosolvent contained PEG300, Tween-80 and saline) via intragastric administration every two days, while the control group was treated with saline. The tumor volume was measured using a digital caliper and estimated using the following equation: tumor volume = (the longest diameter × shortest diameter^2^) / 2. Finally, all the mice were euthanized after seven doses of GNE140, and the tumors were isolated, photographed, weighted, and processed for paraffin-embedded.

### Chromatin Immunoprecipitation sequencing (ChIP-seq) and ChIP-qPCR

ChIP-seq was conducted on MDA-MB-468 using Pierce Magnetic ChIP Kit (26157, Thermo Fisher Scientific) according to the manufacturer’s instructions. Briefly, cross-linked with formaldehyde (28906, Thermo Fisher Scientific), digested with Micrococcal Nuclease, sonicated to obtain 150–900 bp DNA/protein fragments, and immunoprecipitated with specific antibodies (anti-H4K79la, anti-H4K91la) and Protein A/G magnetic beads. Finally, after reversed the cross-links and treated with Proteinase K, purified DNA fragment were then sended to BGI (Shenzhen, China) for the construction of ChIP-seq library, amplification, and high-throughput DNA sequencing on an BGISEQ-500 platform, The sequencing data has been deposited in NCBI BioProject PRJNA1215530.

ChIP-qPCR was performed on MDA-MB-468 and T-47D cells under various conditions or treatments as indicated to further verify the results of ChIP-seq. In brief, cells were cross-linked, lysed and immunoprecipitated with anti-H4K79la (PTM Bio), anti-H4K91la (PTM Bio), or anti-IgG (2729 S, CST) antibodies. The real-time qPCR reactions of the purified DNA were performed using the SYBR Green Master kit (Roche, 04913914001) and an ABI Prism 7500 system (Applied Biosystems, CA, USA). Fold enrichment was quantified through RT-qPCR analysis and expressed as a percentage of the input chromatin (Input %). The primer sequences for ChIP-qPCR are listed in Table S2.

### 4D label-free quantitative lactylproteomics analysis

4D label-free quantitative lactylproteomics analysis was conducted by Jingjie PTM BioLabs (Hangzhou, China). Briefly, following protein extraction, trypsin digestion, lactyl-modified peptide segment enrichment by Anti-L-Lactyl Lysine Antibody Conjugated Agarose Beads (PTM1404, PTM-Bio), the peptide segments were separated using the ultra-high-performance liquid chromatography system and ionized through the Capillary ion source for ionization. Subsequently, the segments were analyzed with the TimsTOF Pro mass spectrometer. Secondary mass spectrometry data were analyzed using MaxQuant (v1.6.15.0). The Homo_sapiens_9606_SP_20201214.fasta database was used, containing 20,395 sequences. The false discovery rate was set to 1%. The mass spectrometry proteomics data have been deposited to the ProteomeXchange Consortium (https://proteomecentral.proteomexchange.org) via the iProX partner repository [51, 52] with the dataset identifier PXD060185.

### The cancer genome atlas (TCGA) dataset

To evaluate the gene expression profiles of LDHA, PGK1 and HK1 and their correlation with P300, transcriptional landscape of breast invasive carcinoma (BRCA) were downloaded from TCGA (https://portal.gdc.cancer.gov/) database and log2-transformation for normalization. Gene Set Enrichment Analysis (GSEA) evaluating the changes in glycolysis-related signals between BC tissues and normal tissues based on TCGA-BRCA database.

### Statistical analysis

Statistical analysis was performed using GraphPad Prism software (version 8.0.2) and R software (version 4.2.2, https://www.r-project.org). All data were presented as mean ± standard deviation (SD). Student’s t-test or ANOVA was used to assess the statistical significance between two groups or multiple groups. Kaplan–Meier curves and log-rank tests were performed for survival analysis. The relationship between two quantitative variables was estimated by Pearson correlation analysis. Statistical significance was set at *P* < 0.05 (“*” *P* value < 0.05; “**” *P* value < 0.01; “***” *P* value < 0.001; “****” *P* value < 0.0001; ns: not significant).

## Results

### Elevated protein lactylation levels are associated with an unfavorable prognosis in patients with BC

**Fig. 1:**
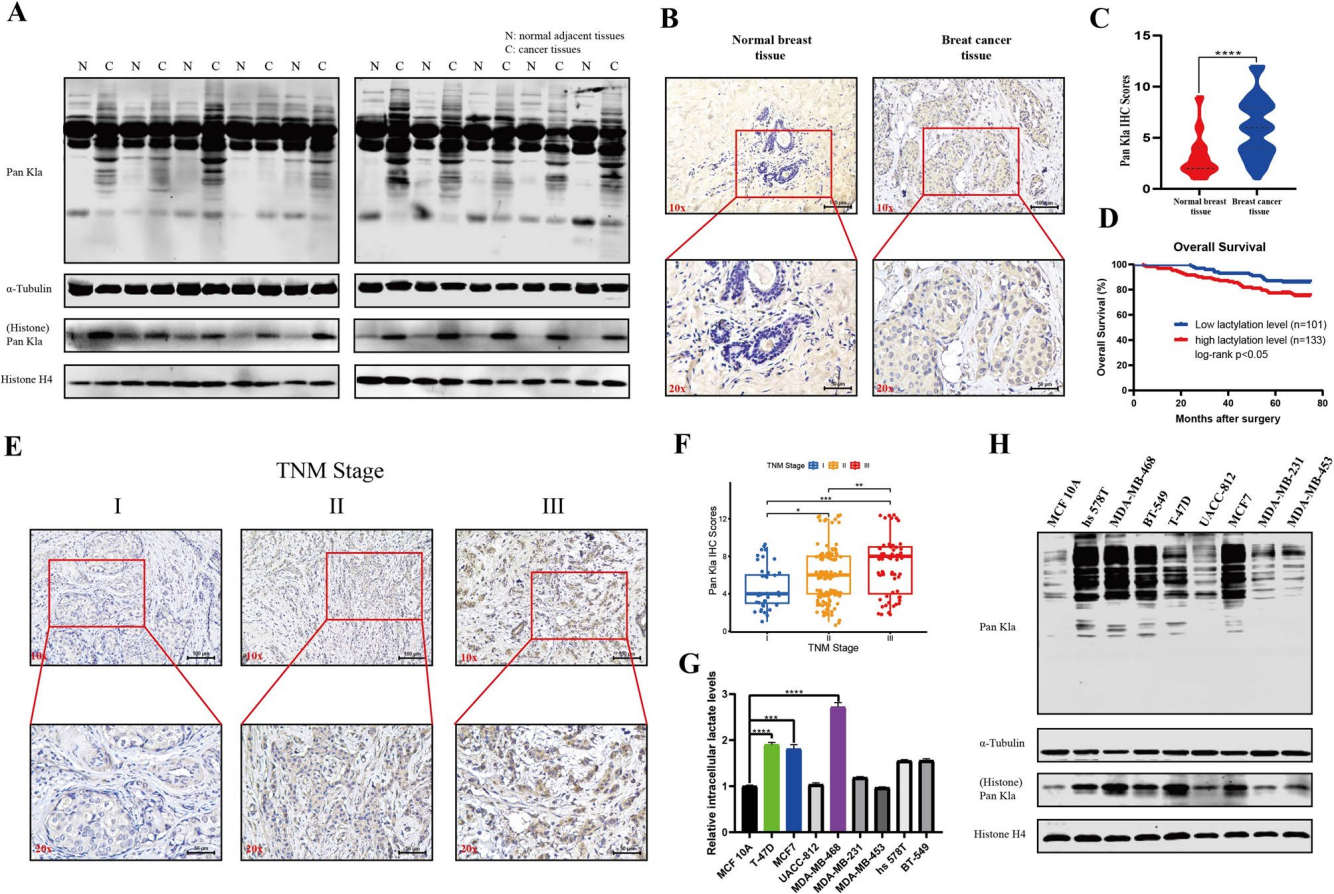
Kla levels were significantly upregulated in BC patients and were correlated with poor prognosis. (**A**). Pan-Kla levels in BC tissues and paired normal adjacent tissues were measured by western blot. (**B**). Representative IHC images of BC tissues and normal tissues. (**C**). Statistical results of lactyation levels in normal and BC tissues. Scale bars: 100 μm (10x); 50 μm (20x). (**D**). Kaplan-Meier analysis and log-rank tests of overall survival in BC patients with low (*n* = 101) and high pan-Kla (*n* = 133) levels. (**E**). Representative pictures of IHC staining of lactylation levels in different TNM stage of BC tissues. Scale bars: 100 μm (10x); 50 μm (20x). (**F**). Statistical results of lactyation levels in different TNM stage of BC tissues. (**G**). Measurement of intracellular lactate levels in BC cells (T-47D, MCF7, UACC-812, MDA-MB-468, MDA-MB-231, MDA-MB-453, hs578T, and BT-549) and human normal mammary epithelial cell line MCF 10 A. (**H**) The lactylation levels in BC cells (T-47D, MCF7, UACC-812, MDA-MB-468, MDA-MB-231, MDA-MB-453, Hs578T, and BT-549) and human normal mammary epithelial cell line MCF 10 A were detected by western blot. Error bars represent the mean ± SD. *P < 0.05, ** *P* < 0.01, *** *P* < 0.001, **** *P* < 0.0001

The Warburg effect (aerobic glycolysis) is a hallmark of cancer [6]. GSEA plots revealed a marked enrichment of the glycolysis/serum lactate-related pathways in patients with BC based on the TCGA-BRCA database (Figure S1a-e). Since lactate acts as a by-product of glycolysis and is regarded as a substrate for protein lysine lactylation (Kla) [13], we measured the global lactylation levels in BC tissues and paired normal adjacent tissues (NAT) through using pan-Kla antibody, western blot analysis revealed the widespread distribution of lactylation throughout the histone and non-histone regions. Cancer tissues exhibited significantly higher levels of global lactylation compared to NATs (Fig. 1A). Subsequently, we measured the Kla level and their clinical correlation by IHC staining of BC tissues and normal breast tissues to assess the significance of protein lactylation in patients with BC. Consistently, IHC staining also indicated a widespread increase in Kla-modified proteins in the nucleus and cytoplasm of BC tissues compared to normal tissues (Fig. 1B, C). Kaplan-Meier analysis revealed that patients with high levels of global Kla had a poorer overall survival and shorter disease-free survival compared to those with low levels of global Kla (Fig. 1D, Figure S1f). Moreover, the correlations between Kla levels and clinicopathological characteristics are summarized in Table S3. Kla levels were positively correlated with higher clinical stage, histological grade, and lymph node metastasis (Fig. 1E, F, Figure S1g-i). However, no significant correlations were found between Kla levels and patients’ age or the molecular subtype of cancer (Table S3). Additionally, consistent with the finding in BC tissues, higher levels of intracellular lactate concentration and global Kla were observed in most BC cells than in human normal mammary epithelial cell line MCF 10 A, especially in T-47D, MCF7, and MDA-MB-468 (Fig. 1G, H). Together, these results revealed that protein lactylation is upregulated in BC tissues and cells, and increased protein lactylation predicts unfavorable prognosis in patients with BC.

### Inhibition of protein lactylation suppressed the development and progression of BC

**Fig. 2:**
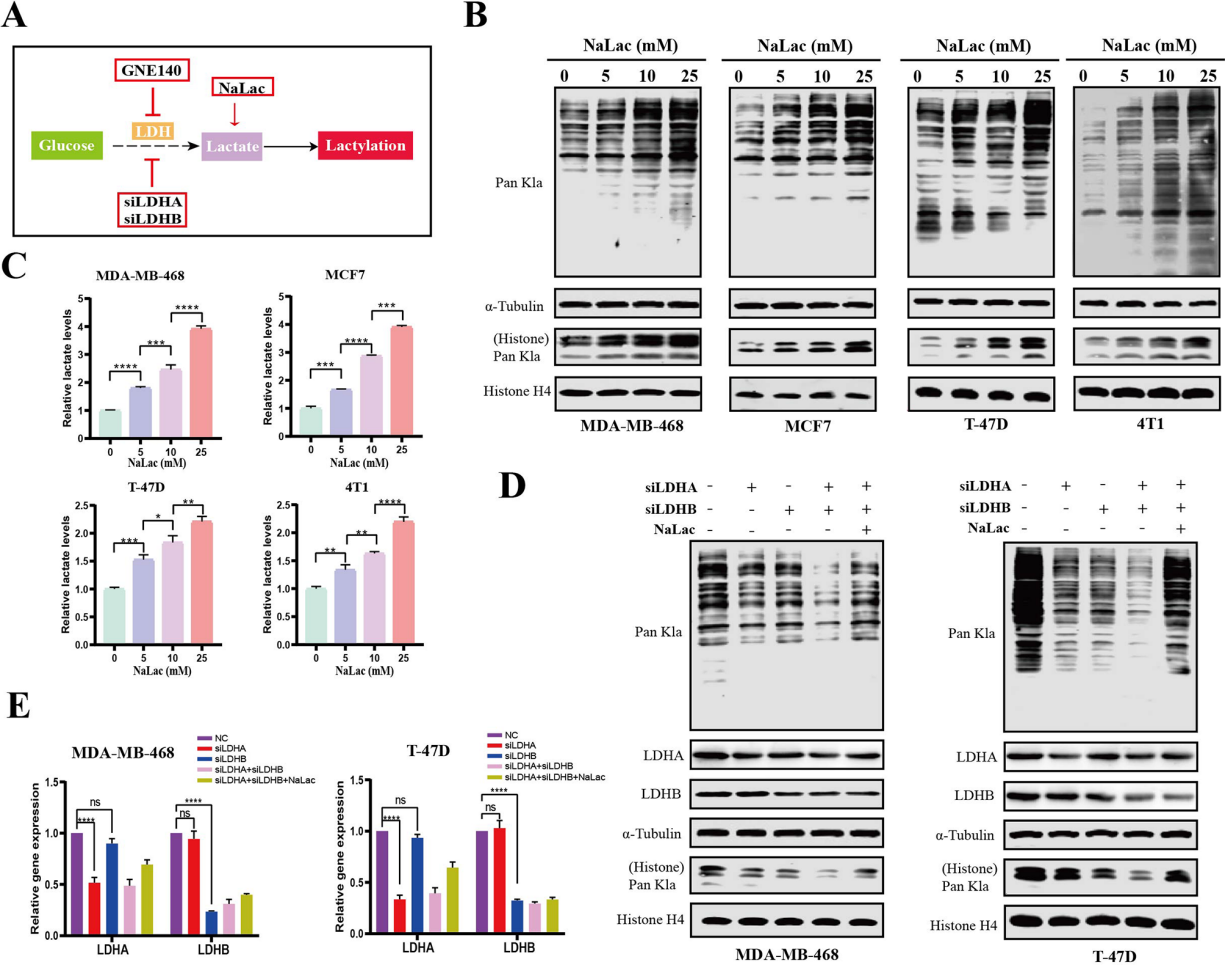
Global lactylation levels in BC cells were regulated by lactate content. (**A**). Schematic representation of experimental design to regulate glycolysis and Kla level. (**B**). Western blot analysis of Pan Kla level in BC cells (MDA-MB-468, MCF7, T-47D, and 4T1) cultured in different concentrations of NaLac for 24 h. (**C**). Measurement of lactate levels in BC cells (MDA-MB-468, MCF7, T-47D, and 4T1) cultured in different concentrations of NaLac for 24 h. (D-E). BC cells were transfected with siLDHA, siLDHB or both siLDHA and siLDHB, then incubated with or without NaLac for 24 h. (**D**). LDHA, LDHB, and Pan Kla levels in MDA-MB-468 and T-47D cells subjected to various treatment groups were analyzed by Western blot. (**E**). Relative mRNA levels of LDHA and LDHB in MDA-MB-468 and T-47D cells subjected to various treatment groups were analyzed by qRT-PCR analysis. * *P* < 0.05, ** *P* < 0.01, *** *P* < 0.001, **** *P* < 0.0001

**Fig. 3:**
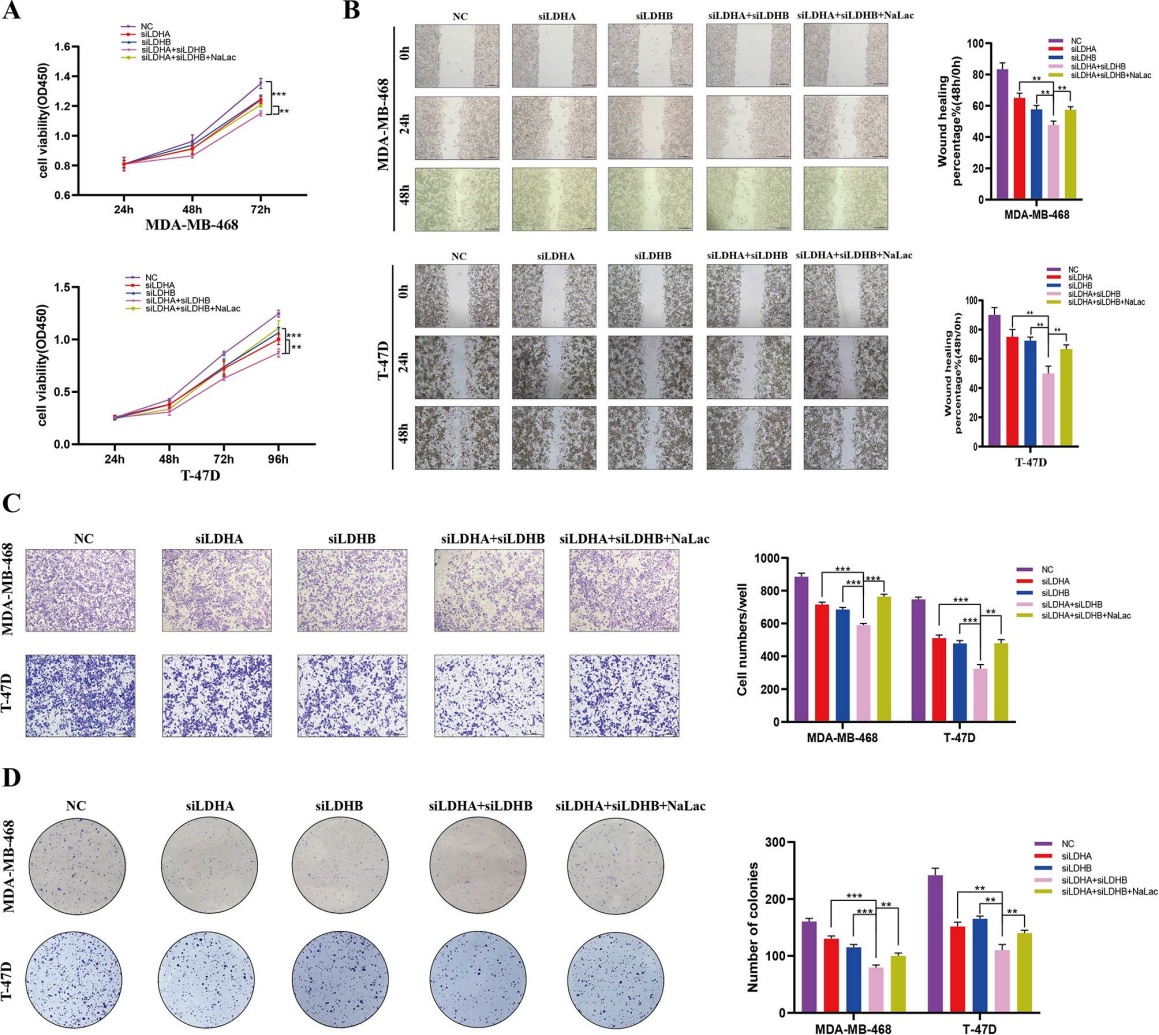
The inhibitory effect on the malignant behavior of BC cells after Simultaneous silencing LDHA and LDHB could be partially restored by adding exogenous NaLac. (**A**-**D**). BC cells were transfected with siLDHA, siLDHB or both siLDHA and siLDHB, then incubated with or without NaLac for 24 h. (**A**). Proliferation ability of MDA-MB-468 and T-47D cells subjected to various treatment groups were analyzed by CCK8 assay. (**B**). Migration ability of MDA-MB-468 and T-47D cells subjected to various treatment groups were analyzed by wounding healing assay. The wound space was photographed at 0, 24 and 48 h. Scale bars: 200 μm. (**C**). Invasion ability of MDA-MB-468 and T-47D cells subjected to various treatment groups were analyzed by transwell assay. Scale bars: 200 μm. (**D**). Proliferation capacity of MDA-MB-468 and T-47D cells subjected to various treatment groups were analyzed by colony formation assays. Relative cell numbers are shown as means ± SD.* *P* < 0.05, ** *P* < 0.01, *** *P* < 0.001, **** *P* < 0.0001

In light of the above findings, we hypothesized that protein lactylation may be associated with the development and progression of BC. To explore the biological functions of protein lactylation in BC, we altered the global lactylation level in BC cells by adding exogenous lactate or attenuating endogenous lactate production (Fig. 2A). As anticipated, both lactate content and protein Kla level were elevated in MDA-MB-468, MCF7, T-47D, and 4T1 BC cells after adding sodium lactate (NaLac) in a concentration-dependent manner (Fig. 2B, C). However, CCK-8 and colony formation assays revealed that NaLac did not significantly affect the proliferative capacity of BC cells (Figure S2a, b). Similarly, wounding healing assay showed the migration ability of BC cells almost did not change after exposure to different concentrations of lactate (Figure S3a, b). Transwell assay showed the invasion ability of cancer cells not obvious influenced even at 25mM of NaLac (Figure S3c, d). In contrast, we found that deletion of either LDHA or LDHB (Fig. 2D, E; Figure S4a-c), two major subunits of LDH, reduced global Kla levels in BC cells (Fig. 2D; Figure S4b, c). Correspondingly, CCK-8 and colony formation assays revealed that siLDHA or siLDHB reduced cell proliferation for both MDA-MB-468 and T-47D cells compared to the control group (Figure S4d-g). Wounding healing and transwell assays indicated that the migration and invasion ability of BC cells were suppressed after treatment with siLDHA (Figure S5a-c). Similarly inhibitory trend was also observed after siLDHB (Figure S5d-f). Notably, simultaneous silencing of LDHA and LDHB strongly enhanced the suppressed effect of protein Kla (Fig. 2D) and significantly inhibited cell proliferation (Fig. 3A), migration (Fig. 3B), invasion (Fig. 3C), and colony formation (Fig. 3D), while NaLac supplementation to LDHA and LDHB-deficient cancer cells restored global protein Kla level (Fig. 2D), thereby attenuating the suppressive effect of simultaneous silencing of LDHA and LDHB (Fig. 3), indicating that decreased protein Kla suppressed the malignant behavior of BC cells and this inhibitory effect could be partially restored by adding exogenous NaLac. A plausible explanation of this phenomenon could be that the levels of Kla had reached saturation in the high-lactate environment of BC cells and were sufficient for tumorigenesis. Further increase in Kla levels did not significantly affect the malignant behavior of BC cells. However, decreased lactate and Kla levels exhibited a more pronounced impact on BC cells.

**Fig. 4:**
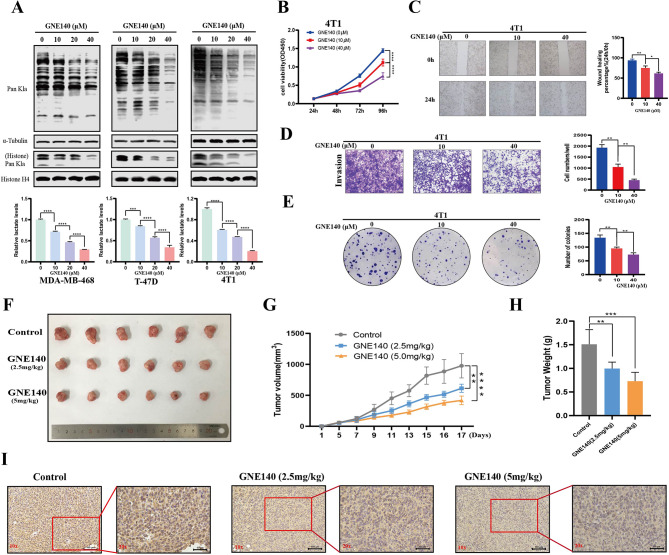
Glycolysis inhibitor reduced global Kla levels and inhibited the malignant behavior of BC cells both in vitro and in vivo. (**A**). Measurement of the lactate content and Pan Kla level in MDA-MB-468, T-47D, and 4T1 cells cultured in different concentrations of GNE140 for 24 h by Western blot and Lactate Colorimetric Assay Kit. (**B**). Cell proliferation ability was evaluated by CCK8 assays in 4T1 cell after treatment with different concentrations of GNE140. (**C**). Cell migration ability was evaluated by wounding healing assay in 4T1 cell after treatment with different concentrations of GNE140. The wound space was photographed at 0 and 24 h. Scale bars: 200 μm. (**D**). Cell invasion ability was evaluated by transwell assays in 4T1 cell after treatment with different concentrations of GNE140. Scale bars: 200 μm. (**E**). Cell proliferation ability was evaluated by colony formation assays in 4T1 cell after treatment with different concentrations of GNE140. (**F**). General pictures of tumor tissues demonstrated the suppressive effect of GNE140 in 4T1-bearing mice model (*n* = 6). Tumor growth curves (**G**) and tumor weight (**H**) were shown in control and different concentrations of GNE40 treatment groups (2.5 mg/kg, 5.0 g/kg). (**I**). Representative IHC images with Pan-Kla of mouse tissues in each treatment groups. Scale bars: 100 μm (10x); 50 μm (20x). Error bars represent the mean ± SD. * *P* < 0.05, ** *P* < 0.01, *** *P* < 0.001, **** *P* < 0.0001

**Fig. 5:**
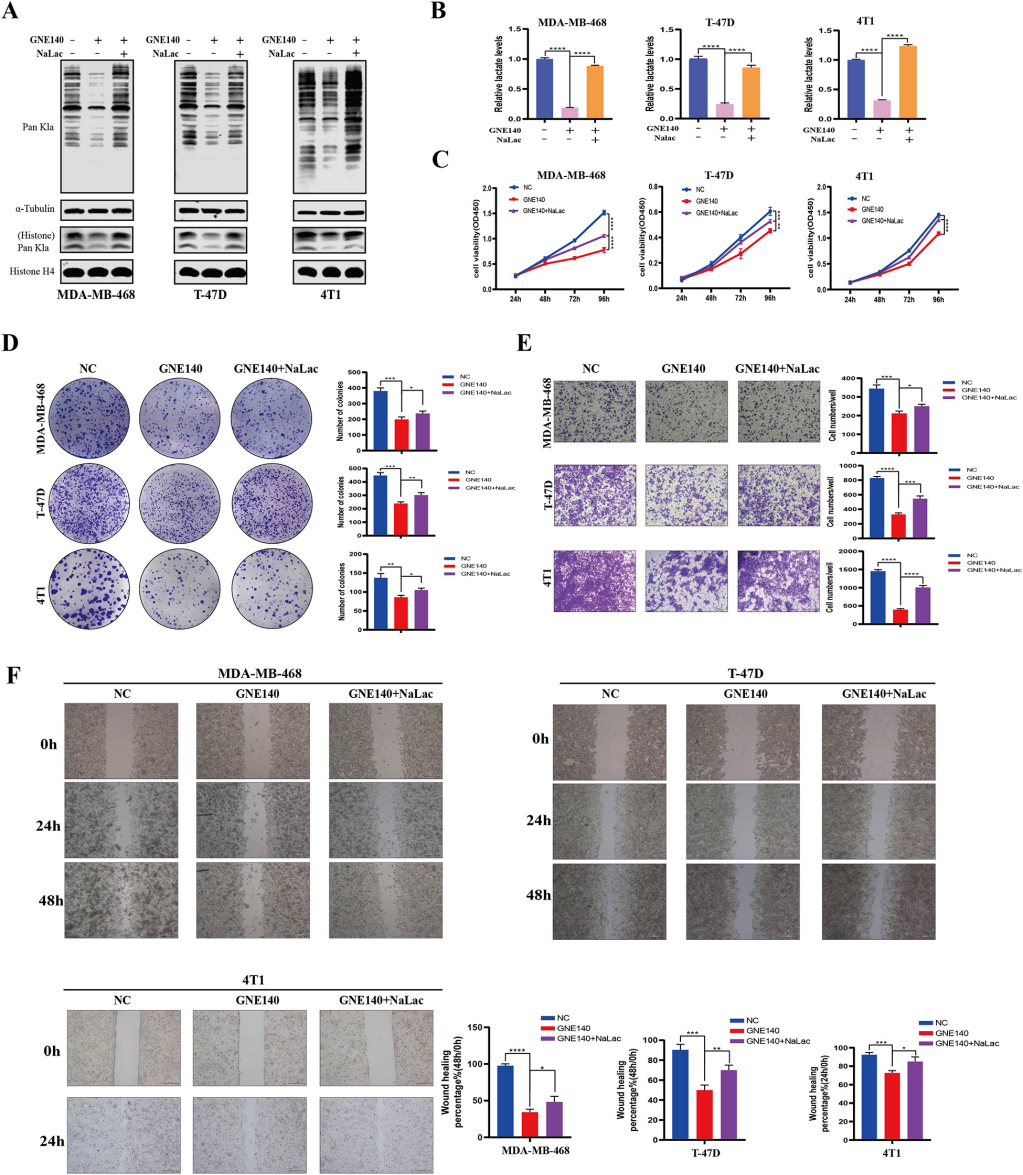
The inhibitory effect on global Kla levels and the malignant behavior of BC cells after GNE140 treatment could be partially restored by adding exogenous NaLac. (**A**-**F**) BC cells were treated with GNE140 then incubated with or without NaLac for 24 h. (**A**, **B**) Pan Kla and Lactate levels in different group of MDA-MB-468, T-47D and 4T1 cells were measured by Western blot and Lactate Colorimetric Assay Kit. (**C**-**F**). Proliferation, invasion and migration capabilities of BC cells in different treatment groups were analyzed by CCK8 assay (**C**), colony formation assay (**D**), transwell assay (**E**), and wounding healing assays (**F**), respectively. Error bars represent the mean ± SD. * *P* < 0.05, ** *P* < 0.01, *** *P* < 0.001, **** *P* < 0.0001. Scale bars: 200 μm

Moreover, the LDH inhibitor (GNE140) reduced lactate level in a dose-dependent manner and the level of global protein Kla was markedly downregulated in line with the decreased lactate level (Fig. 4A). In parallel, the inhibitory effects of GNE140 on the proliferation, migration, and invasion ability of 4T1 BC cells occurred in a concentration-dependent manner (Fig. 4B-E). Similarly, GNE140 inhibited the malignant biological behaviors of MDA-MB-468 and T-47D cells, which was more pronounced at higher concentration than lower concentration (Figure S6). Next, we evaluated the biological significance of protein Kla in vivo. The subcutaneous mouse tumor model was constructed and treated with different doses of GNE140 or vehicle. The GNE140-treated groups exhibited a significant reduction in tumor volume and weight compared to the control group, and the group receiving higher doses showed a more pronounced inhibition of tumor progression compared to lower dose group (Fig. 4F-H). Consistently, similar to in vitro findings, IHC staining of mouse tumor tissues revealed a gradient downregulation in Kla levels compared to the control group (Fig. 4I). In addition, in vitro experiments further demonstrated that adding back NaLac into GNE140-treated BC cells recovered lactate and Kla levels in cells (Fig. 5A, B), and partly restored cell proliferation (Fig. 5C, D), invasion (Fig. 5E), and migration (Fig. 5F) abilities consequently.

Collectively, these in vitro and in vivo findings suggest that lactate level is a key determinant of Kla, and inhibition of protein Kla may exhibit potential antitumor activity against BC.

### Characterization of the lactylome in BC tissues and cells

**Fig. 6:**
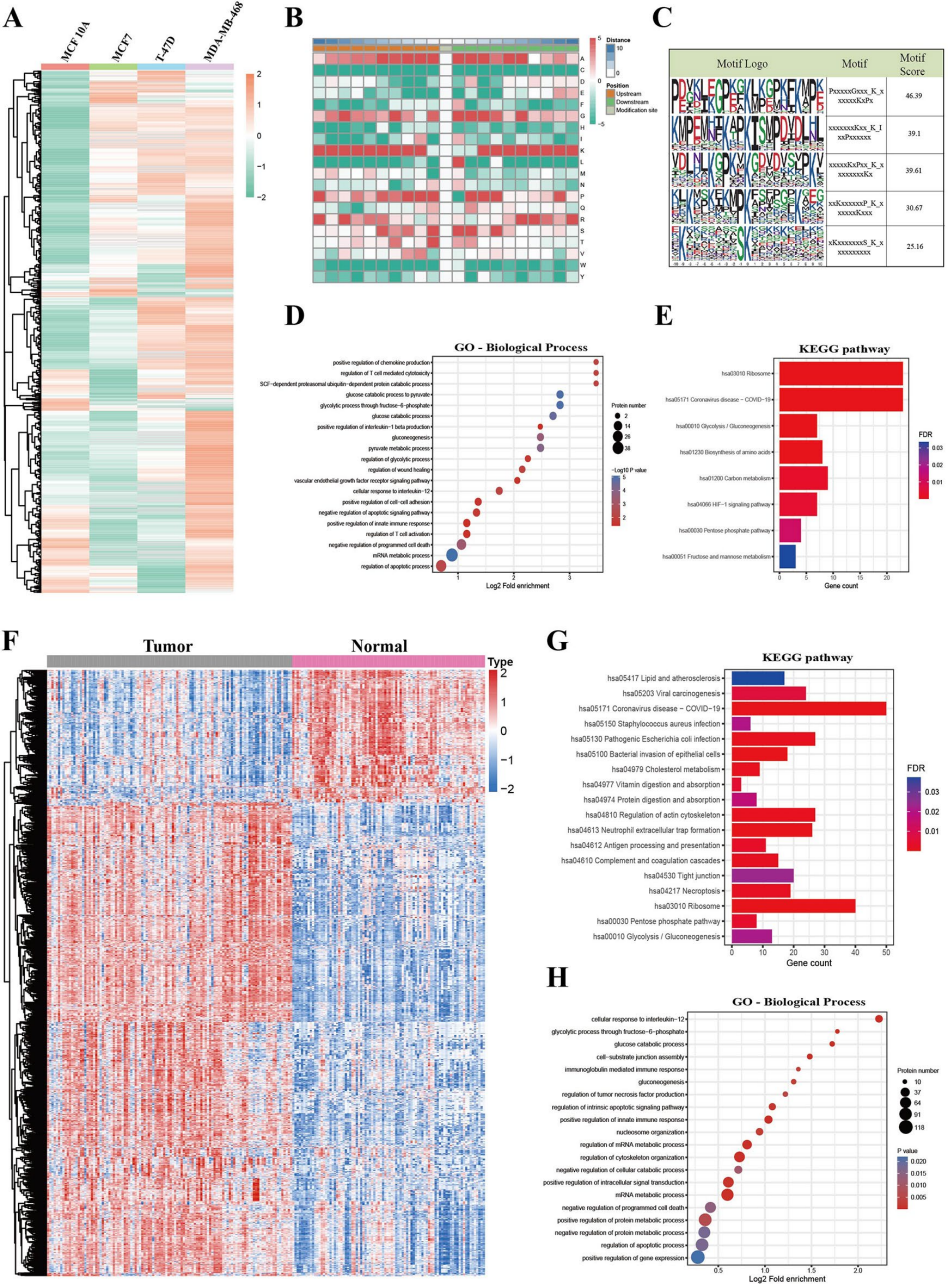
Characterization of the lactylome in BC tissues and cells. (**A**). A heatmap displaying various up-regulated and down-regulated protein lactylation sites in BC cells (MCF7, T-47D, and MDA-MB-468) as compared to normal epithelial cell (MCF10A). Each row represents one lactylation modification site, and each column represents a different type of breast cell lines (MCF 10 A, MCF7, T-47D, and MDA-MB-468). Red represents high expression, green indicates low expression, and gray denotes non-quantifiable values in the corresponding sample. (**B**). Heatmap of the 21 amino-acid compositions of the Kla site showing the frequency of the different amino acids in the specific upstream (orange) 10 amino acids or downstream (green) 10 amino acids positions of the lactylated lysine. Different colors represent the preference of each residue in the position of a 21 amino-acid-long sequence context (red indicates greater possibility, whereas green refers to less possibility). (**C**) The ten amino acids up- and downstream of the Kla using Motif-X are analyzed and the top five motifs with the highest scores are shown. The height of each letter corresponds to the frequency of that amino-acid residue in that position. The central K refers to the lactylated lysine. (**D**). GO-BP enrichment analysis of common DELPs identified in BC cells (MCF7, T-47D, and MDA-MB-468). (**E**). KEGG pathway enrichment analysis of common DELPs identified in BC cells. (**F**-**H**). lactylproteomics analysis of 113 tumor tissues and 88 NAT samples. (**F**). Heatmap of differentially expressed lactylation sites between BC tissues (grey) and normal tissues (pink bar). Each row represents one lactylation modification site, and each column represents a different tissue sample. Red represents high expression, blue indicates low expression, and gray denotes non-quantifiable values in the corresponding sample. (**G**). KEGG pathway enrichment analysis of DELPs identified in BC tissues. (**H**). GO-BP enrichment analysis of DELPs identified in BC tissues

Considering the role of protein Kla in the development and progression of BC, we next attempted to gain a global overview of lactylome in BC cells and tissues. 4D label-free quantitative lactylproteomics analysis was performed to identify the differentially lactylated proteins between human normal mammary epithelial cell line (MCF 10 A) and BC cells (MCF7, T-47D, and MDA-MB-468). In total, 5495 lactylation sites on 1987 proteins were identified (Figure S7a; Table-S4). After normalizing the abundance of Kla to the expression of their corresponding proteins, the heatmap revealed that various upregulated and downregulated protein lactylation sites were detected in BC cells compared to normal epithelial cell, notably, a widespread upregulation rather than downregulation of Kla levels was observed in BC cells (Fig. 6A). Furthermore, comprehensive analysis with the upregulation defined by a fold change exceeding 1.5 and downregulation by a fold change less than 1/1.5 revealed a widespread upregulation of lactylation levels in each BC cells, among them, 829 sites of 488 proteins were upregulated and 28 sites of 26 proteins were downregulated in MDA-MB-468 BC cells compared to normal cells, 393 sites of 257 proteins were upregulated and 220 sites of 160 proteins were downregulated in MCF-7 BC cells compared to normal cells, 434 sites of 302 proteins were upregulated and 170 sites of 144 proteins were downregulated in T-47D BC cells compared to normal cells (Figure S7b), which was in agreement with our findings showing that the levels of lactate and global lactylation were higher in BC cells than in normal epithelial cell (Fig. 1G, H). Integrative lactylomic alterations of common differentially expressed lactylated proteins (DELPs) showed that 222 sites of 167 proteins were all upregulated in cancer cells compared to normal cells, whereas only one site of one protein was downregulated in cancer cells (Figure S7c). Moreover, subcellular localization analysis of these common DELPs indicated a wide distribution across various cellular compartments, such as the nucleus (50.6%), cytoplasm (36.9%), mitochondria (4.76%), and extracellular (4.79%) (Figure S7d). To investigate the amino acid sequence preference for Kla in BC cells, Kla Motif analysis of the peptide sequences (10 amino acids) upstream and downstream surrounding the identified Kla sites was performed using Motif-X, and results revealed that the Kla sites preferably located downstream of glycine, lysine, proline, or serine residues and upstream of lysine or isoleucine (Fig. 6B-C). Next, GO and KEGG pathway enrichment analyses were conducted to explore the potential function of these DELPs. The results unveiled a significantly enrichment of cellar metabolism-related pathway, immune-related and cell death-related pathways, such as glycolysis/gluconeogenesis, biosynthesis of amino acids, pentose phosphate pathway, mRNA metabolic process, regulation of T cell-mediated cytotoxicity, and regulation of apoptotic process (Fig. 6D-E, Figure S7e-f).

Based on the inspiring lactylproteomics analysis results in BC cells compared with normal mammary epithelial cell, lactylproteomics analysis of 113 tumor tissues and 88 NAT samples was conducted to further obtain a comprehensive molecular understanding of Kla in the development and progression of BC. As expected, a notable and widespread upregulation of lactylation levels was found in tumor tissues compared to NAT samples (Fig. 6F). A quantitative comparison of the lactylome data using the criterion of *P*-value < 0.05 and fold change > 1.5 identified 1310 sites on 468 proteins with significantly increased lactylation levels in BC tissues, while 368 sites on 86 proteins with significantly decreased lactylation in cancer tissues compared to normal tissues (Figure S7g). Similarly, the majority of DELPs that were identified in tumor tissues were also localized in the nucleus (28.44%) and cytoplasm (39.49%) (Figure S7h), suggesting that Kla may exert profound biological functions by modifying nuclear and cytosolic proteins. GO and KEGG analysis of the DELPs identified in cancer tissues indicated their potential regulatory roles in metabolism, gene expression, signal transduction, and other cancer-related pathways (Fig. 6G-H, Figure S7i-j).

According to the functional enrichment analysis results of DELPs in BC cells and cancer tissues (Table-S5), numerous enzymes that participated in the regulation of cell-metabolism process were identified to be differentially expressed lactylated proteins. For instance, in Glycolysis/ Gluconeogenesis and Biosynthesis of amino acids pathways, lysine 131 (K131) site of PGK1, K322 and K13 sites of ALDOA, K215 and K194 sites of GAPDH, K89 site of ENO1, K678 site of PFKM were hyper-lactylated in cancer cells and tissues. Furthermore, K49 site of ANXA2, the protein that enrichment in the biological process of angiogenesis, was also Kla upregulated. In addition, modified sites identified on DELPs, for example, six differentially expressed lactylated sites (K577, K467, K71, K477, K132, and K223) were identified in NCL, further research is needed to identify and screen the modification sites that exert corresponding functions.

Altogether, we comprehensively characterized the landscape of Kla in BC tissues and cells, suggesting a potential association between increased Kla and the development and progression of BC.

### BC tissues and cells exhibited elevated histone H4K79 and H4K91 lactylation levels

**Fig. 7:**
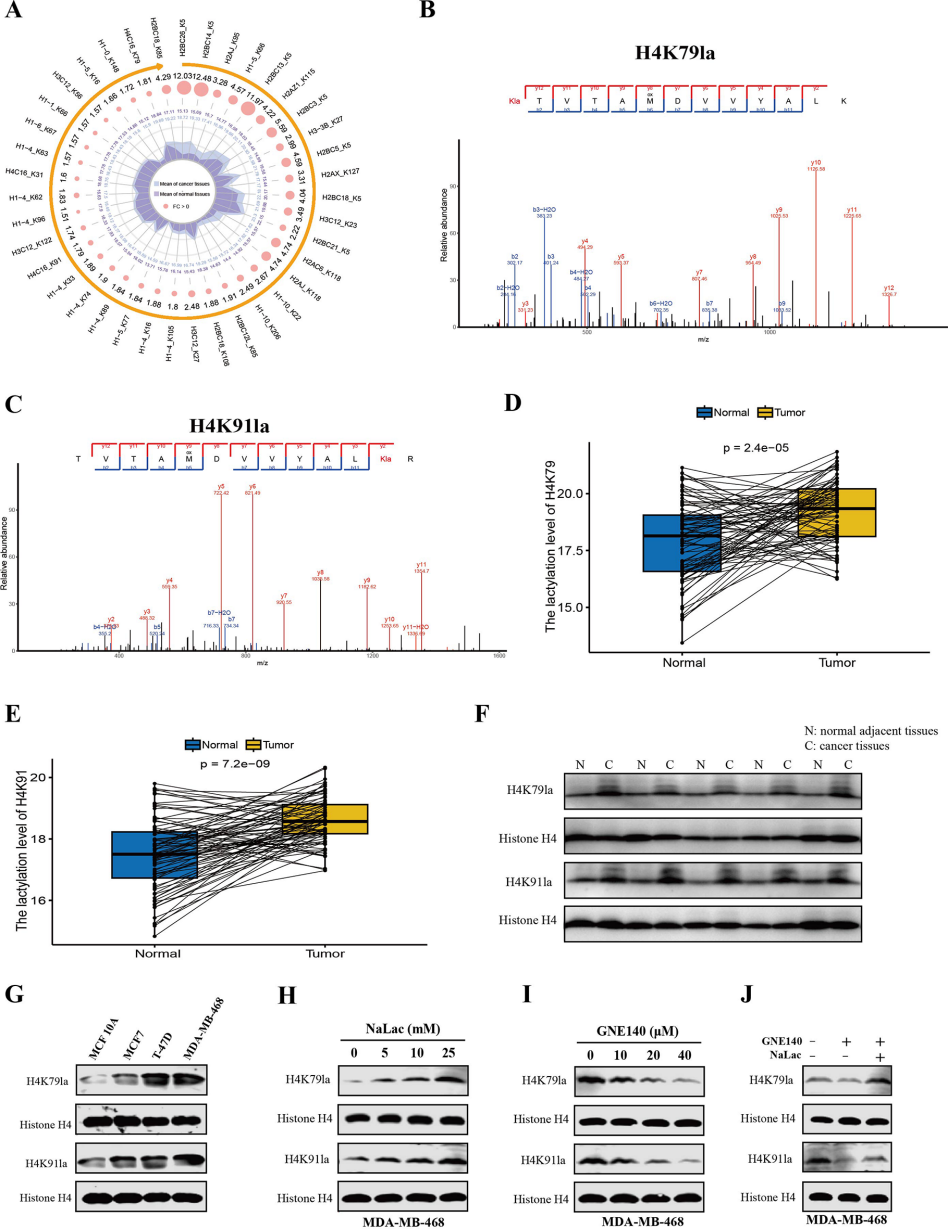
BC exhibited elevated levels of H4K79la and H4K91la and regulated by glycolysis and lactate levels. (**A**). Quantitative comparative analysis of the abundance of Kla sites on histone was shown by radar map, the inner circle represents the mean log2-normalized quantitative values of the Kla levels at specific sites in tumor (gray) and normal (purple) tissues, the numbers represent the FC values of tumor tissue/normal tissue, larger FC values correspond to larger dots diameters, and outside circle represents differentially expressed histone lactylation modification sites identified in BC tissues. (**B**). Mass spectrometry extracted from lactylproteomics data verified the lactylation modification at H4K79. (**C**). Mass spectrometry extracted from lactylproteomics data verified the lactylation modification at H4K91. (**D**, **E**). Paired analysis of the H4K79la (**D**) and H4K91la (**E**) levels in tumor tissues and their paired normal adjacent tissues. (**F**). Western blot analysis of H4K79la and H4K91la levels in BC tissues and paired normal adjacent tissues. (**G**). Western blot analysis of H4K79la and H4K91la levels in BC cells and normal mammary epithelial cell (MCF 10 A). (**H**-**I**). Western blot analysis of H4K79la and H4K91la levels in MDA-MB-468 cell cultured in different concentrations of NaLac (**H**) or treated with different doses of GNE140 (**I**). (**J**) Western blot analysis of H4K79la and H4K91la levels in MDA-MB-468 cell treated with GNE140 then incubated with or without NaLac

Recently, lactate-triggered histone Kla has been identified as a novel type of epigenetic modification that plays a pivotal regulatory role during tumor development and progression [29, 53–55]. However, the mechanism underlying histone Kla in BC remains poorly understood. Through quantitative comparative analysis the abundance of Kla sites on histone, a prevalently elevation of histone Kla levels was found in tumor tissues (Fig. 7A). Notably, among the upregulated lactylation sites, histone H4K79 and H4K91 sites caught our attention since their lactylation levels were higher in both BC tissues and cells (Fig. 7A, Figure S8 a, b). In addition, there is currently a research gap on the mechanism and function of these two histone Kla sites. Mass spectrometry data extracted from the lactylproteomics data validated the existence of Kla at these two histone sites (Fig. 7B, C). Paired analysis of lactylproteomics data revealed that the lactylation levels of H4K79 and H4K91 were higher in tumor tissues compared to their paired normal adjacent tissues (Fig. 7D, E).

Subsequently, we used site-specific H4K79la and H4K91la antibodies to detect the levels of H4K79la and H4K91la in tumor tissues and cancer cells and facilitate further studies on H4K79 and H4K91 lactylation (hereafter referred to as H4K79la and H4K91la, respectively). The verification data of the specialized anti-H4K79la and anti-H4K91la antibodies are provided in Figure S8. Additionally, consistent with the lactylproteomics data, Western blot using these two specialized antibodies showed that H4K79la and H4K91la were upregulated in tumor tissues compared to normal adjacent tissues (Fig. 7F, Figure S9a). Consistently, IHC staining also indicated increased H4K79la and H4K91la levels in BC tissues compared to normal tissues (Figure S9b, c). Similarly, BC cells exhibited obviously increased H4K79la and H4K91la levels than normal epithelial cell (Fig. 7G). Furthermore, a concentration-dependent increase in H4K79la and H4K91la levels was observed after treatment with NaLac in MDA-MB-468 cell (Fig. 7H). In contrast, treatment with GNE140 revealed the opposite effect (Fig. 7I), while adding back NaLac into GNE140-treated MDA-MB-468 cells recovered H4K79la and H4K91la levels (Fig. 7J). Similar alterations in H4K79la and H4K91la levels were observed in T-47D and 4T1 cancer cells under various treatments (Figure S9d-f), confirming the existence and regulation of H4K79la and H4K91la.

Taken together, these findings indicated that H4K79la and H4K91la are upregulated in BC tissues and cells, and regulated by glycolysis and lactate levels.

### Glycolysis/H4K79la/H4K91la/glycolytic genes form a positive feedback loop in BC

**Fig. 8:**
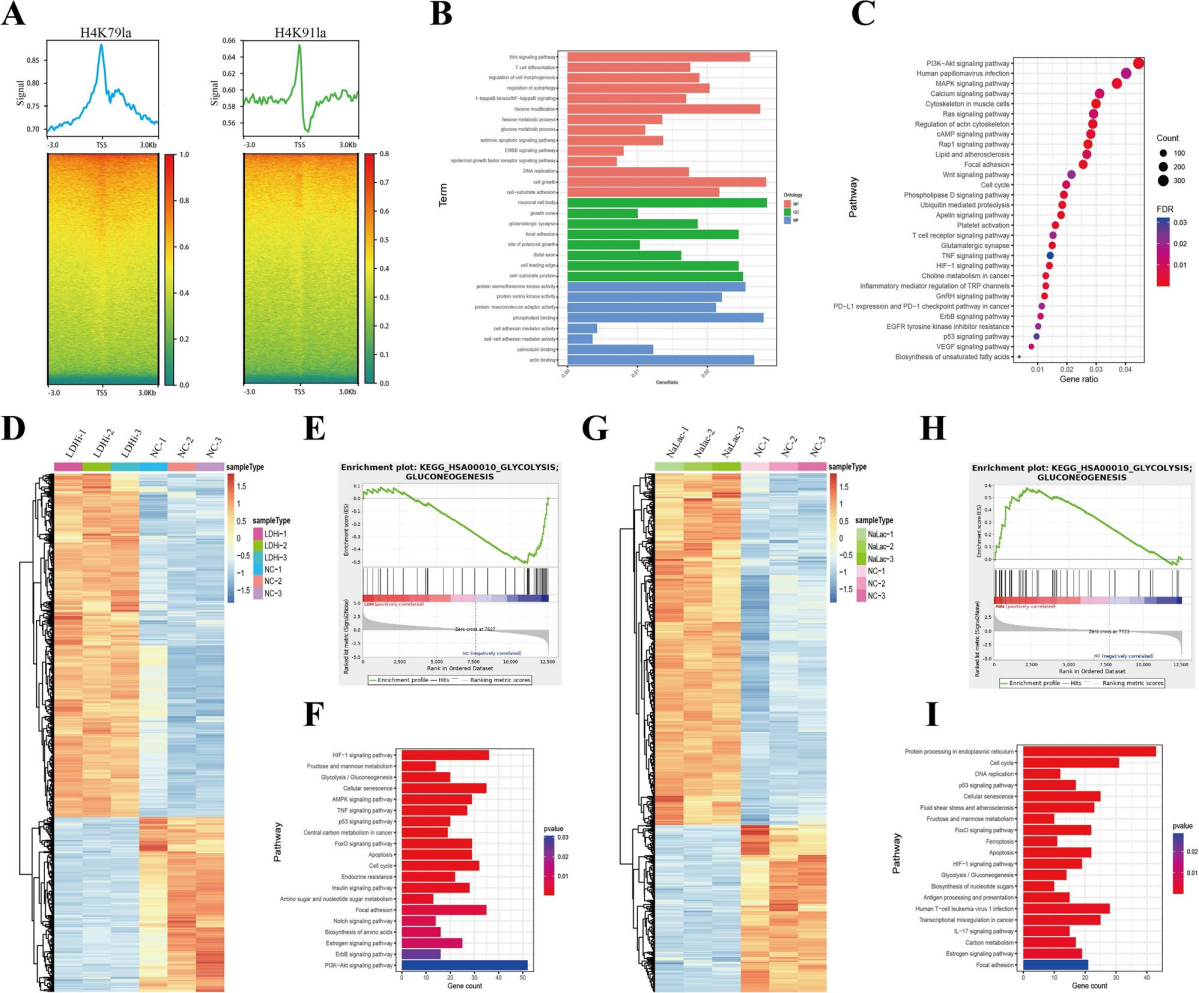
ChIP-seq analysis of H4K79la and H4K91la, and RNA-seq analysis of cells treated with NaLac or LDH inhibitor. (**A**). The heatmaps for H4K79la and H4K91la binding peaks in the transcription start site. Color depth indicates the relative number of reads. (**B**). GO analysis of H4K79la-realted promoter peak genes. (**C**). KEGG analysis of H4K79la-realted promoter peak genes. (**D**). Heatmap of differentially expressed genes in GNE140-treated group based on RNA-seq. (**E**). GSEA analyses of Glycolysis/Gluconeogenesis pathway based on the RNA-seq data from GNE140-treated group versus control group. (**F**). KEGG analysis of differentially expressed genes after GNE140 treatment. (**G**). Heatmap of differentially expressed genes in NaLac-treated group based on RNA-seq. (**H**). GSEA analyses of Glycolysis/Gluconeogenesis pathway based on the RNA-seq data from NaLac-treated group versus control group. (**I**). KEGG analysis of differentially expressed genes after NaLac treatment

Histone modifications play critical role in regulating the transcription of genes from chromatin. Therefore, we conducted ChIP-seq in MDA-MB-468 cell using anti-H4K79la or anti-H4K91la antibodies to identify the potential target genes and comprehensively elucidate the potential biological functions of H4K79la and H4K91la in BC. ChIP-seq data showed H4K79la and H4K91la binding signals in gene promoter regions (± 2 kb around transcriptional start sites) (Fig. 8A), indicating that H4K79la and H4K91la enrich in gene promoter regions. Motif sequence analysis of H4K79la and H4K91la peaks is provided in Figure S10a-b and Table-S8, 9. Notably, GO and KEGG analysis of H4K79la-related promoter peak genes revealed enrichment in glucose metabolic process, cell cycle, focal adhesion, and cancer-related signaling pathways, such as Wnt, PI3K-Akt, MAPK, and TNF pathways (Fig. 8B, C). Concurrently, GO and KEGG analysis of H4K91la-related genes also showed enrichment in metabolism-related and cancer-related pathways (Figure S10c-d), implying the profound biological functions of H4K79la and H4H91la in BC.

**Fig. 9:**
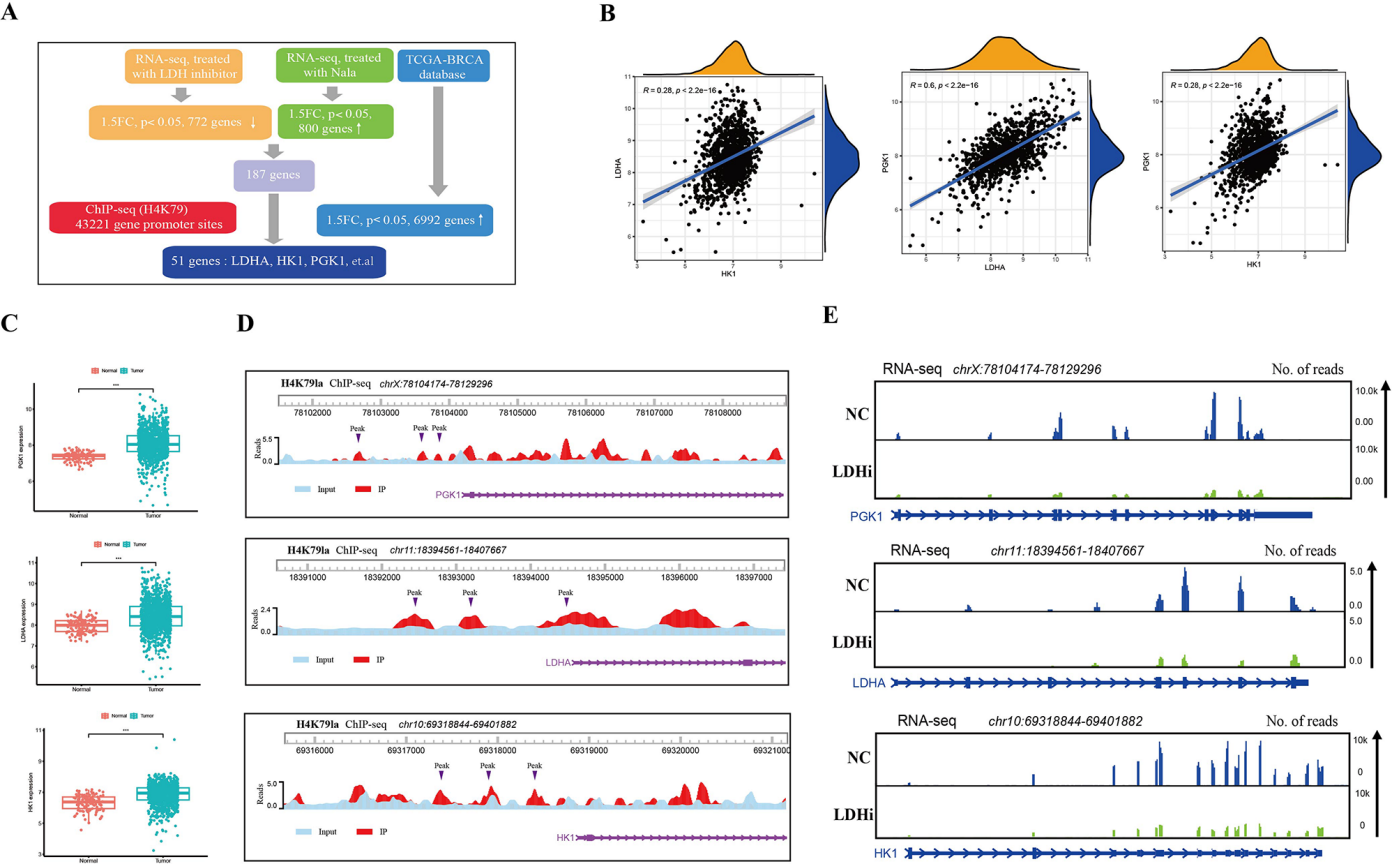
Identification of the potential target genes of H4K79la. (**A**). Combination of ChIP-seq, RNA-seq and TCGA-BRCA database to identify the potential downstream targets of H4K79la. (**B**). A scatterHist showing the correlation among the expression of LDHA, PGK1 and HK1 based on the TCGA-BRCA cohort. Significance was determined by Pearson correlation analysis. (**C**). Differential expression of LDHA, PGK1 and HK1 in TCGA-BRCA cohort. (**D**). Representative IGV tracks showing enriched H4K79la modification in PGK1, LDHA, and HK1 promotor regions by ChIP-seq. Arrows are the H4K79la peaks at the gene promotor. (**E**). Representative IGV tracks showing decreased PGK1, LDHA, and HK1 expression upon LDH inhibitor treatment by RNA-seq

Additionally, we conducted RNA-seq analyses on MDA-MB-468 cells treated with NaLac or LDH inhibitor (GNE140) to identify the potential candidate genes epigenetically regulated by H4K79la and H4K91la. We identified 2287 differentially expressed genes (DEGs) in the LDH inhibitor treatment group compared to the control group, consisting of 1515 upregulated and 772 downregulated genes (Fig. 8D, Figure S10f, Table-S6). As expected, GSEA plots revealed a decreased enrichment of the Glycolysis/Gluconeogenesis pathway in the LDH inhibitor treatment group (Fig. 8E). GO and KEGG pathway analysis revealed that these DEGs were significantly associated with metabolic pathways and various tumor-related pathways (Fig. 8F, Figure S10e). Moreover, we identified 1183 DEGs with fold change > 1.5 in the NaLac treatment group, including 800 upregulated and 383 downregulated genes (Fig. 8G, Figure S10g, Table-S7). In contrast, GSEA plots revealed the enrichment of the Glycolysis/Gluconeogenesis pathway in the NaLac treatment group compared to the control group (Fig. 8H). Similarly, the DEGs identified after treatment with NaLac were enriched in glycolytic and tumor-related pathways (Fig. 8I, Figure S10h). Then by intersecting the ChIP-seq data of H4K79la, RNA-seq data of cells treated with NaLac or LDH inhibitor, and TCGA-BRCA database, 51 DEGs with the enrichment of H4K79la peaks in the promoter region were identified, whose mRNA levels were decreased after treatment with the LDH inhibitor and increased after treatment with NaLac, and were also upregulated in the TCGA-BRCA database (Fig. 9A). Considering both ChIP-seq and RNA-seq data revealed enrichment in the glycolytic pathway, and the production of lactate and the lactylation of H4K79 and H4K91 were regulated by glycolysis. Among the candidate genes, LDHA, PGK1, and HK1 immediately caught our attention since they are well-known glycolysis-related genes [56–60] that are upregulated in BC tissues and whose expression was positively correlated with each other (Fig. 9B, C). Furthermore, ChIP-seq analysis revealed that H4K79la was enriched at the promoter region of LDHA, PGK1, and HK1 (Fig. 9D). RNA-seq analysis showed the expression of these glycolysis-related genes were decreased upon LDH inhibitor (Fig. 9E) treatment while increased after NaLac treatment (Figure S11a). Consequently, we decided to focus on these three genes for further research. Intriguingly, by overlapping the gene sets among the ChIP-seq of H4K91la, RNA-seq, and TCGA-BRCA database, HK1 was identified as the potential target gene of H4K91la (Figure S12a, b). Therefore, there may exist a positive feedback loop in which activated glycolysis in BC upregulated H4K79la and H4K91a, thereby promoting the transcription of glycolysis-related genes (LDHA, PGK1, and HK1). Upregulation of glycolysis-related genes can in turn stimulate glycolysis and lactylation.

**Fig. 10:**
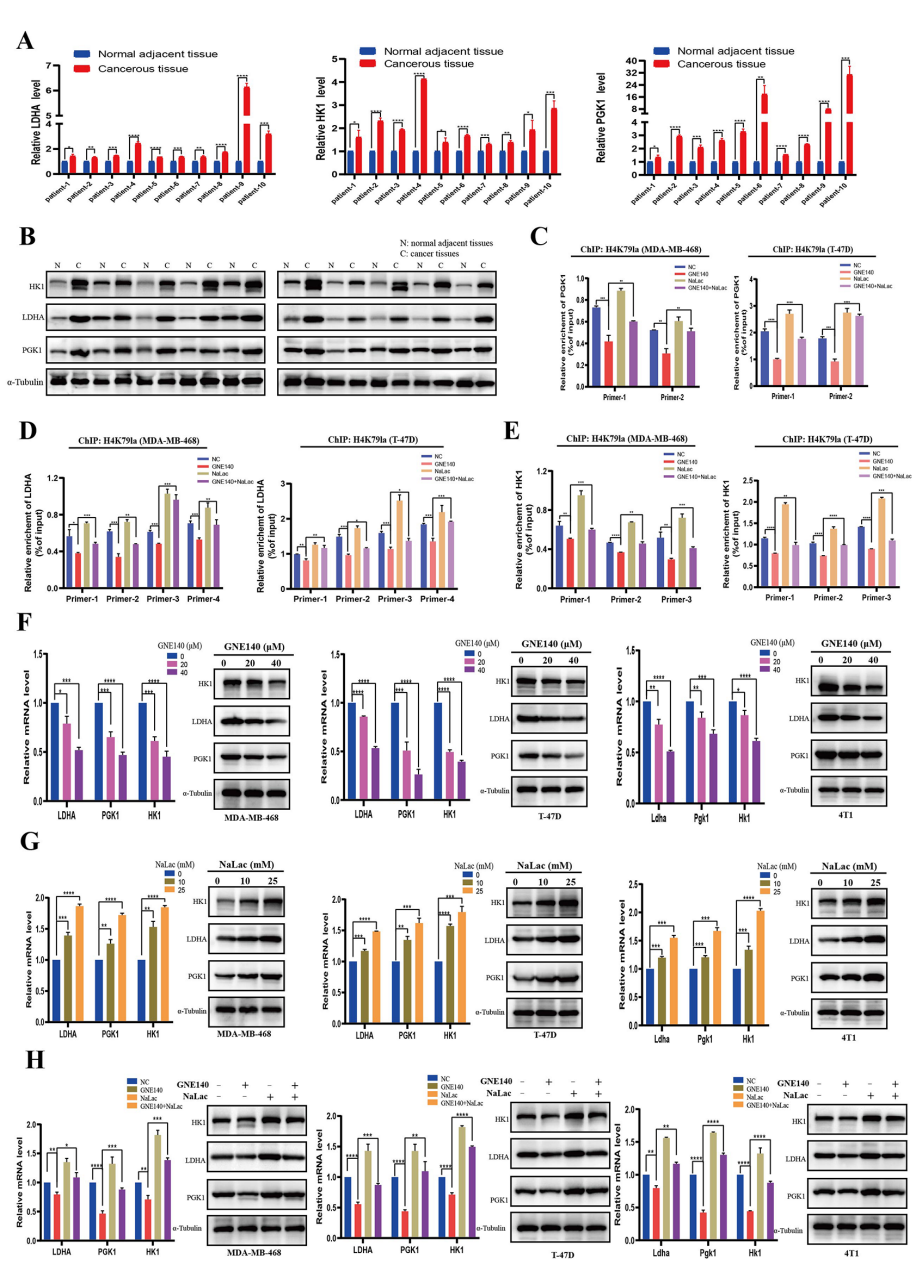
H4K79la regulated the transcription of LDHA, PGK1, and HK1. (**A**). RT-qPCR validation the mRNA expression of LDHA, HK1, and PGK1 in cancerous tissues and the normal adjacent tissues (*n* = 10). (**B**). Expression of HK1, LDHA, and PGK1 were detected in cancerous tissues and the normal adjacent tissues by Western blot (*n* = 10). (**C**). ChIP-qPCR assay of H4K79la status in the PGK1 promoter region in MDA-MB-468 and T-47D cells upon treatment with GNE140, NaLac, or GNE140 combined NaLac. (**D**). ChIP-qPCR assay of H4K79la status in the LDHA promoter region in MDA-MB-468 and T-47D cells upon treatment with GNE140, NaLac, or GNE140 combined NaLac. (**E**). ChIP-qPCR assay of H4K79la status in the HK1 promoter region in MDA-MB-468 and T-47D cells upon treatment with GNE140, NaLac, or GNE140 combined NaLac. (**F**). Expression levels of the LDHA, PGK1 and HK1 in MDA-MB-468, T-47D, and 4T1 cells treated with different concentrations of GNE140 for 24 h by RT-qPCR and Western blot. (**G**). Expression levels of the LDHA, PGK1 and HK1 in MDA-MB-468, T-47D, and 4T1 cells treated with different concentrations of NaLac for 24 h by RT-qPCR and Western blot. (**H**). Expression levels of the LDHA, PGK1 and HK1 in MDA-MB-468, T-47D, and 4T1 cells treated with GNE140, NaLac, or GNE140 combined NaLac by RT-qPCR and Western blot. Error bars represent the mean ± SD. * *P* < 0.05, ** *P* < 0.01, *** *P* < 0.001, **** *P* < 0.0001

We elucidated the synergistic regulatory effects between H4 histone lactylation and glycolysis-related genes in BC. Firstly, RT-qPCR and Western blot verified the upregulation of LDHA, PGK1, and HK1 in BC tissues compared to paired NATs (Fig. 10A-B). Moreover, ChIP-qPCR assays confirmed that H4K79la was enriched at the promoter region of LDHA, PGK1, and HK1 (Figure S11b-d). Considering that the levels of H4K79la and H4K91la were regulated by glycolysis and lactate levels (Fig. 7H-J), we conducted ChIP-qPCR assays after treating cancer cells with NaLac or LDH inhibitor. Our results revealed that the binding of H4K79la to the promoter regions of PGK1 was significantly downregulated in MDA-MB-468 and T-47D cells after treatment with the LDH inhibitor (GNE140), which was partly restored by supplementing NaLac (Fig. 10C, Figure S11e). Similarly, the reduced enrichment of H4K79la at LDHA and HK1 promoter regions upon treatment with GNE140 was compensated by adding NaLac (Fig. 10D-E, Figure S11f-g). Furthermore, ChIP-qPCR assays confirmed that H4K91la was enriched at the promoter region of HK1, and this enrichment was reduced in MDA-MB-468 and T-47D cells upon treatment with GNE140 while compensated by adding NaLac (Figure S12c-e). Consistent with the results of ChIP, GNE140 suppressed both mRNA and protein expression levels of LDHA, PGK1, and HK1 in BC cells in a dose-dependent manner (Fig. 10F). On the contrary, increased protein and mRNA levels of LDHA, PGK1, and HK1 were detected in cancer cells cultured at different concentrations of NaLac (Fig. 10G). Furthermore, adding back NaLac into GNE140-treated BC cells partly recovered the expression of LDHA, PGK1, and HK1 (Fig. 10H).

Taken together, we found a positive feedback loop between H4K79la/H4K91la/glycolytic genes and glycolysis/lactylation.

### P300 affected the lactylation of H4K79/H4K91 and subsequently regulated the expression of glycolytic genes

**Fig. 11:**
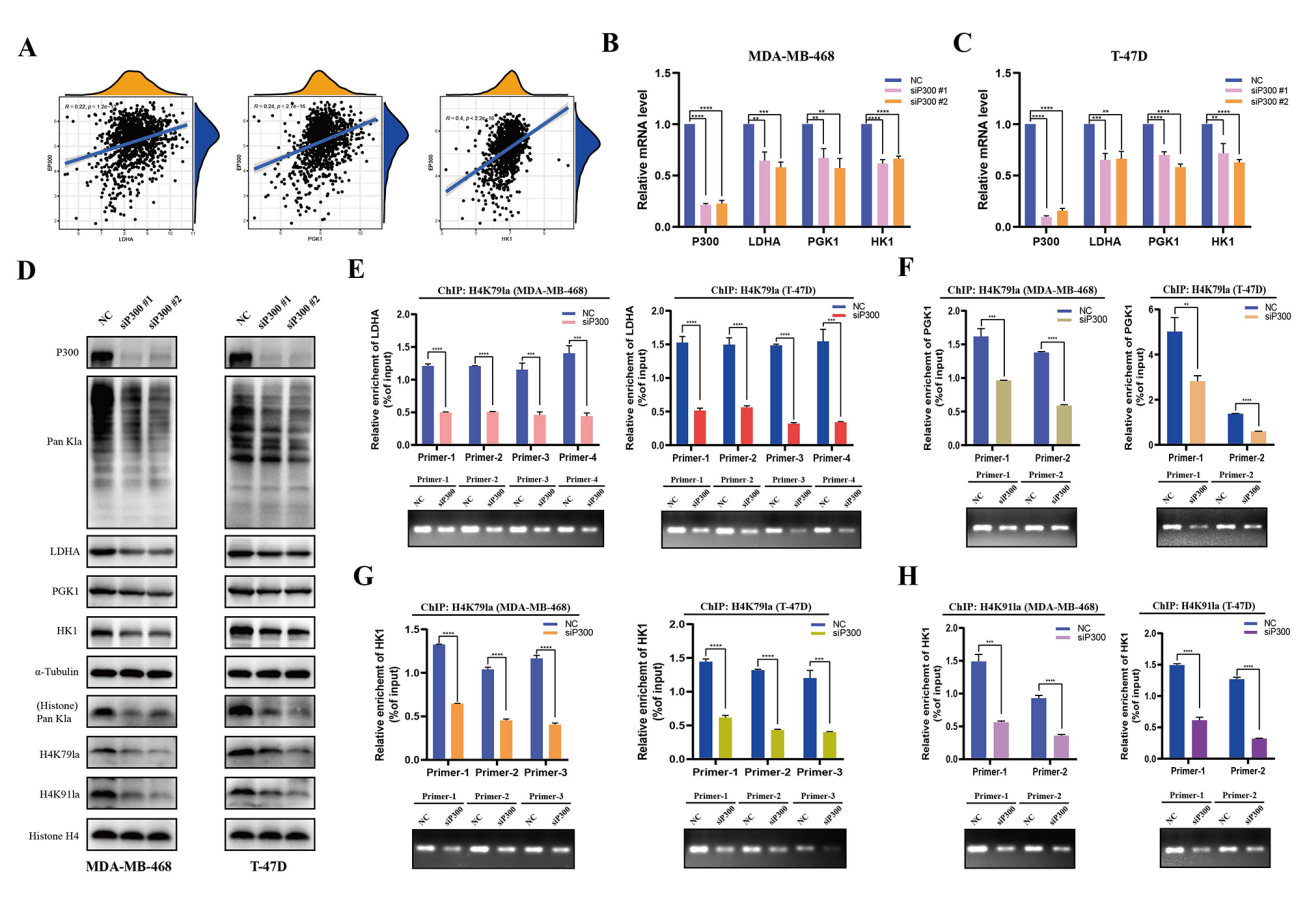
P300 is the potential writer of H4K79la and H4K91la in BC cells. (**A**). Correlation analysis of P300 expression and LDHA, PGK1 or HK1 expression in the TCGA-BRCA cohort. Significance was determined by Pearson correlation analysis. (**B**-**C**). Expression levels of the P300, LDHA, PGK1 and HK1 in MDA-MB-468 (**B**) and T-47D (**C**) cells after treated with siRNA for P300 by RT-qPCR. (**D**). Western blot analysis of P300, pan-Kla, LDHA, PGK1, HK1, H4K79la and H4K91la levels in MDA-MB-468 and T-47D cells after treated with siRNA for P300. (**E**-**G**). ChIP-qPCR assay of H4K79la status in the LDHA (**E**), PGK1 (**F**), and HK1 (**G**) promoter region in MDA-MB-468 and T-47D cells after treated with siRNA for P300. (**H**). ChIP-qPCR assay of H4K91la status in the HK1 promoter region in MDA-MB-468 and T-47D cells after treated with siRNA for P300. All data are presented as mean ± SD. * *P* < 0.05, ** *P* < 0.01, *** *P* < 0.001, **** *P* < 0.0001

Currently, several reports have shown that acetyltransferases, such as P300, KAT2A, and KAT5, most likely regulate lactylation in a lactyl-CoA-dependent manner [61, 62]. Furthermore, AARS1/2, typically catalyzes the ligation of l-alanine to transfer RNA, were recently identified as the general lysine lactyltransferase that senses lactate and mediates global lysine lacylation in tumor cells in an ATP-dependent manner [28, 63, 64]. As the newly identified modified sites in this study, the enzyme that catalyzes the lactylation of H4K79 and H4K91 is unknown. Therefore, we chose C646 (an inhibitor targeting histone acetyltransferase P300), MB-3 (targeting KAT2A), MG 149 (targeting KAT5), and Gln-AMS (targeting AARS1 and AARS2) to identify the potential writer of H4K79la and H4K91la. The results revealed that C646 obviously decreased global Kla, H4K79la, and H4K91la levels in both MDA-MB-468 and T-47D cells (Figure S13). Further correlation analysis showed positive correlations between the expression of P300 and the above-mentioned glycolytic genes (LDHA, PGK1, and HK1) (Fig. 11A). Since C646 inhibits P300 by suppressing its enzyme activity, we silenced P300 in MDA-MB-468 and T-47D cells to further verify its role in regulating lactylation levels. The results showed that the mRNA and protein levels of LDHA, PGK1, and HK1 were decreased upon siP300 (Fig. 11B-D). Meanwhile, the levels of pan-Kla, H4K79la, and H4K91la were also downregulated after treatment with siRNA for P300 (Fig. 11D), indicating that P300 can regulate histone and non-histone Kla. Consistently, ChIP-qPCR assays confirmed that the enrichment of H4K79la at the LDHA, PGK1, and HK1 promoter regions in MDA-MB-468 and T-47D cells was reduced after silencing P300 (Fig. 11E-G). Similarly, the hypo-lactylation of H4K91 upon silencing P300 decreased its binding to the promoter of HK1 (Fig. 11H). Generally, we confirmed that P300 is the potential writer of H4K79la and H4K91la and regulates the expression of glycolytic genes via histone modification.

## Discussion

As a major public health issue threatening women’s health worldwide, BC is the most prevalent malignancy and increases mortality among female patients [41, 42]. Further exploration of the molecular abnormalities underlying the development and progression of BC is urgently required for developing effective therapeutic strategies. Metabolic reprogramming is a hallmark of BC development. Cancer cells predominantly rely on aerobic glycolysis (the Warburg effect) for energy production to sustain their rapid growth and proliferation [65, 66]. Therefore, being a metabolic end-product of glycolysis in tumor cells, lactate is closely associated with the malignant behavior of BC cells [49, 50]. Recently, lactate-derived protein lactylation has been characterized as a novel PTM that epigenetically regulates gene expression in the case of histone Kla and reveals multifaceted functions in terms of non-histone Kla [18, 35, 38, 53, 54], which explains how tumor cells benefit from the Warburg effect from a new perspective. Protein lactylation has become a research hotspot since its discovery in 2019 [13]. Subsequently, several global lactylome profiles have demonstrated that Kla is a prevalent type of modification and is generally upregulated in most cancers, such as ocular melanoma [14, 29], hepatocellular carcinoma [19, 20], colorectal cancer [53], and gastric cancer [28]. However, the role of Kla in BC remained largely unclear till now. In the present study, we reported that global Kla levels were elevated in BC tissues and were associated with poor overall survival. Furthermore, we presented a global view of lactylome profile in BC and identified two previously unreported sites of histone lactylation, namely, H4K79la and H4K91la, which were hyperlactylated in BC tissues and cells. H4K79la and H4K91la were enriched at the promoters and stimulated the transcription of glycolytic genes, thereby promoting glycolysis and lactylation in BC cells (Graphical Abstract). Inhibition of lactate production with the LDH inhibitor downregulated the level of H4K79la and H4K91la, and inhibited the malignant behavior of BC cells, including their proliferation, migration, and invasion. These results revealed a crucial mechanism underlying glycolysis and lactylation in the development progression of BC.

Histones, comprised of core histones (H2A, H2B, H3, and H4) and linker histone (H1), are the fundamental structural proteins of chromosomes and frequently undergo covalent modifications at their amino residues, thereby leading to transcriptional activation or gene silencing [67–69]. Therefore, histone modifications play a vital role in regulating various pathophysiological processes and are deeply involved in the progression of numerous diseases [70–72]. With the development of mass spectrometry-based approaches, various histone acylation marks derived from cellular metabolites, such as propionylation, malonylation, butyrylation, and succinylation, were gradually discovered in recent years [73, 74]. More recently, lactate-derived histone lactylation was identified as a new type of epigenetic modification and numerous histone lactylation sites have been identified till now, such as H3K18la, H3K9la, H4K12la, and H3K56la [13]. Subsequent studies explored the role of histone lactylation in the pathogenesis of diseases, especially in cancer [14, 26, 29, 38, 53, 55]. For instance, H3K18la was shown to promote the development of ocular melanoma by regulating N1-methyladenosine modification [14]. Upregulated H3K56la is closely associated with the development and stemness of liver cancer stem cells [37]. However, the mechanism underlying histone Kla in BC remains poorly understood. Here, employing quantitative lactylproteomics analysis, increased histone Kla levels were found in tumor tissues, and numerous upregulated lactylation sites were identified in BC, including H1K96, H2AK115, H3K27, H3K56, H4K79, and H4K91. These findings are consistent with those of Yan et al.. and Duan et al.. who revealed that the lactylation levels of different histones increased globally in hepatocellular carcinoma [20] and gastrointestinal cancers [36]. Notably, among these upregulated lactylation sites, histone H4K79 and H4K91 sites caught our attention since they were hyperlactylated in both BC tissues and cells. Furthermore, there is currently a research gap on the mechanism and function of these two histone Kla sites. Thus, we focused on H4K79la and H4K91la in this study. Analogous to the research methods of other post-translational modifications at specific sites [25, 75], we developed site-specific H4K79la and H4K91la antibodies to confirm the existence and regulation of H4K79la and H4K91la. As expected, the levels of H4K79la and H4K91la were upregulated in BC and regulated by glycolysis and lactate levels. Therefore, we decided to clarify its role and regulatory mechanisms in the pathogenesis of BC.

Substantial evidence demonstrated that histone lactylation, similar to histone acetylation, functions as a vital epigenetic regulator in the transcription of many crucial genes, including those involved in macrophage polarization [13], embryonic development [76], tumorigenesis [14, 27, 38], and angiogenesis [31]. Enhanced H4K79la and H4K91la can alter the transcription pattern of corresponding genes. We conducted ChIP-seq analysis for H4K79la and H4K91la to clarify the potential biological functions of H4K79la and H4K91la in BC, and the results showed that both H4K79la and H4K91la could enrich in gene promoter regions. GO and KEGG analysis indicated that H4K79la- and H4K91la-related genes are both enriched in metabolism-related and cancer-related pathways, indicating a strong relationship between histone lactylation and biological processes. Meanwhile, RNA-seq analysis of cells treated with NaLac or the LDH inhibitor (GNE140) was also conducted, which showed that alterations in exogenous and endogenous lactate can also affect glycolytic and tumor-related pathways. Then, by overlapping the gene sets among ChIP-seq, RNA-seq, and TCGA-BRCA databases, we found that several well-known glycolysis-related genes, such as LDHA, PGK1, and HK1 were regulated by H4K79la or H4K91la. Previous studies have shown that LDHA, a crucial rate-limiting enzyme regulates the last step of glycolysis, in which pyruvate is reduced to lactate. LDHA is upregulated in BC and promotes the proliferation, migration, and invasion of cancer cells [57, 59]. Besides, PGK1 can modulate the sensitivity to paclitaxel by regulating apoptosis in BC and is significantly associated with poor prognosis [58, 60]. Additionally, HK1, the first rate-limiting enzyme of glycolysis was upregulated in BC, which may serve as a potential therapeutic target for ErbB2-overexpressing BC [56]. Our experiments in MDA-MB-468 and T-47D cells provided evidence that H4 histone lactylation regulates the transcription of glycolysis-related genes mentioned above. Notably, H4K79la promotes the expression of LDHA, PGK1, and HK1, while H4K91la promotes the expression of HK1. Since H4K79la and H4K91la rely on the presence of lactate produced in glycolysis and they promote the transcription of glycolytic genes, there may exist a positive feedback loop between histone lactylation and glycolysis. This positive feedback loop may be responsible for the persistent activation of glycolysis and enhanced lactate production in BC cells. We also showed that disruption of this feedback loop by GNE140 can downregulate H4K79la and H4K91la, and inhibit the malignant behavior of BC cells, including their proliferation, migration, and invasion. Together, our study might be an imperative supplement of current metabolic-epigenetic understandings, and the development of drugs, like GNE140, to target this glycolysis/H4K79la/H4K91la/glycolytic gene loop may provide a useful therapeutic strategy for patients with BC. Furthermore, apart from the glycolytic genes studied in this study, some other candidate genes identified in integrated analysis, such as Nectin cell adhesion molecule 4 (NECTIN4), Kringle-containing transmembrane protein 2 (KREMEN2), Serpin family-B member 7 (SERPINB7), and basal cell adhesion molecule (BCAM), act as oncogenes during cancer progression [77–81], which remain to be further studied in BC.

Metabolic reprogramming and epigenetic modification are closely linked and regulate each other in a reciprocal manner [1, 2]. The glycolysis/H4K79la/H4K91la/glycolytic genes positive feedback identified in the present study provides novel insight into the crosstalk between metabolic reprogramming and epigenetic modification. Consistently, other studies indicated that Kla serves as a feedback regulator of metabolism by affecting the transcription and activity of metabolism-related enzymes, especially those involved in glycolysis [16, 23, 27, 82–84]. For instance, the accumulation of lactate in colorectal cancer cells not only activates the transcription of NSUN2, a 5-methylcytosine (m^5^C) methyltransferase, through H3K18la but also induces the lactylation of NSUN2 at the Lysine 356 residue. This mechanism is crucial for modulating the expression of ENO1 in an m^5^C-dependent manner, resulting in increased glycolysis and lactate production, thereby forming a positive feedback loop involving the NSUN2/m5C/ENO1 signaling axis to promote the progression of colorectal cancer [84, 85]. Furthermore, elevated lactylation of H4K21 in brain microglial cells resulted in the transcription of the glycolysis gene PKM2 and activated the glycolysis/H4K12la/PKM2 positive feedback loop, which exacerbated the dysfunction in Alzheimer’s disease [16]. Additionally, PKM2 acted as a lactylation substrate and lactylated at the Lysine 62 residue, which inhibited its tetramer-to-dimer transition and promoted the transition of pro-inflammatory macrophages toward a reparative phenotype [23]. Altogether, lactate, the substrate for lactylation, is also a metabolic end-product of glycolysis. Hence, it is challenging to determine whether protein lactylation is a driving factor or a consequence of metabolic reprogramming. Therefore, a deeper insight into the nature of lactylation is still needed.

Similar to other PTMs, protein lactylation relies on a series of lactyltransferases and delactylases [13, 19, 32, 69, 82]. P300, a crucial acetyltransferase, is also a lactyltransferase responsible for histone lactylation [13]. Defining the regulatory role of lactylation by P300 in BC, we showed that the expression of P300 was positively associated with LDHA, PGK1, and HK1 in the TCGA-BRCA database. Notably, C646 (P300 inhibitor) treatment or silencing P300 decreased global Kla, H4K79la, and H4K91la levels, silencing P300 suppressed the transcription of the downstream glycolytic genes. Generally, we confirmed that P300 is the potential writer of H4K79la and H4K91la. Further studies on the exact chemical reactions involved in H4K79la and H4K91la are still remains to be performed.

Despite the novel insights provided in this study, there are several limitations needs further consideration. Firstly, regarding H4K79la and H4K91la, histone H4 is encoded by numerous genes [86]. Given the complex and dynamic nature of histone H4 modifications [87], we cannot efficiently generate site mutations for histone H4, and there is currently lacking inhibitors that targeting specific histone lactylation sites. Therefore, we could not investigate the specific effects of H4K79la and H4K91la on the positive feedback loop. Secondly, our protein lactylome analysis of BC tissues and cells revealed that lactylated proteins were widespread in several subcellular compartments and were closely associated with various cancer-related biological processes. Therefore, apart from histone lactylation investigated in this study, the underlying mechanisms of non-histone lactylation in BC progression are worth further investigation. Furthermore, lactate has been shown to promote tumor progression by inducing an immunosuppressive microenvironment [66]. Recently, lactate-derived kla has been implicated in the regulation of the immune microenvironment [15, 25, 38], considering the accumulation of lactate in the tumor microenvironment, Kla levels and the role of Kla in immune cells in BC tissues are worth attention. In addition, uncovering the crosstalk between lactylation and other PTMs is a complex research focus. Particularly, the relationship between lactylation and acetylation is intricate, as they both affect lysine residues and are regulated by a set of shared enzymes. The substrates for these two modifications are generated from opposite metabolic reactions starting from pyruvate. Therefore, they may play antagonistic roles in various biological processes, which deserve further studies.

## Conclusions

In summary, our study revealed protein lactylation was upregulated in BC and inhibition of protein lactylation suppressed the development and progression of BC. For the first time, we identified two lactylation modification sites in histone H4, which forms a glycolysis/H4K79la/H4K91la/glycolytic genes positive feedback loop to enhance glycolysis and lactylation, thereby providing novel insights into the crosstalk between metabolic reprogramming and epigenetic modification. Furthermore, we presented an initial landscape of the lactylome profile in BC and highlighted numerous differentially lactylated proteins that are worthy of further investigation. Together, these findings expand our understanding of the regulatory mechanisms of Kla in the progression of BC and establish the fundamental knowledge for further research, offering promising treatments for cancer.

## Supplementary Information

Below is the link to the electronic supplementary material.

Supplementary Material 1: Figure S1. BC tissues are active in glycolysis/serum lactate-related pathways. (a-e). GESA plots evaluating glycolytic glycolysis/serum lactate related pathways changes in BC tissues (T group) and normal controls (N group) based on TCGA-BRCA database. (f). Kaplan-Meier analysis and log-rank tests of disease-free survival in BC patients with low (n=101) and high pan-Kla (n=133) levels. (g). Representative pictures of IHC staining of lactylation levels in lymph node metastasis and non-metastasis BC tissues. Scale bars: 100µm (10x); 50µm (20x). (h,i). Represent pictures and statistical results of lactyation levels in different histological grade of BC tissues by IHC staining. Scale bars: 100µm (10x); 50µm (20x).

Supplementary Material 2: Figure S2. Effects of increased protein lactyation on the proliferative capacity of BC cells. (a). Cell proliferation ability was evaluated by CCK8 assays in MDA-MB-468, MCF7, T-47D, and 4T1 cells after treatment with 0mM, 10mM or 25mM of NaLac. (b). Cell proliferation ability was evaluated by colony formation assays in MDA-MB-468, MCF7, T-47D, and 4T1 cells after treatment with different concentrations of NaLac. Error bars represent the mean±SD, ns: not significant.

Supplementary Material 3: Figure S3. Effects of increased protein lactyation on the migration and invasion ability of BC cells. (a). Cell migration ability was evaluated by wounding healing assay in MDA-MB-468 and MCF7 cells at different concentrations of NaLac-treatment group, the wound space was photographed at 0, 24 and 48h. (b). Cell migration ability was evaluated by wounding healing assay in T-47D and 4T1 cells in different concentrations at NaLac-treatment group. (c). Cell invasion ability was evaluated by transwell assays in MDA-MB-468 cell after treatment with different concentrations of NaLac. (d). Cell invasion ability was evaluated by transwell assays in T-47D and 4T1 cells after treatment with different concentrations of NaLac. Error bars represent the mean±SD, ns: not significant. Scale bars: 200µm.

Supplementary Material 4: Figure S4. Silencing LDHA/LDHB reduced global Kla levels and inhibited the proliferative capacity of BC cells. (a). qRT-PCR analysis the relative mRNA levels of LDHA and LDHB after transfected with siLDHA or siLDHB. (b). Western blot analysis of Kla and LDHA in MDA-MB-468, MCF7, and T-47D cells after transfected with siLDHA. (c). Western blot analysis of Kla and LDHB in MDA-MB-468, MCF7, and T-47D cells after transfected with siLDHB. (d-e). Proliferation capacity of MDA-MB-468 (d) and T-47D cells (e) after transfected with siLDHA were analyzed by CCK8 and colony formation assays. (f-g). Proliferation capacity of MDA-MB-468 (f) and T-47D cells (g) after transfected with siLDHB were analyzed by CCK8 and colony formation assays. Relative cell numbers are shown as means ± SD.* P < 0.05, ** P < 0.01, *** P < 0.001, **** P < 0.0001.

Supplementary Material 5: Figure S5. Silencing LDHA/LDHB inhibited the migration and invasion ability of BC cells. (a). Migration ability of T-47D and MDA-MB-468 cells after transfected with siLDHA were analyzed by wounding healing assay, the wound space was photographed at 0, 24 and 48h. (b-c). Invasion ability of T-47D (b) and MDA-MB-468 cells (c) after transfected with siLDHA were analyzed by transwell assay. (d). Migration ability of MDA-MB-468 and T-47D cells after transfected with siLDHB were analyzed by wounding healing assay, the wound space was photographed at 0, 24 and 48h. (e-f). Invasion ability of MDA-MB-468 (e) and T-47D cells (f) after transfected with siLDHB were analyzed by transwell assay. Relative cell numbers are shown as means ± SD.* P < 0.05, ** P < 0.01, *** P < 0.001, **** P < 0.0001. Scale bars: 200µm.

Supplementary Material 6: Figure S6. GNE140 inhibited the proliferation, migration, and invasion of BC cells in a concentration-dependent manner. (a,b). Cell proliferation ability was evaluated by CCK8 (a) and colony formation (b) assays in MDA-MB-468 and T-47D cells after treatment with different concentrations of GNE140. (c). Cell migration ability was evaluated by wounding healing assay in MDA-MB-468 and T-47D cells after treatment with different concentrations of GNE140, the wound space was photographed at 0, 24 and 48h. Scale bars: 200µm. (d). Cell invasion ability was evaluated by transwell assays in MDA-MB-468 and T-47D cells after treatment with different concentrations of GNE140. Scale bars: 200µm. Error bars represent the mean±SD. * P < 0.05, ** P < 0.01, *** P < 0.001, **** P < 0.0001.

Supplementary Material 7: Figure S7. Characterization of the lactylome in BC tissues and cells. (a). Summary of identified lactylation sites and the total proteins filtered through database retrieval. (b). Bar charts displaying upregulated and downregulated modification sites and proteins between cancer cells (MCF7, T-47D, and MDA-MB-468) and normal mammary epithelial cell line (MCF10A). (c). Venn diagram of common DELPs identified in BC cells compared to normal mammary epithelial cell. (d). Subcellular localization of common DELPs identified in BC cells. GO-CC (e) and GO-MF (f) enrichment analysis of common DELPs identified in BC cells. (g). Bar charts displaying statistical data on differential proteins and modification sites in tumor tissues versus normal tissues. (h). Subcellular localization of DELPs identified in in tumor versus normal tissues. GO-CC (i) and GO-MF (j) enrichment analysis of DELPs identified in in tumor versus normal tissues.

Supplementary Material 8: Figure S8. Verification data of the specialized H4K79la and H4K91la antibodies. (a-b). Histogram indicating upregulated lactylation of H4K79 and H4K91 in cancer cells (MCF7, T-47D, and MDA-MB-468) versus normal mammary epithelial cell (MCF 10A). (c). Verification of the specificity of the H4K79la and H4K91la antibody by dot blots experiment. (d-f). The verification of two modified peptides and one non-modified peptide designed for H4K79 by mass spectrometry. (d). Lactyl-H4K79 peptide 1: CEHAKR-(lactyl)K-TVTAM. (e). Lactyl-H4K79 peptide 2: CTEHAKR-(lactyl)K-TVTAMD. (f). unmodified-H4K79 peptide: CTEHAKRKTVTAMD. (g-i). The verification of two modified peptides and one non-modified peptide designed for H4K91 by mass spectrometry. (g). Lactyl-H4K91 peptide 1: CDVVYAL-(lactyl)K-RQGR. (h). Lactyl-H4K91 peptide 2: CMDVVYAL-(lactyl)K-RQGRT. (i). unmodified-H4K91 peptide: CMDVVYALKRQGRT.

Supplementary Material 9: Figure S9. H4K79la and H4K91la levels were significantly upregulated in BC and regulated by glycolysis and lactate levels. (a). Western blot analysis of H4K79la and H4K91la levels in BC tissues and paired normal adjacent tissues. (b). Representative IHC images and statistical results of H4K79la levels in BC tissues and normal tissues. Scale bars: 100µm (10x); 50µm (20x). (c). Representative IHC images and statistical results of H4K91la levels in BC tissues and normal tissues. Scale bars: 100µm (10x); 50µm (20x). (d). Western blot analysis of H4K79la and H4K91la levels in T-47D and 4T1 cells cultured in different concentrations of NaLac. (e). Western blot analysis of H4K79la and H4K91la levels in T-47D and 4T1 cells treated with different doses of GNE140. (f) Western blot analysis of H4K79la and H4K91la levels in T-47D and 4T1 cells treated with GNE140 then incubated with or without NaLac.

Supplementary Material 10: Figure S10. GO and KEGG analysis of ChIP-seq and RNA-seq datasets. (a-b). Motif sequence analysis of H4K79la (a) and H4K91la (b) peaks. (c). KEGG analysis of H4K91la-realted promoter peak genes. (d). GO analysis of H4K91la-realted promoter peak genes. (e). GO analysis of differentially expressed genes after GNE140 treatment. (f). Volcano plot of differently expressed genes in GNE140-treated group based on RNA-seq. (g). Volcano plot of differently expressed genes in NaLac-treated group based on RNA-seq. (h). GO analysis of differentially expressed genes after NaLac treatment.

Supplementary Material 11: Figure S11. H4K79la activated the transcription of LDHA, PGK1, and HK1. (a). Representative IGV tracks showing increased PGK1, LDHA, and HK1 expression upon NaLac treatment by RNA-seq. (b-d). DNA fragments from MDA-MB-468 cells were immunoprecipitated with the H4K79la-specific antibody and analyzed by qPCR using the indicated primers. (b). Verification of H4K79la enriched at PGK1 promoter region by specialized PGK1 primers. (c). Verification of H4K79la enriched at LDHA promoter region by specialized LDHA primers. (d). Verification of H4K79la enriched at HK1 promoter region by specialized HK1 primers. (e). Agarose gel electrophoresis corresponding to Figure 10C. (f). Agarose gel electrophoresis corresponding to Figure 10D. (g). Agarose gel electrophoresis corresponding to Figure 10E.

Supplementary Material 12: Figure S12. H4K91la activated the transcription of HK1. (a). Combination of ChIP-seq, RNA-seq and TCGA-BRCA database to identify the potential downstream targets of H4K91la. (b). Representative IGV tracks showing enriched H4K91la modification in HK1 promotor regions by ChIP-seq. Arrows are the H4K91la peaks at the gene promotor. (c). DNA fragments from MDA-MB-468 cells were immunoprecipitated with the H4K91la-specific antibody and analyzed by qPCR using the specialized HK1 primers. (d-e). ChIP-qPCR assay of H4K91la status in the HK1 promoter region in MDA-MB-468 (d) and T-47D (e) cells upon treatment with GNE140, NaLac, or GNE140 combined NaLac. Error bars represent the mean±SD. * P < 0.05, ** P < 0.01, *** P < 0.001, **** P < 0.0001.

Supplementary Material 13: Figure S13. P300 is the potential writer of H4K79la and H4K91la in BC cells. (a, b). Kla levels of MDA-MB-468 cell (a) and T-47D (b) cells treated with different inhibitors for 24h. C646 (20µm), MB-3 (30µm), MG 149 (20µm), and Gln-AMS (10µm). (c, d). H4K79la and H4K91la levels of MDA-MB-468 cell (c) and T-47D (d) cells treated with different inhibitors for 24h.

Supplementary Material 14: Table S1. Clinical characteristics data.

Supplementary Material 15: Table S2. siRNA sequences and primer sequences were used in this study.

Supplementary Material 16: Table S3. Analysis of the correlation between Kla level and clinicopathological characteristics in BC patients.

Supplementary Material 17: Table S4. The list of lactylation sites identified in BC.

Supplementary Material 18: Table S5. Functional enrichment analysis of differentially expressed lactylated proteins.

Supplementary Material 19: Table S6. The list of differentially expressed genes after BC cell treated with LDH inhibitor.

Supplementary Material 20: Table S7. The list of differentially expressed genes after BC cell treated with NaLac.

Supplementary Material 21: Table S8. H4K79la top100 for motif.

Supplementary Material 22: Table S9. H4K91la top100 for motif.

Supplementary Material 23

## Data Availability

No datasets were generated or analysed during the current study.
