# Supplementary figures and images for: H4K79 and H4K91 histone lactylation, newly identified lactylation sites enriched in breast cancer
Source: J Exp Clin Cancer Res. 2025 Aug 23;44:252. doi: 10.1186/s13046-025-03512-6 (PMC12374308; doi:10.1186/s13046-025-03512-6)

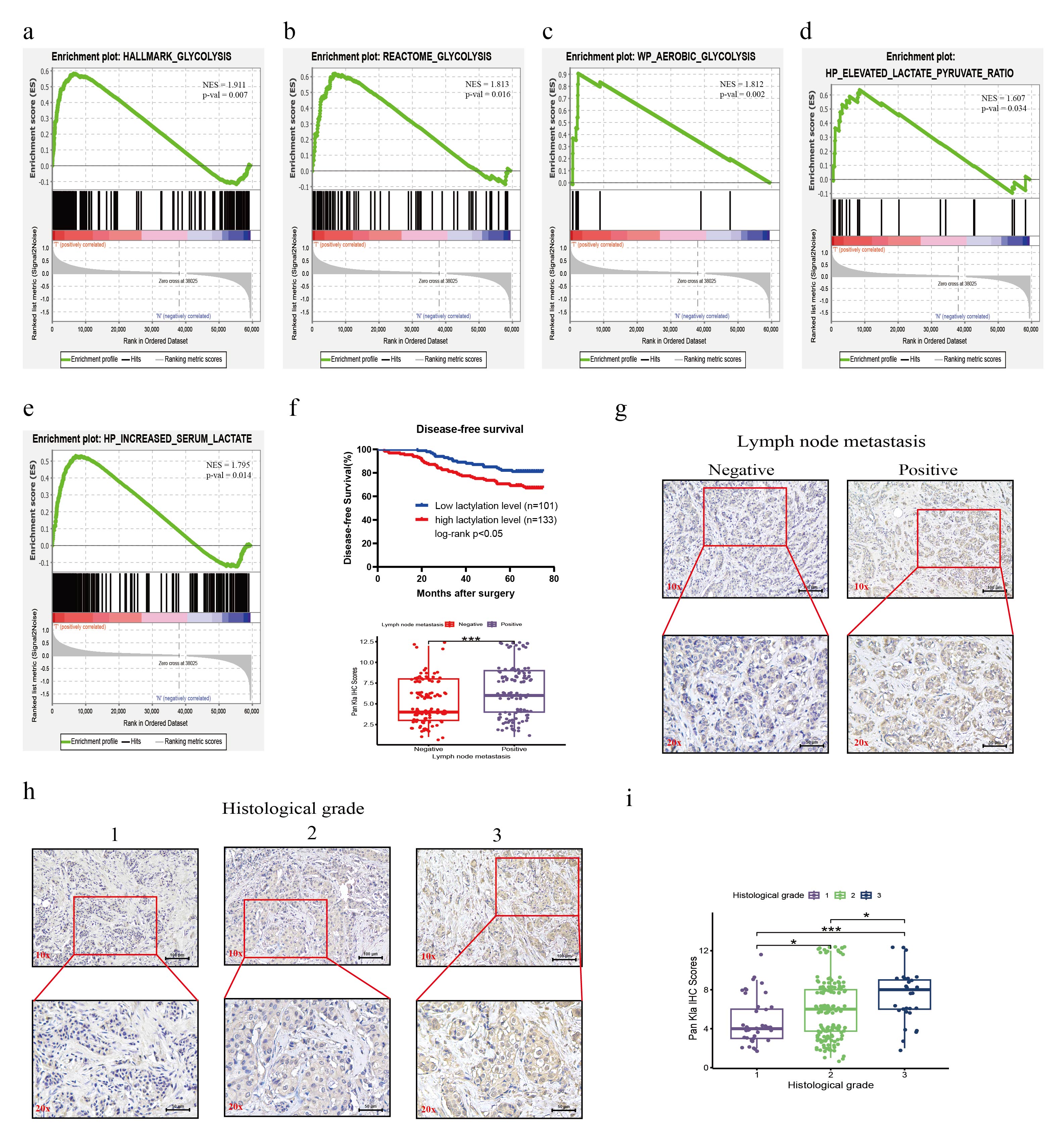

Supplement: Supplementary file 1 — Supplementary Material 1: Figure S1. BC tissues are active in glycolysis/serum lactate-related pathways. (a-e). GESA plots evaluating glycolytic glycolysis/serum lactate related pathways changes in BC tissues (T group) and normal controls (N group) based on TCGA-BRCA database. (f). Kaplan-Meier analysis and log-rank tests of disease-free survival in BC patients with low (n=101) and high pan-Kla (n=133) levels. (g). Representative pictures of IHC staining of lactylation levels in lymph node metastasis and non-metastasis BC tissues. Scale bars: 100µm (10x); 50µm (20x). (h,i). Represent pictures and statistical results of lactyation levels in different histological grade of BC tissues by IHC staining. Scale bars: 100µm (10x); 50µm (20x). [file 13046_2025_3512_MOESM1_ESM.jpg]

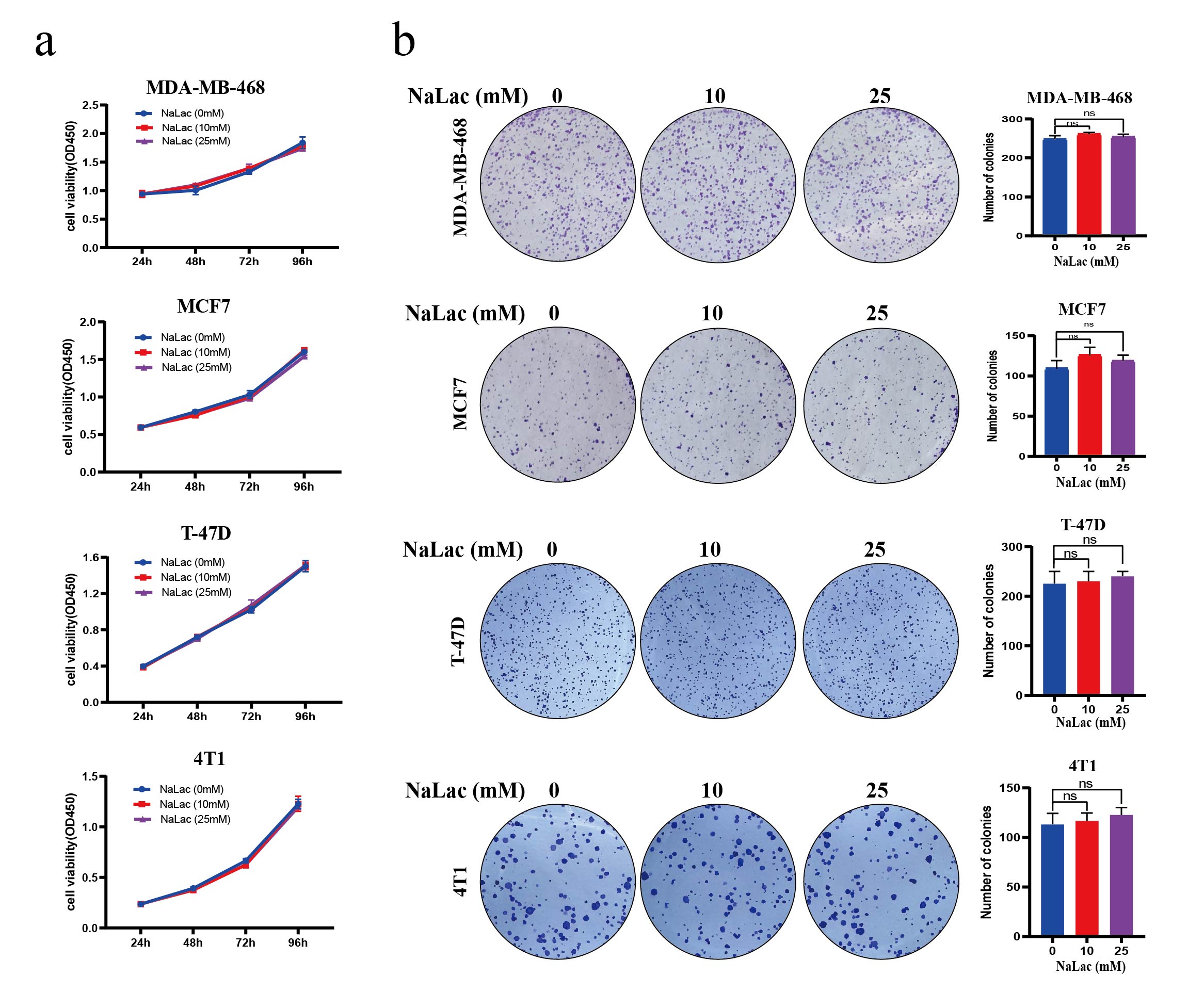

Supplement: Supplementary file 2 — Supplementary Material 2: Figure S2. Effects of increased protein lactyation on the proliferative capacity of BC cells. (a). Cell proliferation ability was evaluated by CCK8 assays in MDA-MB-468, MCF7, T-47D, and 4T1 cells after treatment with 0mM, 10mM or 25mM of NaLac. (b). Cell proliferation ability was evaluated by colony formation assays in MDA-MB-468, MCF7, T-47D, and 4T1 cells after treatment with different concentrations of NaLac. Error bars represent the mean±SD, ns: not significant. [file 13046_2025_3512_MOESM2_ESM.jpg]

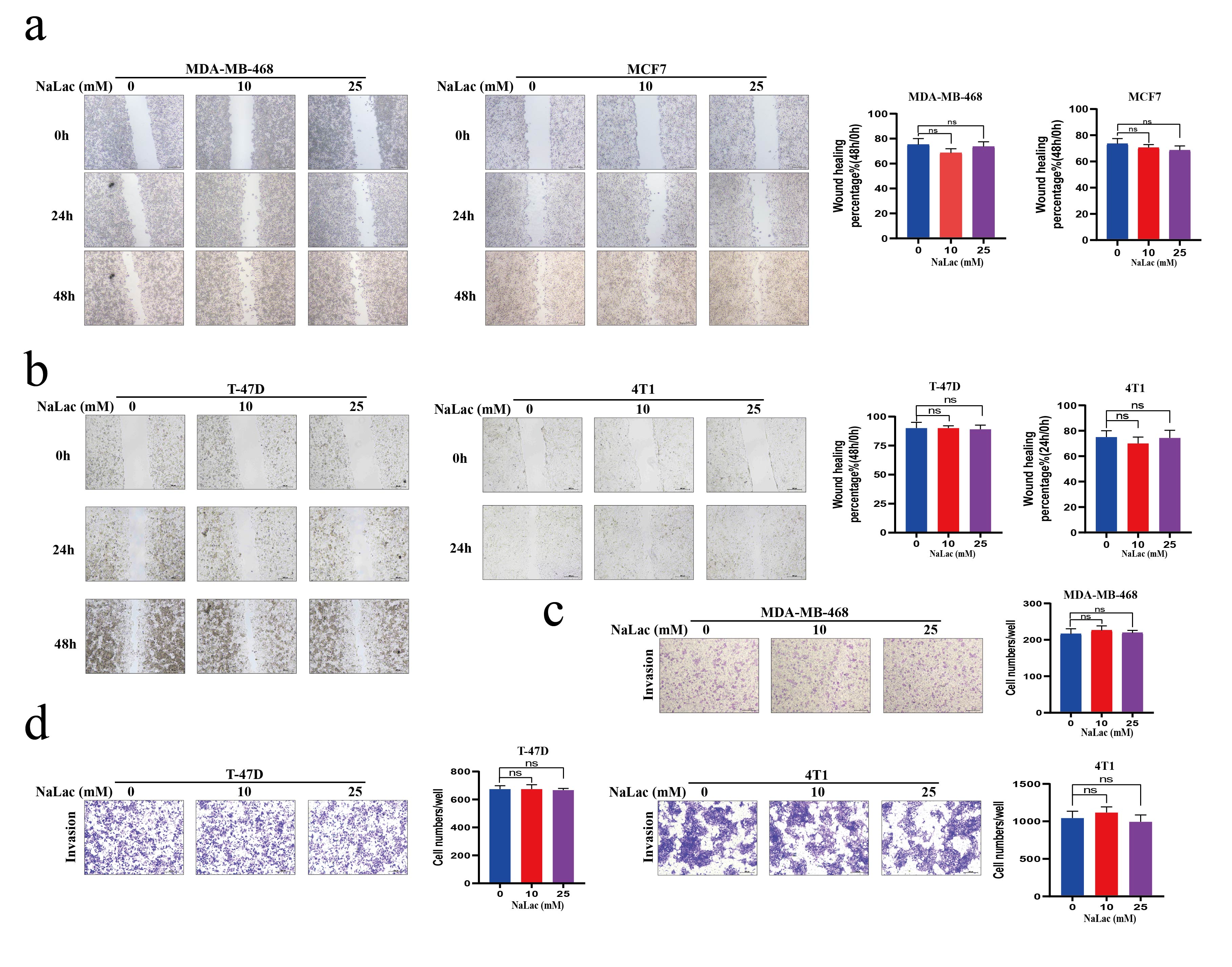

Supplement: Supplementary file 3 — Supplementary Material 3: Figure S3. Effects of increased protein lactyation on the migration and invasion ability of BC cells. (a). Cell migration ability was evaluated by wounding healing assay in MDA-MB-468 and MCF7 cells at different concentrations of NaLac-treatment group, the wound space was photographed at 0, 24 and 48h. (b). Cell migration ability was evaluated by wounding healing assay in T-47D and 4T1 cells in different concentrations at NaLac-treatment group. (c). Cell invasion ability was evaluated by transwell assays in MDA-MB-468 cell after treatment with different concentrations of NaLac. (d). Cell invasion ability was evaluated by transwell assays in T-47D and 4T1 cells after treatment with different concentrations of NaLac. Error bars represent the mean±SD, ns: not significant. Scale bars: 200µm. [file 13046_2025_3512_MOESM3_ESM.jpg]

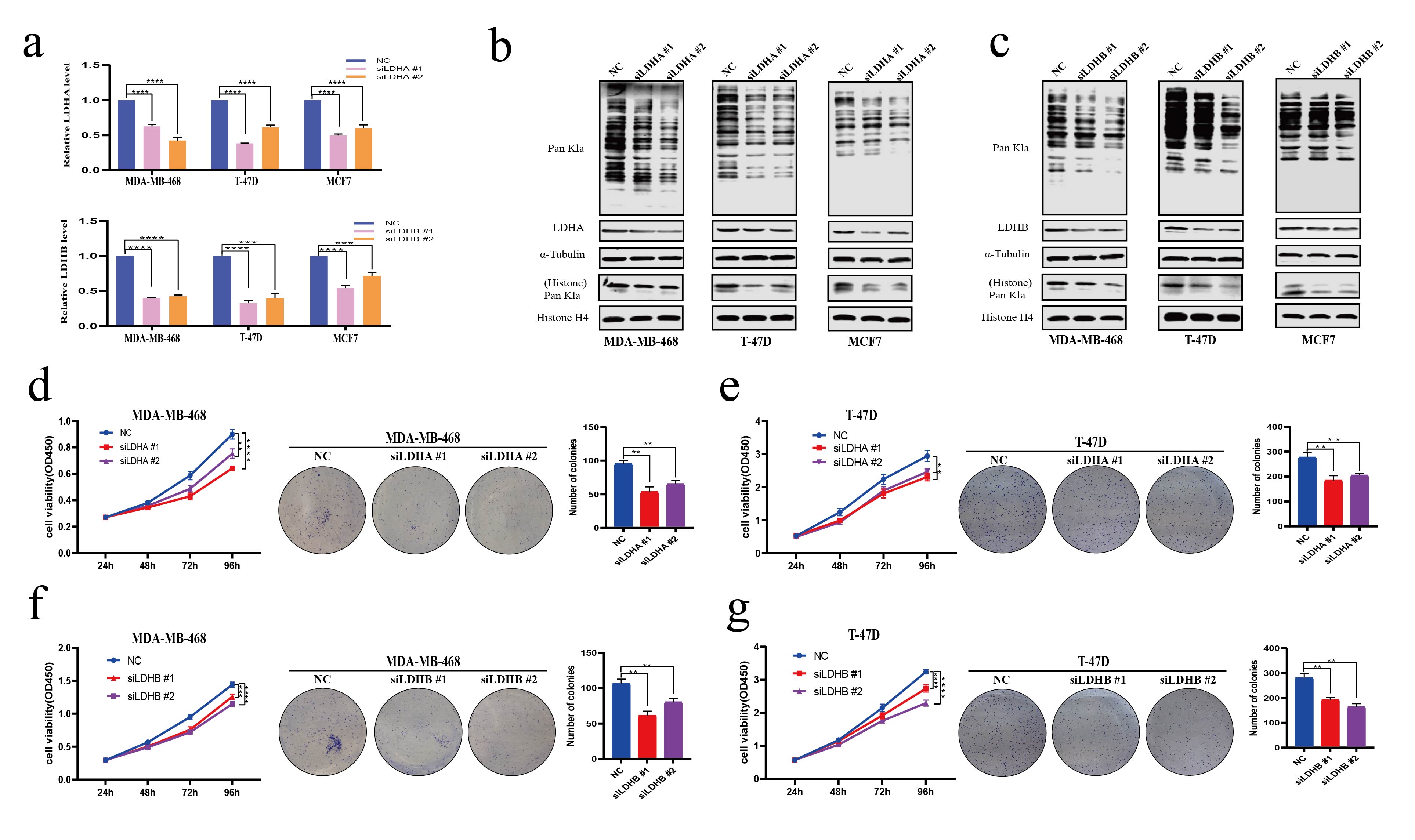

Supplement: Supplementary file 4 — Supplementary Material 4: Figure S4. Silencing LDHA/LDHB reduced global Kla levels and inhibited the proliferative capacity of BC cells. (a). qRT-PCR analysis the relative mRNA levels of LDHA and LDHB after transfected with siLDHA or siLDHB. (b). Western blot analysis of Kla and LDHA in MDA-MB-468, MCF7, and T-47D cells after transfected with siLDHA. (c). Western blot analysis of Kla and LDHB in MDA-MB-468, MCF7, and T-47D cells after transfected with siLDHB. (d-e). Proliferation capacity of MDA-MB-468 (d) and T-47D cells (e) after transfected with siLDHA were analyzed by CCK8 and colony formation assays. (f-g). Proliferation capacity of MDA-MB-468 (f) and T-47D cells (g) after transfected with siLDHB were analyzed by CCK8 and colony formation assays. Relative cell numbers are shown as means ± SD.* P < 0.05, ** P < 0.01, *** P < 0.001, **** P < 0.0001. [file 13046_2025_3512_MOESM4_ESM.jpg]

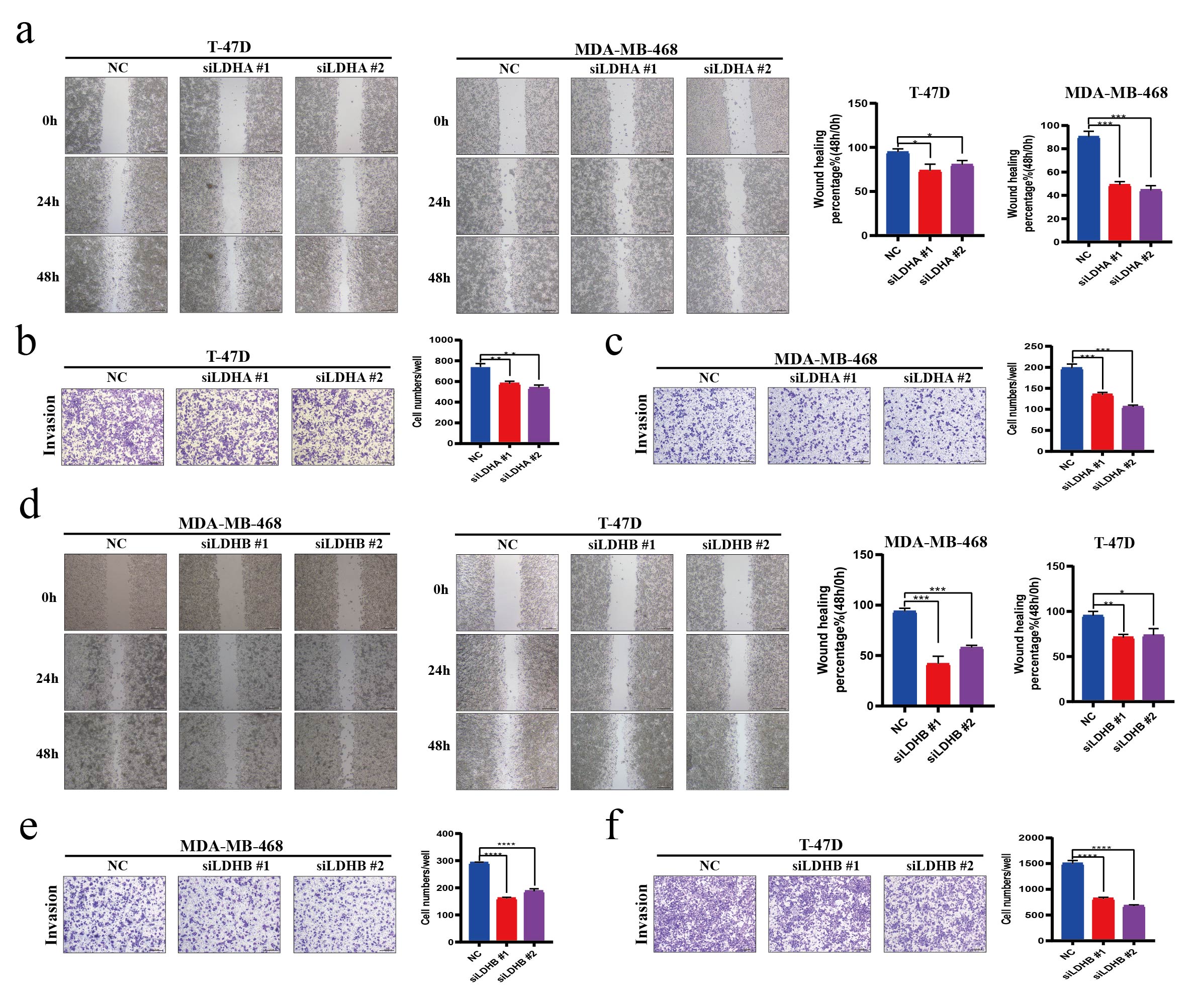

Supplement: Supplementary file 5 — Supplementary Material 5: Figure S5. Silencing LDHA/LDHB inhibited the migration and invasion ability of BC cells. (a). Migration ability of T-47D and MDA-MB-468 cells after transfected with siLDHA were analyzed by wounding healing assay, the wound space was photographed at 0, 24 and 48h. (b-c). Invasion ability of T-47D (b) and MDA-MB-468 cells (c) after transfected with siLDHA were analyzed by transwell assay. (d). Migration ability of MDA-MB-468 and T-47D cells after transfected with siLDHB were analyzed by wounding healing assay, the wound space was photographed at 0, 24 and 48h. (e-f). Invasion ability of MDA-MB-468 (e) and T-47D cells (f) after transfected with siLDHB were analyzed by transwell assay. Relative cell numbers are shown as means ± SD.* P < 0.05, ** P < 0.01, *** P < 0.001, **** P < 0.0001. Scale bars: 200µm. [file 13046_2025_3512_MOESM5_ESM.jpg]

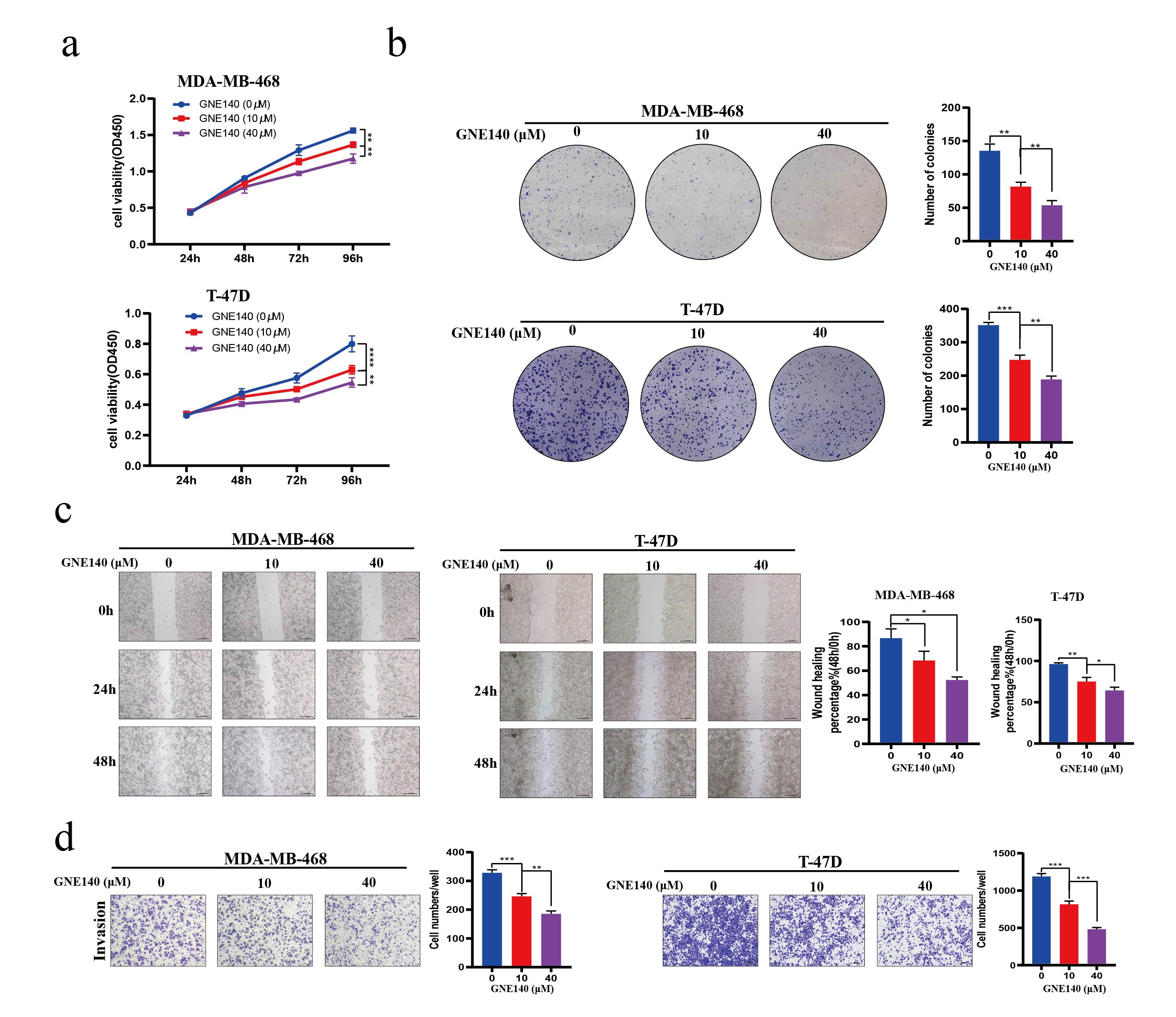

Supplement: Supplementary file 6 — Supplementary Material 6: Figure S6. GNE140 inhibited the proliferation, migration, and invasion of BC cells in a concentration-dependent manner. (a,b). Cell proliferation ability was evaluated by CCK8 (a) and colony formation (b) assays in MDA-MB-468 and T-47D cells after treatment with different concentrations of GNE140. (c). Cell migration ability was evaluated by wounding healing assay in MDA-MB-468 and T-47D cells after treatment with different concentrations of GNE140, the wound space was photographed at 0, 24 and 48h. Scale bars: 200µm. (d). Cell invasion ability was evaluated by transwell assays in MDA-MB-468 and T-47D cells after treatment with different concentrations of GNE140. Scale bars: 200µm. Error bars represent the mean±SD. * P < 0.05, ** P < 0.01, *** P < 0.001, **** P < 0.0001. [file 13046_2025_3512_MOESM6_ESM.jpg]

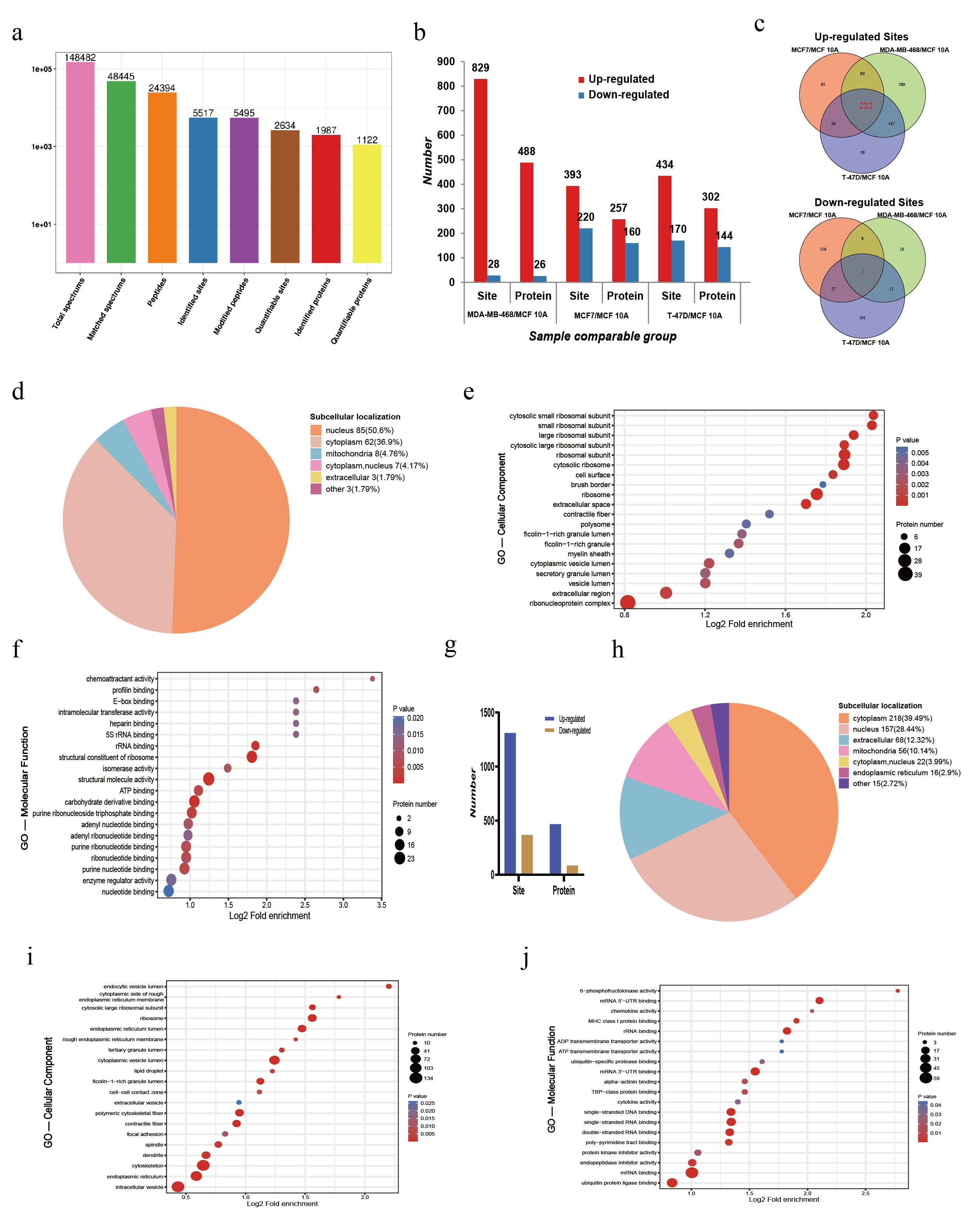

Supplement: Supplementary file 7 — Supplementary Material 7: Figure S7. Characterization of the lactylome in BC tissues and cells. (a). Summary of identified lactylation sites and the total proteins filtered through database retrieval. (b). Bar charts displaying upregulated and downregulated modification sites and proteins between cancer cells (MCF7, T-47D, and MDA-MB-468) and normal mammary epithelial cell line (MCF10A). (c). Venn diagram of common DELPs identified in BC cells compared to normal mammary epithelial cell. (d). Subcellular localization of common DELPs identified in BC cells. GO-CC (e) and GO-MF (f) enrichment analysis of common DELPs identified in BC cells. (g). Bar charts displaying statistical data on differential proteins and modification sites in tumor tissues versus normal tissues. (h). Subcellular localization of DELPs identified in in tumor versus normal tissues. GO-CC (i) and GO-MF (j) enrichment analysis of DELPs identified in in tumor versus normal tissues. [file 13046_2025_3512_MOESM7_ESM.jpg]

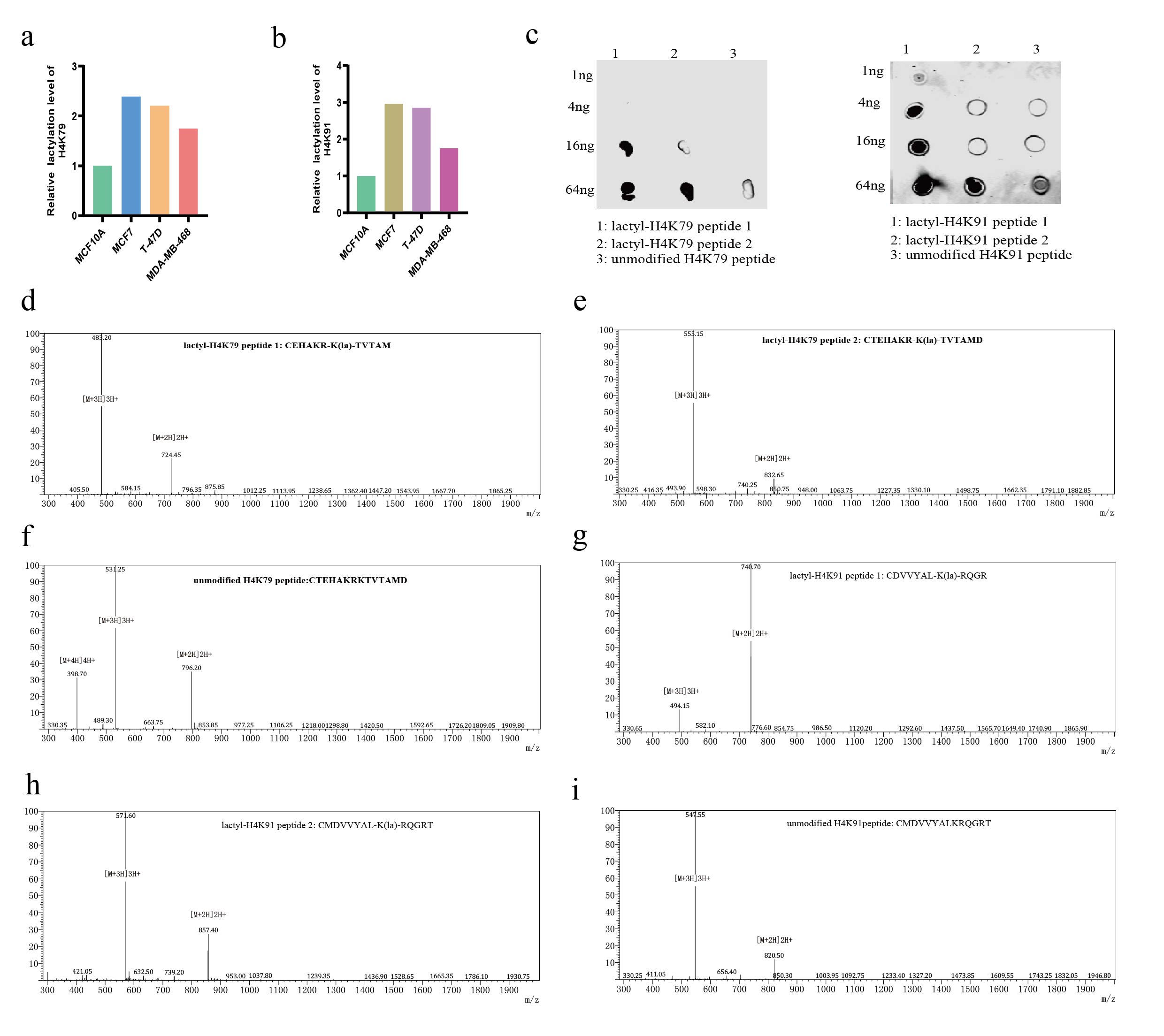

Supplement: Supplementary file 8 — Supplementary Material 8: Figure S8. Verification data of the specialized H4K79la and H4K91la antibodies. (a-b). Histogram indicating upregulated lactylation of H4K79 and H4K91 in cancer cells (MCF7, T-47D, and MDA-MB-468) versus normal mammary epithelial cell (MCF 10A). (c). Verification of the specificity of the H4K79la and H4K91la antibody by dot blots experiment. (d-f). The verification of two modified peptides and one non-modified peptide designed for H4K79 by mass spectrometry. (d). Lactyl-H4K79 peptide 1: CEHAKR-(lactyl)K-TVTAM. (e). Lactyl-H4K79 peptide 2: CTEHAKR-(lactyl)K-TVTAMD. (f). unmodified-H4K79 peptide: CTEHAKRKTVTAMD. (g-i). The verification of two modified peptides and one non-modified peptide designed for H4K91 by mass spectrometry. (g). Lactyl-H4K91 peptide 1: CDVVYAL-(lactyl)K-RQGR. (h). Lactyl-H4K91 peptide 2: CMDVVYAL-(lactyl)K-RQGRT. (i). unmodified-H4K91 peptide: CMDVVYALKRQGRT. [file 13046_2025_3512_MOESM8_ESM.jpg]

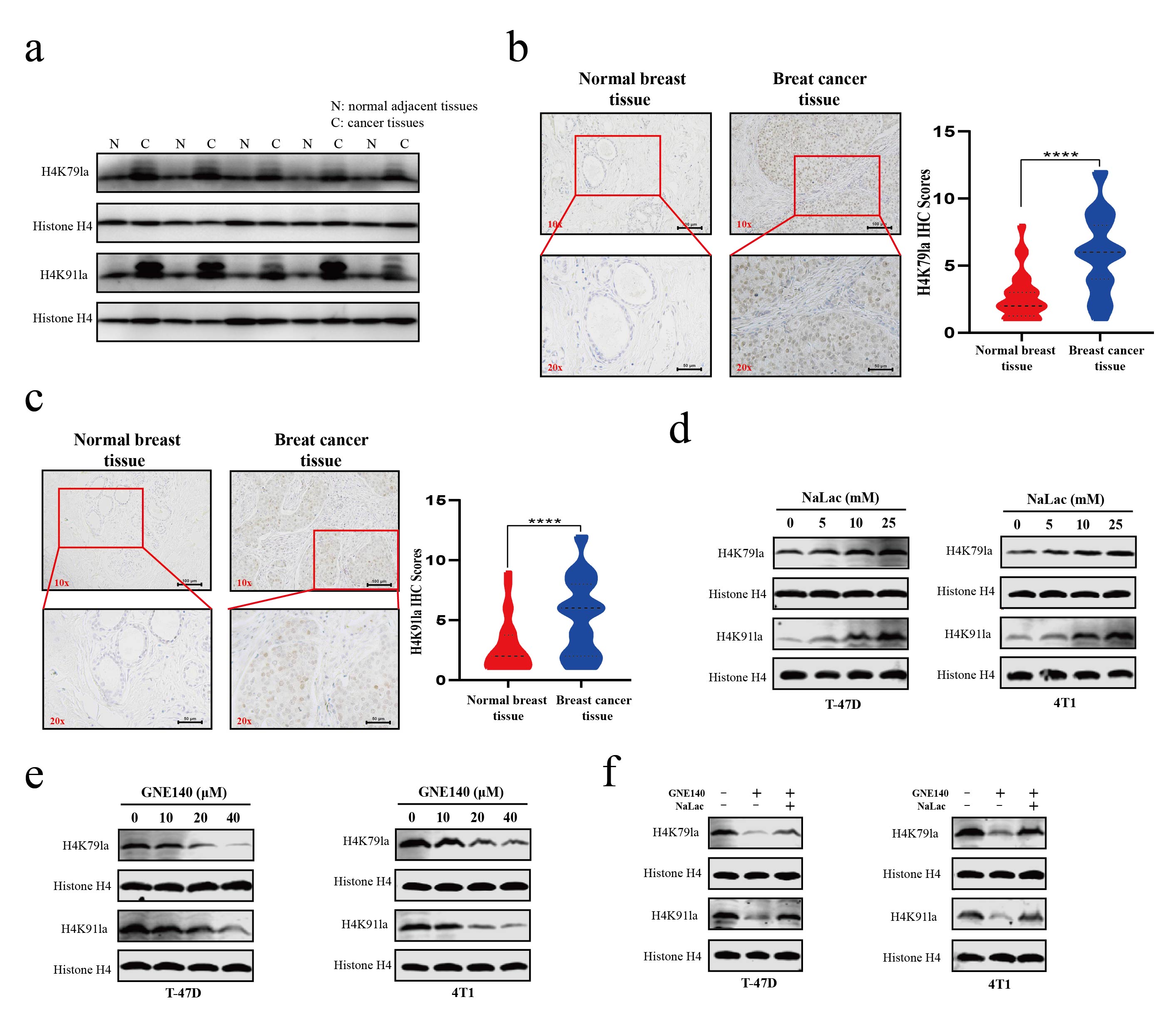

Supplement: Supplementary file 9 — Supplementary Material 9: Figure S9. H4K79la and H4K91la levels were significantly upregulated in BC and regulated by glycolysis and lactate levels. (a). Western blot analysis of H4K79la and H4K91la levels in BC tissues and paired normal adjacent tissues. (b). Representative IHC images and statistical results of H4K79la levels in BC tissues and normal tissues. Scale bars: 100µm (10x); 50µm (20x). (c). Representative IHC images and statistical results of H4K91la levels in BC tissues and normal tissues. Scale bars: 100µm (10x); 50µm (20x). (d). Western blot analysis of H4K79la and H4K91la levels in T-47D and 4T1 cells cultured in different concentrations of NaLac. (e). Western blot analysis of H4K79la and H4K91la levels in T-47D and 4T1 cells treated with different doses of GNE140. (f) Western blot analysis of H4K79la and H4K91la levels in T-47D and 4T1 cells treated with GNE140 then incubated with or without NaLac. [file 13046_2025_3512_MOESM9_ESM.jpg]

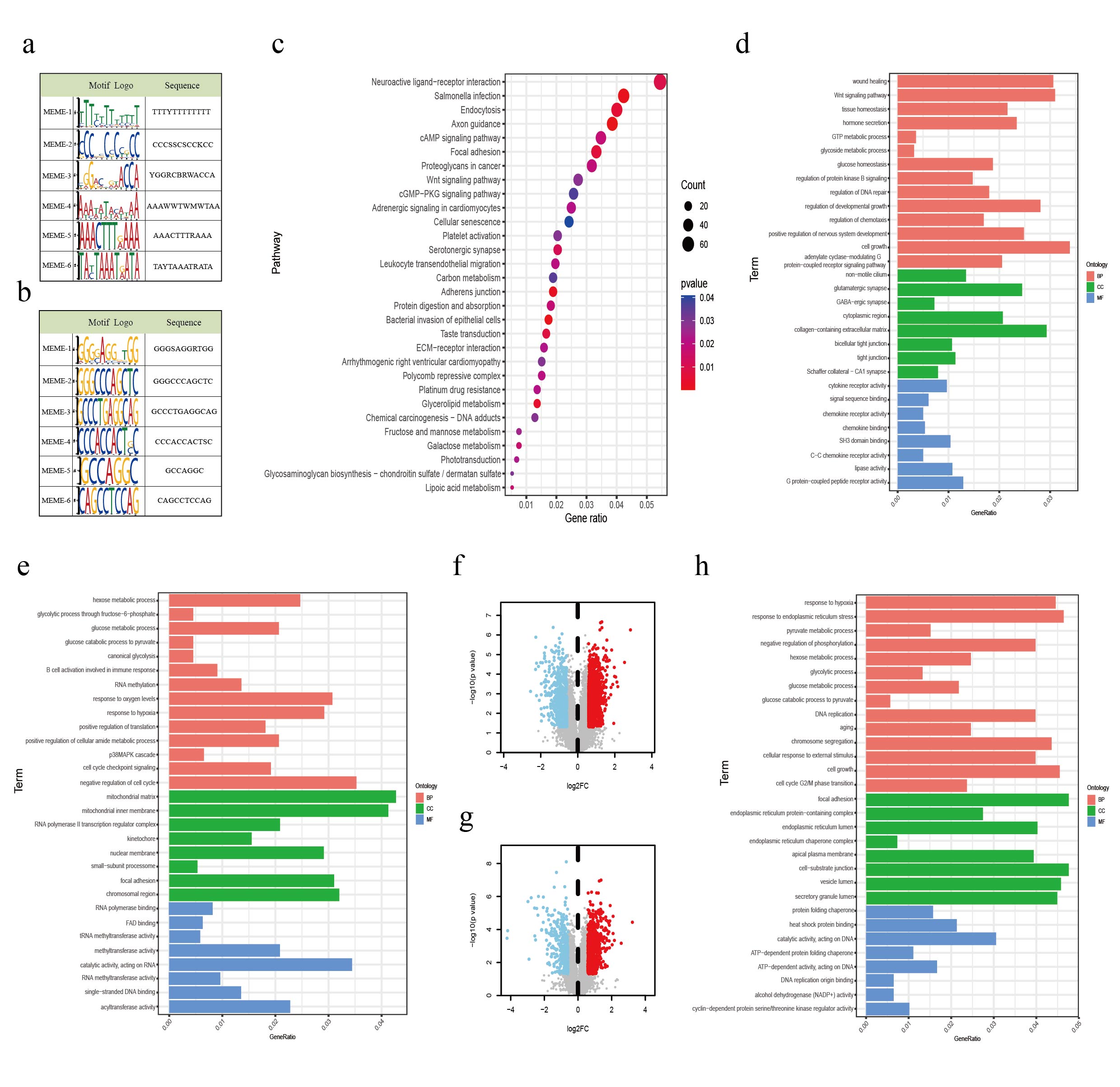

Supplement: Supplementary file 10 — Supplementary Material 10: Figure S10. GO and KEGG analysis of ChIP-seq and RNA-seq datasets. (a-b). Motif sequence analysis of H4K79la (a) and H4K91la (b) peaks. (c). KEGG analysis of H4K91la-realted promoter peak genes. (d). GO analysis of H4K91la-realted promoter peak genes. (e). GO analysis of differentially expressed genes after GNE140 treatment. (f). Volcano plot of differently expressed genes in GNE140-treated group based on RNA-seq. (g). Volcano plot of differently expressed genes in NaLac-treated group based on RNA-seq. (h). GO analysis of differentially expressed genes after NaLac treatment. [file 13046_2025_3512_MOESM10_ESM.jpg]

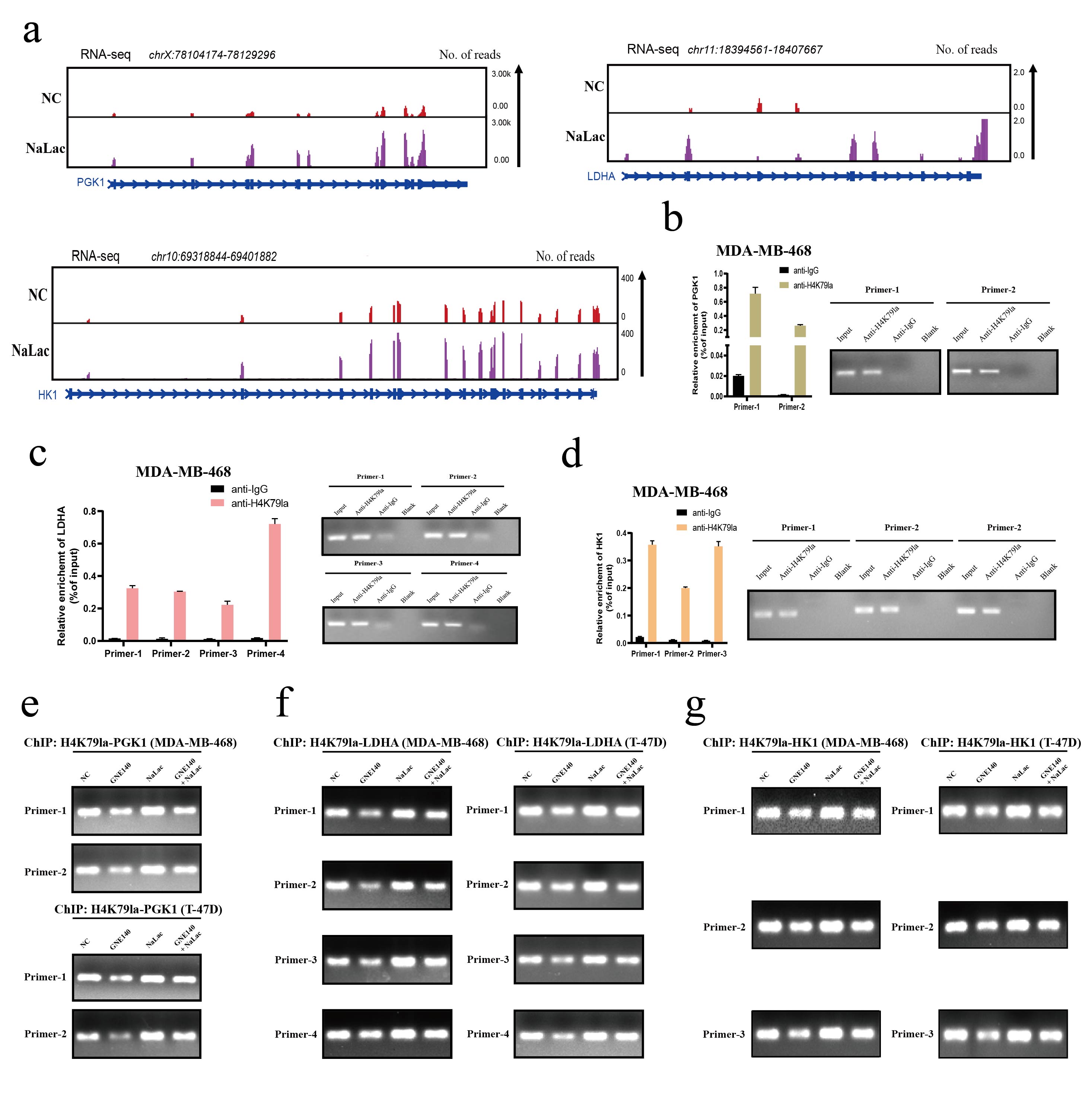

Supplement: Supplementary file 11 — Supplementary Material 11: Figure S11. H4K79la activated the transcription of LDHA, PGK1, and HK1. (a). Representative IGV tracks showing increased PGK1, LDHA, and HK1 expression upon NaLac treatment by RNA-seq. (b-d). DNA fragments from MDA-MB-468 cells were immunoprecipitated with the H4K79la-specific antibody and analyzed by qPCR using the indicated primers. (b). Verification of H4K79la enriched at PGK1 promoter region by specialized PGK1 primers. (c). Verification of H4K79la enriched at LDHA promoter region by specialized LDHA primers. (d). Verification of H4K79la enriched at HK1 promoter region by specialized HK1 primers. (e). Agarose gel electrophoresis corresponding to Figure 10C. (f). Agarose gel electrophoresis corresponding to Figure 10D. (g). Agarose gel electrophoresis corresponding to Figure 10E. [file 13046_2025_3512_MOESM11_ESM.jpg]

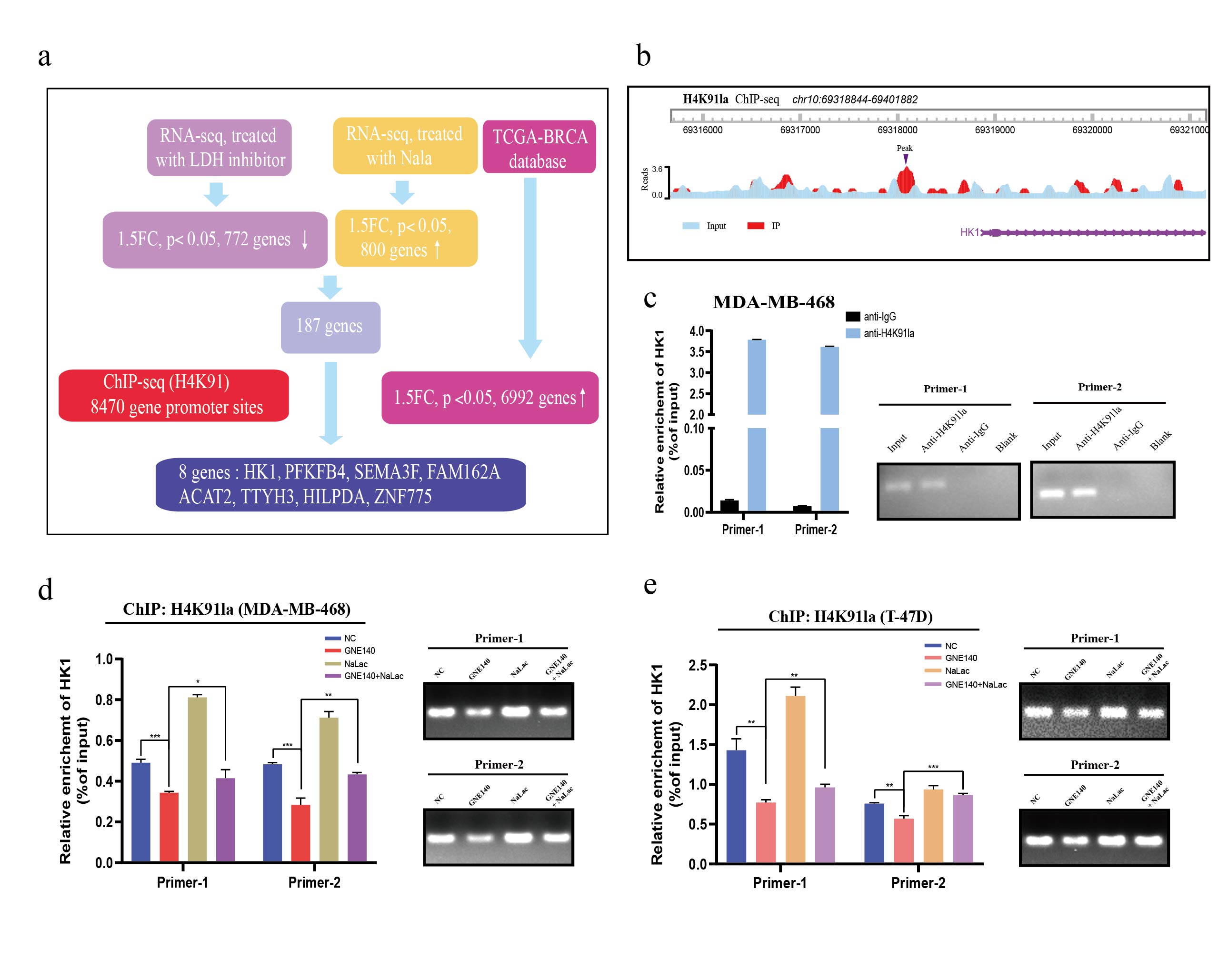

Supplement: Supplementary file 12 — Supplementary Material 12: Figure S12. H4K91la activated the transcription of HK1. (a). Combination of ChIP-seq, RNA-seq and TCGA-BRCA database to identify the potential downstream targets of H4K91la. (b). Representative IGV tracks showing enriched H4K91la modification in HK1 promotor regions by ChIP-seq. Arrows are the H4K91la peaks at the gene promotor. (c). DNA fragments from MDA-MB-468 cells were immunoprecipitated with the H4K91la-specific antibody and analyzed by qPCR using the specialized HK1 primers. (d-e). ChIP-qPCR assay of H4K91la status in the HK1 promoter region in MDA-MB-468 (d) and T-47D (e) cells upon treatment with GNE140, NaLac, or GNE140 combined NaLac. Error bars represent the mean±SD. * P < 0.05, ** P < 0.01, *** P < 0.001, **** P < 0.0001. [file 13046_2025_3512_MOESM12_ESM.jpg]

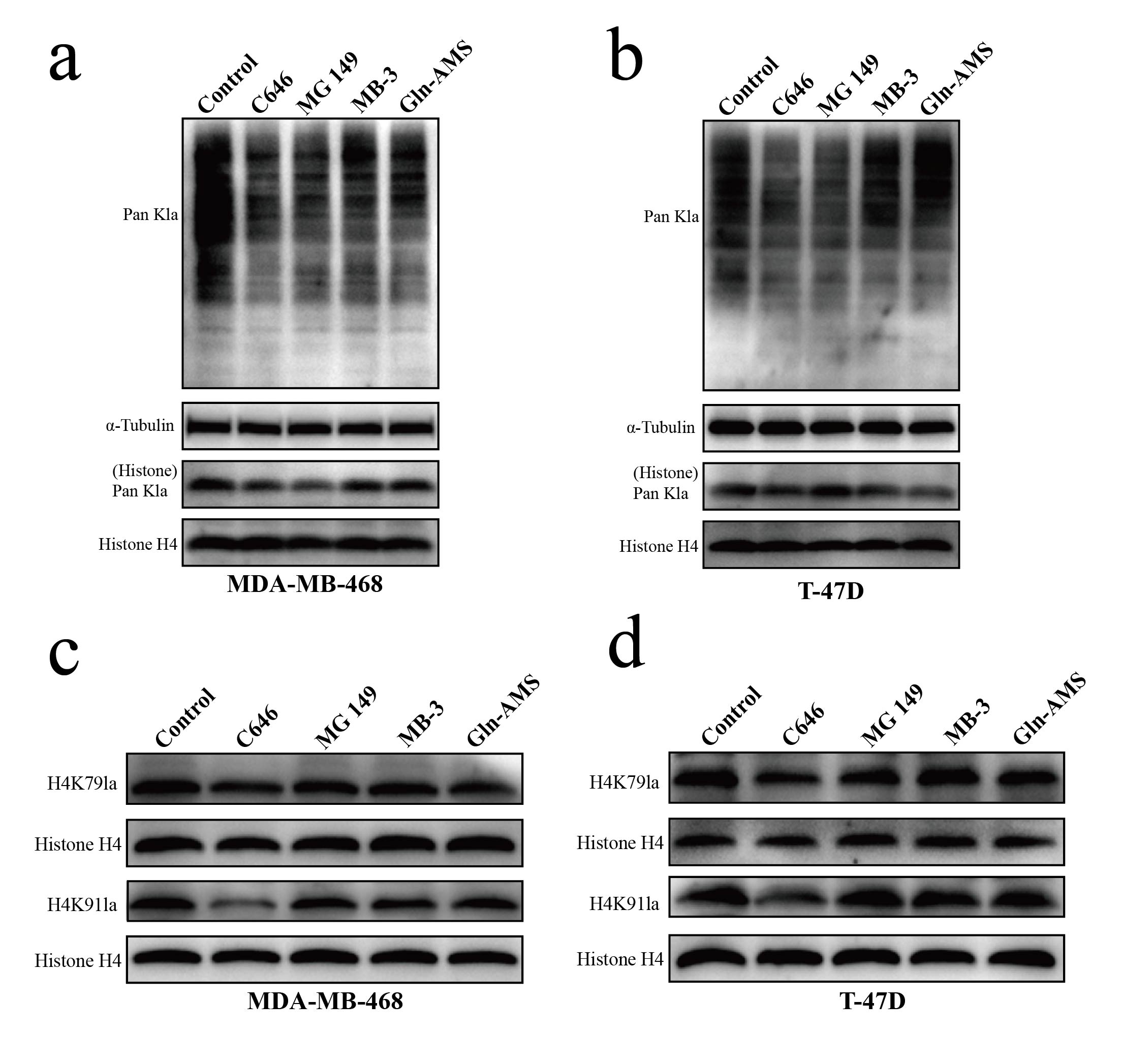

Supplement: Supplementary file 13 — Supplementary Material 13: Figure S13. P300 is the potential writer of H4K79la and H4K91la in BC cells. (a, b). Kla levels of MDA-MB-468 cell (a) and T-47D (b) cells treated with different inhibitors for 24h. C646 (20µm), MB-3 (30µm), MG 149 (20µm), and Gln-AMS (10µm). (c, d). H4K79la and H4K91la levels of MDA-MB-468 cell (c) and T-47D (d) cells treated with different inhibitors for 24h. [file 13046_2025_3512_MOESM13_ESM.jpg]
