# Supplementary material for: H4K79 and H4K91 histone lactylation, newly identified lactylation sites enriched in breast cancer
Source: J Exp Clin Cancer Res. 2025 Aug 23;44:252. doi: 10.1186/s13046-025-03512-6 (PMC12374308; doi:10.1186/s13046-025-03512-6)
Supplement: Supplementary file 15 — Supplementary Material 15: Table S2. siRNA sequences and primer sequences were used in this study. [file 13046_2025_3512_MOESM15_ESM.docx]

**Table S2. siRNA used in this study**

| **Gene** | **Target Sequences** |
| --- | --- |
| LDHA-si1 | CTTGGAAGATAAGTGGTTT |
| LDHA-si2 | GCTGGGAGTTCACCCATTA |
| LDHB-si1 | GGGAGCTTATTTCTTCAGA |
| LDHB-si2 | CAGTCCTGATTGCATCATA |
| EP300-NC | UUC UCC GAA CGU GUC ACG UTT |
| EP300-si1 | GUGGCACGAAGAUAUUACUTT |
| EP300-si2 | GCCCAAUGUUCUGGAAGAATT |

**RT-qPCR primers:**

| **Gene** | **Sequences (5’-3’)** |
| --- | --- |
| (Homo) ACTB-Forward | CATGTACGTTGCTATCCAGGC |
| (Homo) ACTB-Reverse | CTCCTTAATGTCACGCACGAT |
| (Homo) LDHA-Forward | ATGGCAACTCTAAAGGATCAGC |
| (Homo) LDHA-Reverse | CCAACCCCAACAACTGTAATCT |
| (Homo) LDHB-Forward | TGGTATGGCGTGTGCTATCAG |
| (Homo) LDHB-Reverse | TTGGCGGTCACAGAATAATCTTT |
| (Homo) HK1-Forward | GCTCTCCGATGAAACTCTCATAG |
| (Homo) HK1-Reverse | GGACCTTACGAATGTTGGCAA |
| (Homo) PGK1-Forward | TGGACGTTAAAGGGAAGCGG |
| (Homo) PGK1-Reverse | GCTCATAAGGACTACCGACTTGG |
| (Mus) Ldha-Forward | CAAAGACTACTGTGTAACTGCGA |
| (Mus) Ldha -Reverse | TGGACTGTACTTGACAATGTTGG |
| (Mus) Pgk1-Forward | ATGTCGCTTTCCAACAAGCTG |
| (Mus) Pgk1-Reverse | GCTCCATTGTCCAAGCAGAAT |
| (Mus) Hk1-Forward | GAGTCTGAGGTCTACGACACC |
| (Mus) Hk1-Reverse | CCCACGGGTAATTTCTTGTCC |
| (Mus) Actb-Forward | GTGACGTTGACATCCGTAAAGA |
| (Mus) Actb -Reverse | GCCGGACTCATCGTACTCC |

**H4K79 ChIP-qPCR primers:**

| **Gene** | **Sequences (5’-3’)** |
| --- | --- |
| PGK1- Primer-1-Forward | CCAAACTAGCCTTGGGAAAGAA |
| PGK1- Primer-1-Reverse | GCATGGCCCCTAAACTGTAATT |
| PGK1- Primer-2-Forward | GCCTGGCCCACTAAACTTCT |
| PGK1- Primer-2-Reverse | TGGTTTCCTAGCTCCCTTCA |
| HK1- Primer-1-Forward | GCATGGTGGCGCAATCA |
| HK1- Primer-1-Reverse | CTGCTCGGGAGGCTGAGAT |
| HK1- Primer-2-Forward | GGACTGTGACCTGGACAGGAA |
| HK1- Primer-2-Reverse | AGAGGGAGGGATGACGTAGGTT |
| HK1- Primer-3-Forward | CCTCCCATCCTACGTAACTTCCT |
| HK1- Primer-3-Reverse | TGGCCTGTCCCCATCCT |
| LDHA- Primer-1-Forward | CCCAACCCAAGCCTTTCAG |
| LDHA- Primer-1-Reverse | CCACTGCTGCTGGGAAGAG |
| LDHA- Primer-2-Forward | GCCCTCTAATTCCCTAATCATTTG |
| LDHA- Primer-2-Reverse | AAAGAGCCTTTTTCCCTGACTTAAT |
| LDHA- Primer-3-Forward | GAGTGCCTATTACGTGCCAGAA |
| LDHA- Primer-3-Reverse | ACCTTCAGTTTCCTCATCCATGA |
| LDHA- Primer-4-Forward | TGACAGGCCTTTGCAACAAG |
| LDHA- Primer-4-Reverse | GCCAGCGCCAGACTTTAAAT |

**H4K91 ChIP-qPCR primers:**

| **Gene** | **Sequences (5’-3’)** |
| --- | --- |
| HK1-Primer-1-Forward | CTGGACAAATGATCTGACCCATAG |
| HK1-Primer-1-Reverse | CCTCCCTAACCTGCCAAACA |
| HK1-Primer-2-Forward | CCTCCCATCCTACGTAACTTCCT |
| HK1-Primer-2-Reverse | TGGCCTGTCCCCATCCT |
