# Supplementary material for: H4K79 and H4K91 histone lactylation, newly identified lactylation sites enriched in breast cancer
Source: J Exp Clin Cancer Res. 2025 Aug 23;44:252. doi: 10.1186/s13046-025-03512-6 (PMC12374308; doi:10.1186/s13046-025-03512-6)
Supplement: Supplementary file 16 — Supplementary Material 16: Table S3. Analysis of the correlation between Kla level and clinicopathological characteristics in BC patients. [file 13046_2025_3512_MOESM16_ESM.docx]

**Analysis of the correlation between Kla level and clinicopathological characteristics in breast cancer patients.**

| Characteristics | Number of patients (n=234) | Protein lactylation expression | | *P*-value |
| --- | --- | --- | --- | --- |
|  |  | Low (n=101) | High (n=133) |  |
| Age | | | | |
| ≤50 (years) | 135 | 59 | 76 | 0.9508 |
| >50 (years) | 99 | 42 | 57 |  |
| Histological grade | | | | |
| 1 | 42 | 29 | 13 | 0.0001 |
| 2 | 164 | 67 | 97 |  |
| 3 | 28 | 5 | 23 |  |
| Stage | | | | |
| I | 40 | 25 | 15 | 0.0011 |
| II | 130 | 59 | 71 |  |
| III | 64 | 17 | 47 |  |
| Lymph node metastasis | | | | |
| Negative | 116 | 61 | 55 | 0.0059 |
| Positive | 118 | 40 | 78 |  |
| Molecular subtype | | | | |
| Luminal | 195 | 81 | 114 | 0.480286 |
| HER2+ | 13 | 6 | 7 |  |
| TNBC | 26 | 14 | 12 |  |
