# Supplementary material for: H4K79 and H4K91 histone lactylation, newly identified lactylation sites enriched in breast cancer
Source: J Exp Clin Cancer Res. 2025 Aug 23;44:252. doi: 10.1186/s13046-025-03512-6 (PMC12374308; doi:10.1186/s13046-025-03512-6)

Figure 1A

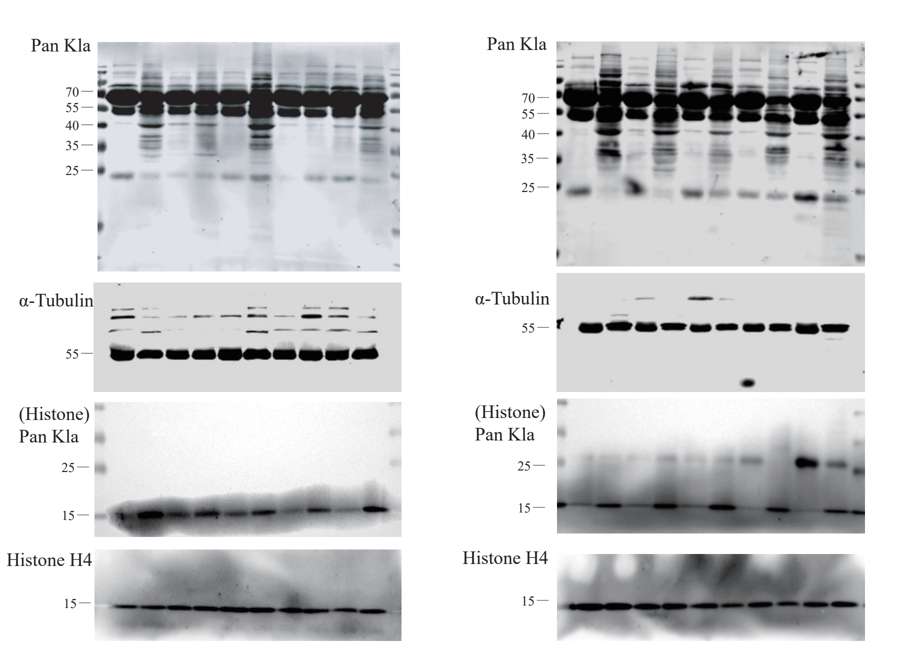

Figure 1H

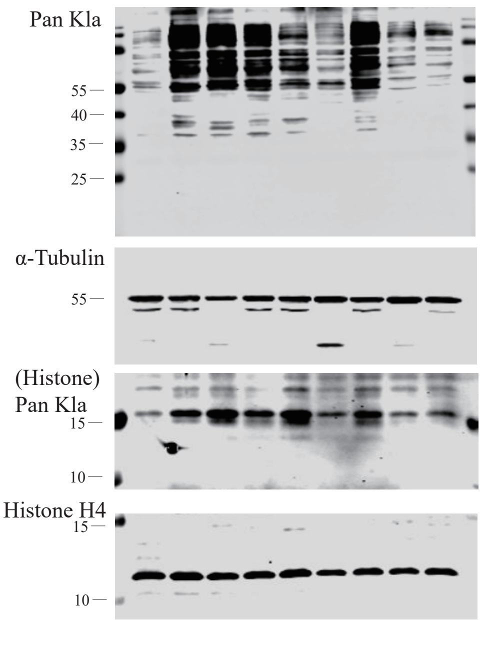

Figure 2B

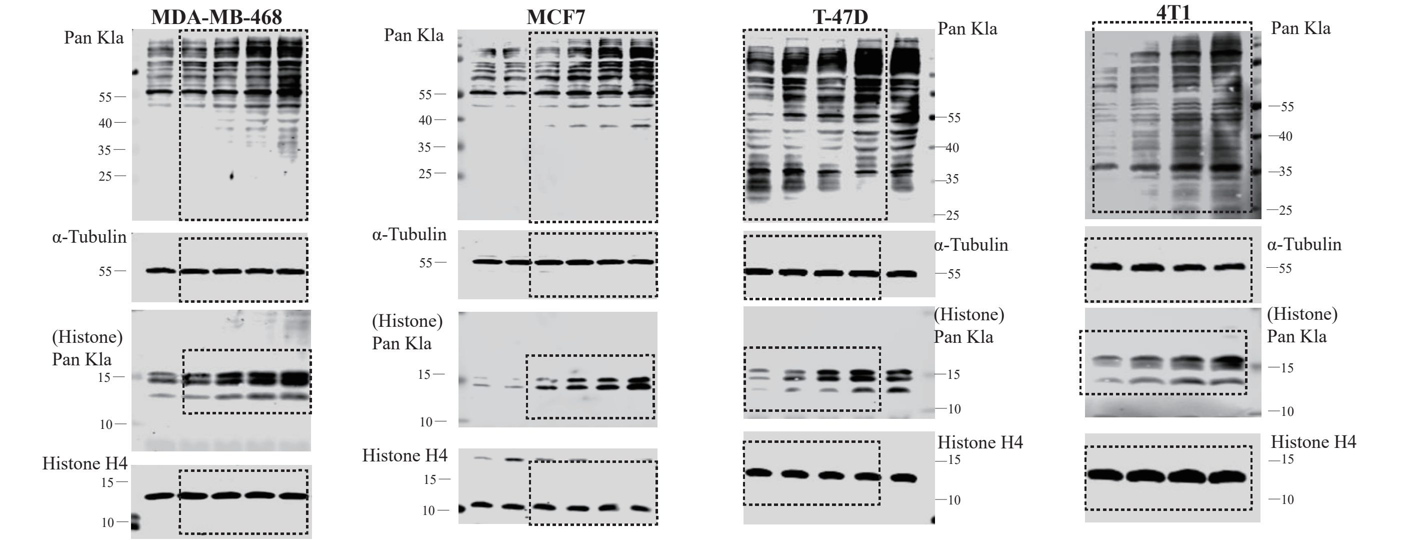

Figure 2D

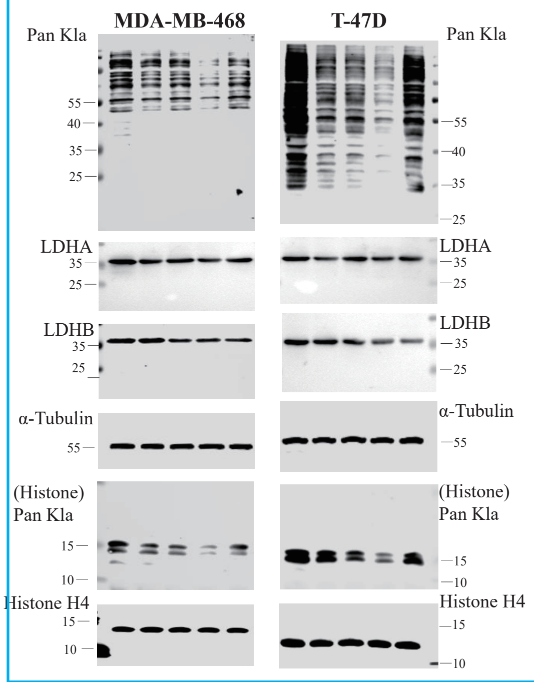

Figure 4A

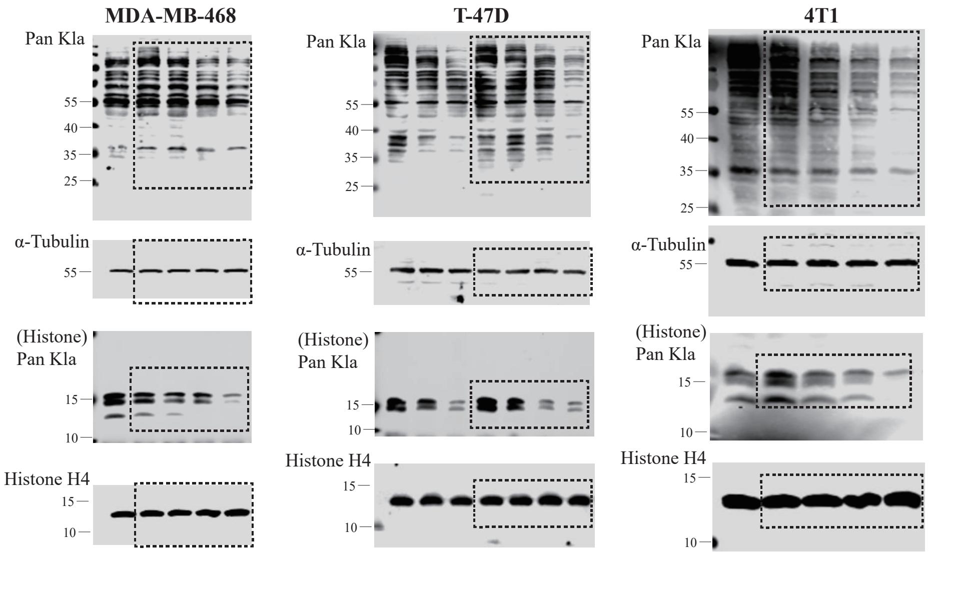

Figure 5A

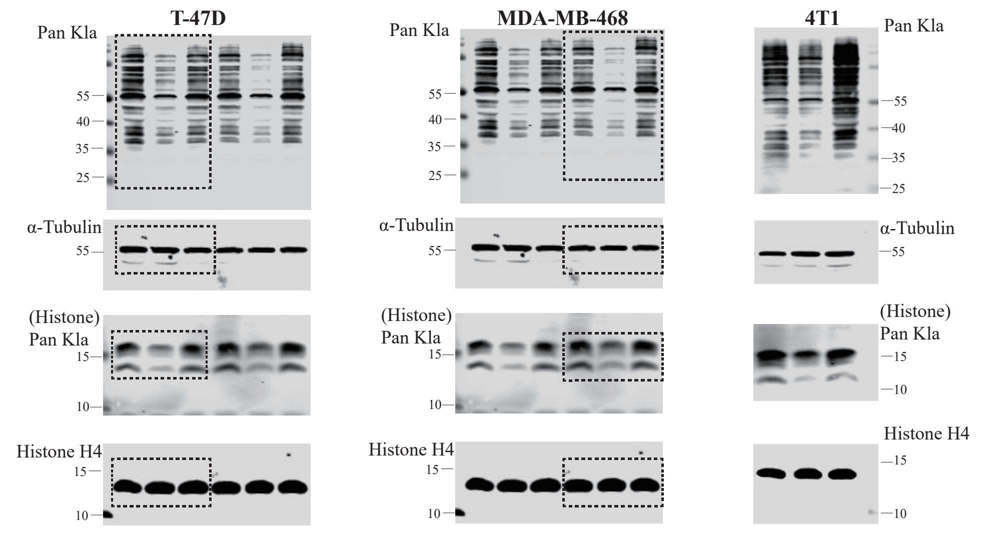

Figure 7F

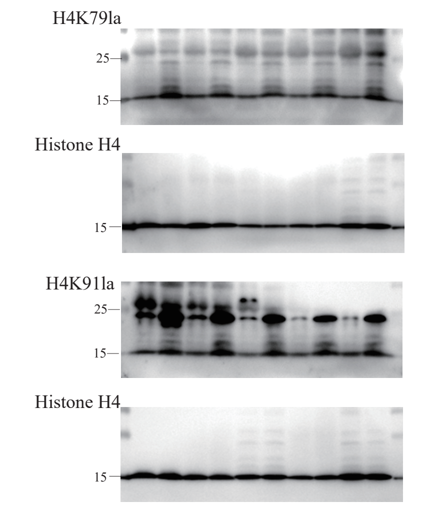

Figure 7G

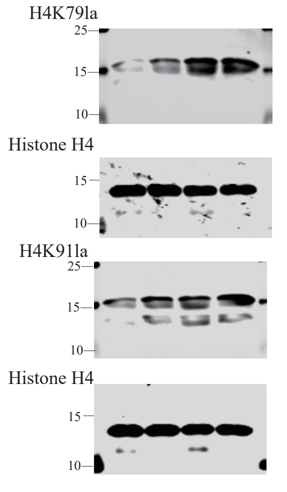

Figure 7H

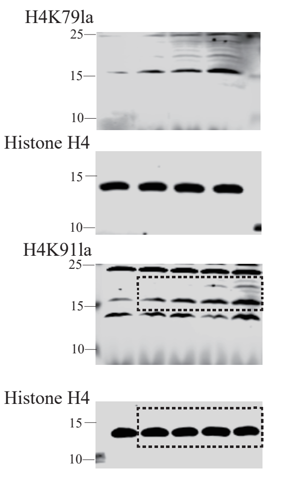

Figure 7I

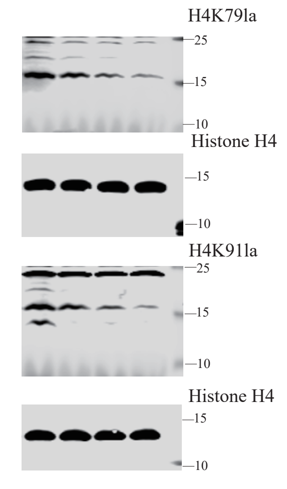

Figure 7J

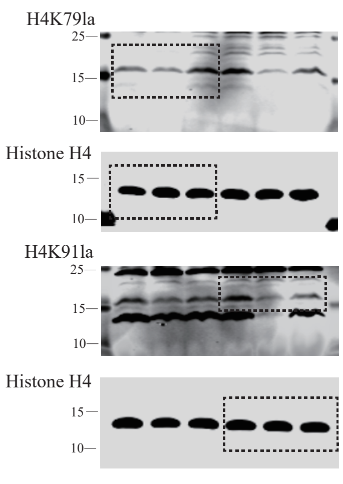

Figure 10B

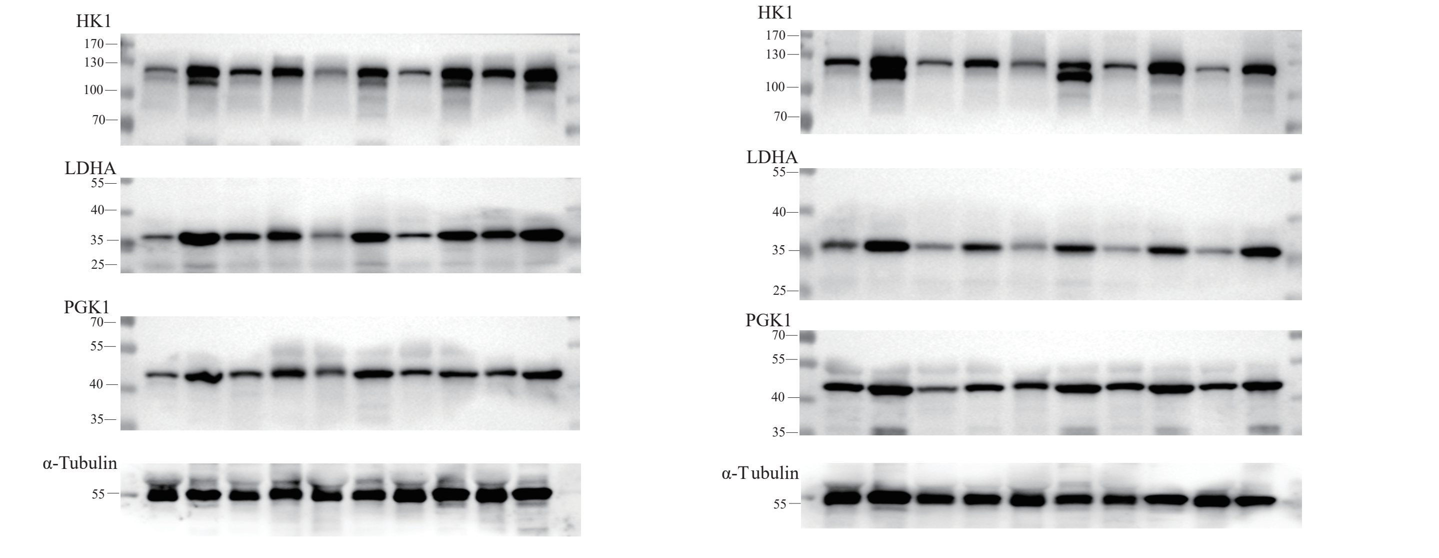

Figure 10F

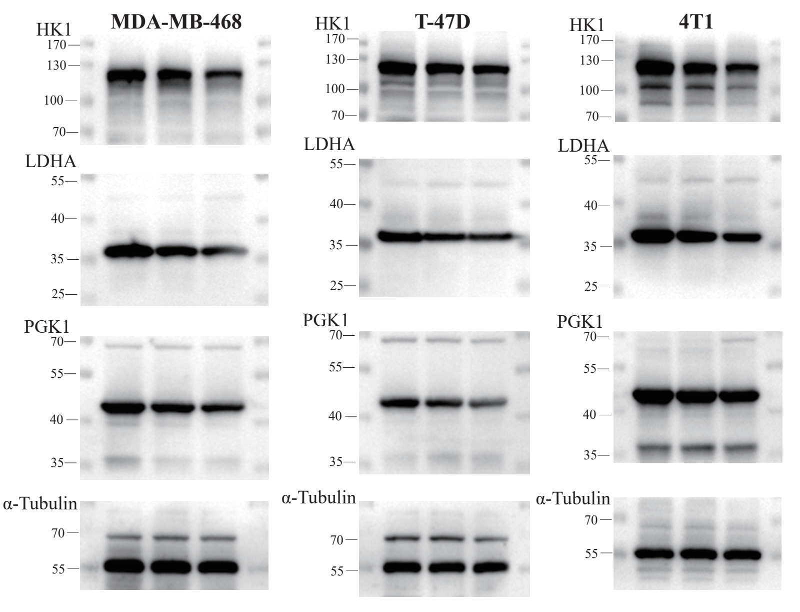

Figure 10G

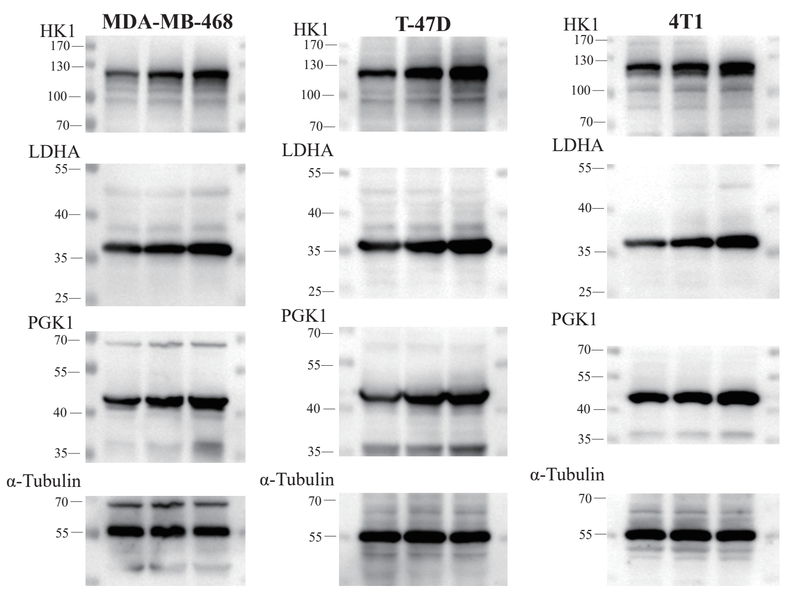

Figure 10H

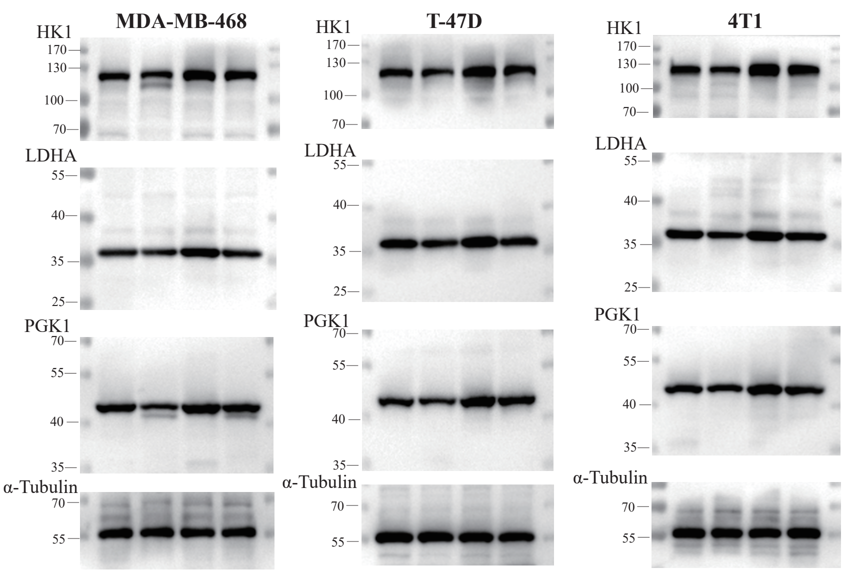

Figure 11D

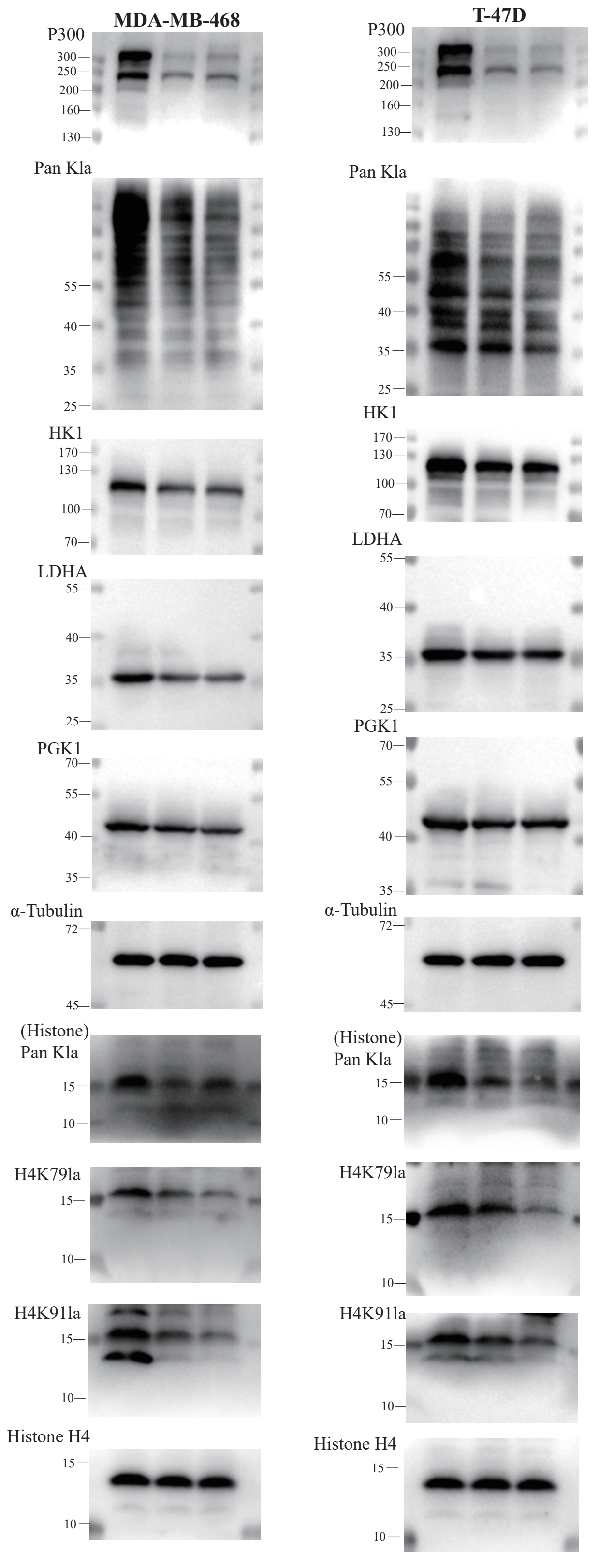

Figure S4b

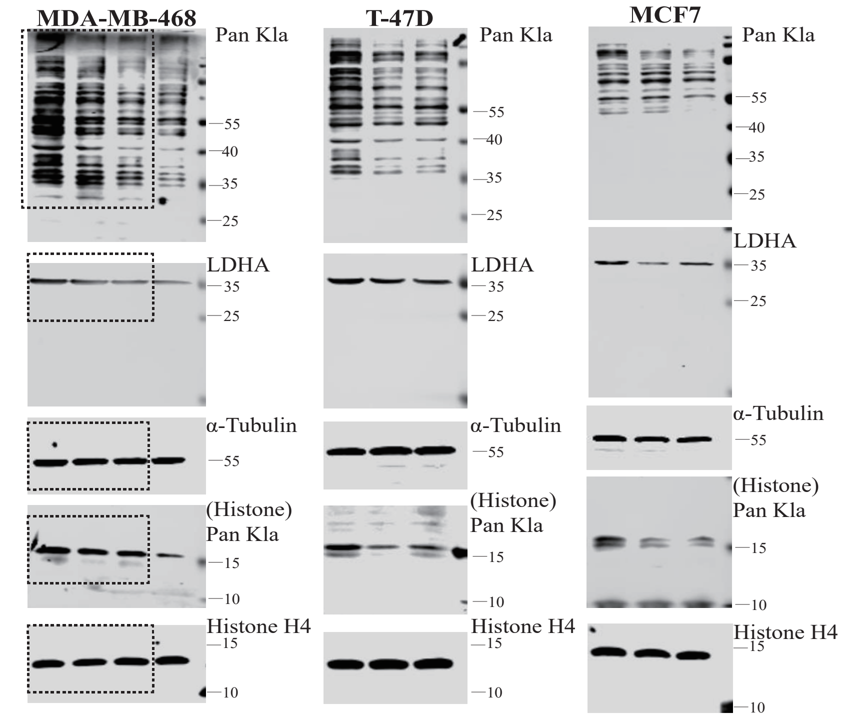

Figure S4c

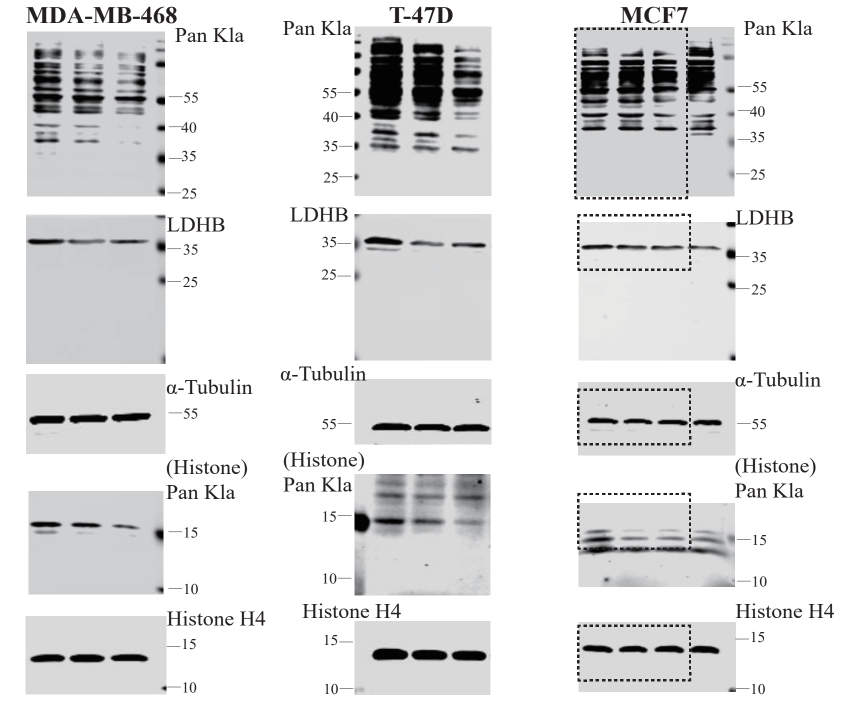

Figure S9a

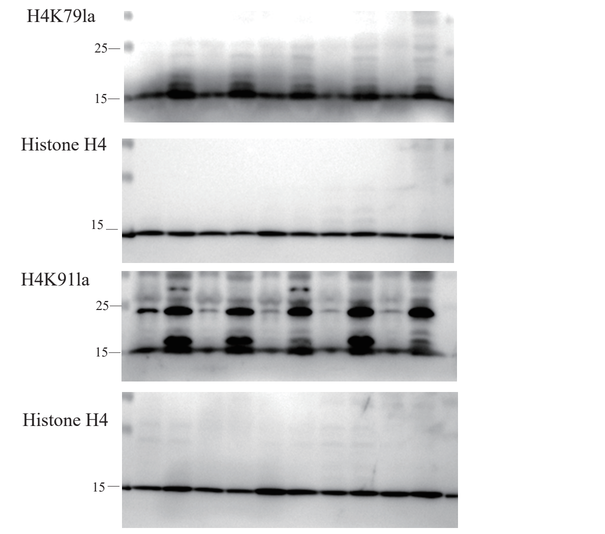

Figure S9d

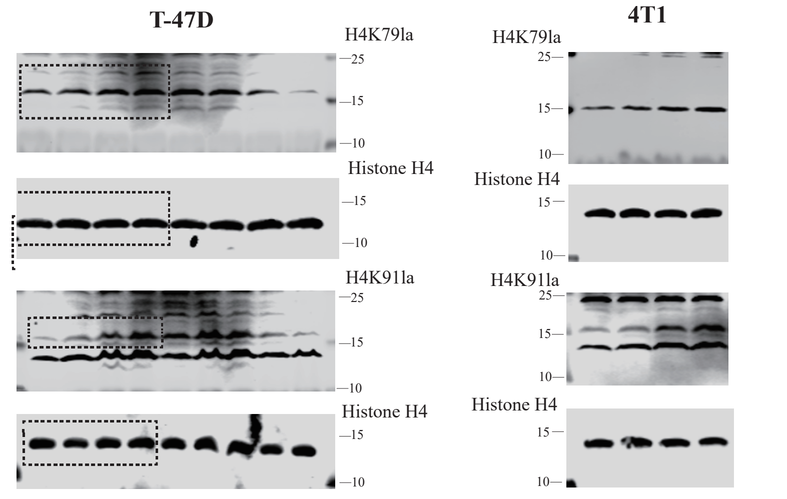

Figure S9e

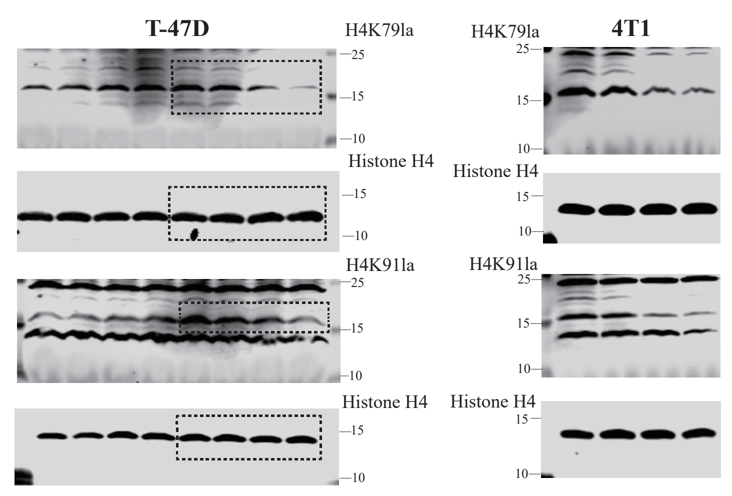

Figure S9f

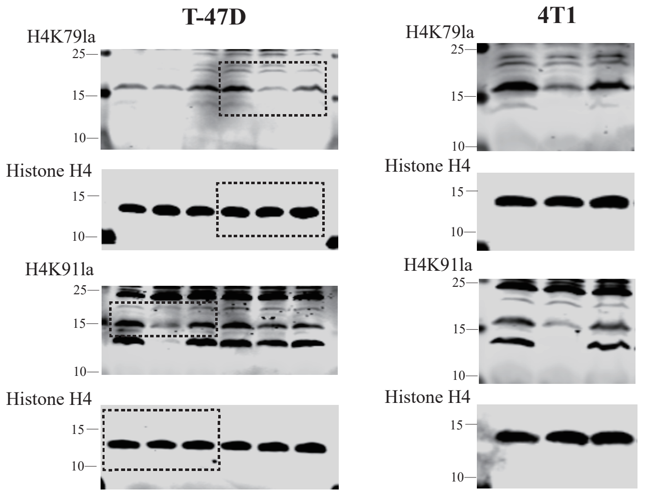

Figure S13a

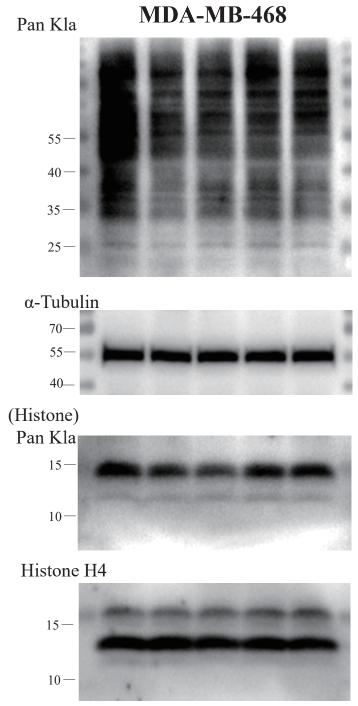

Figure S13b

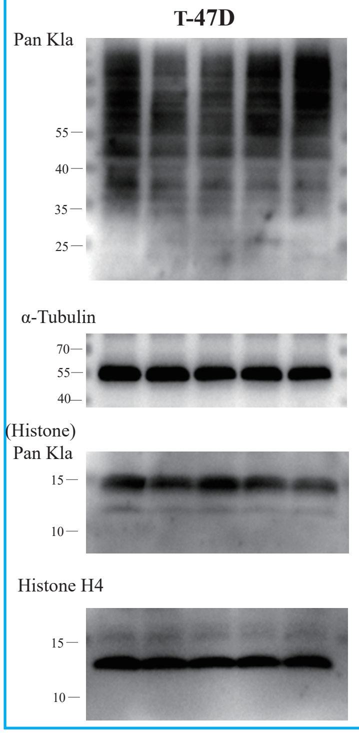

Figure S13c

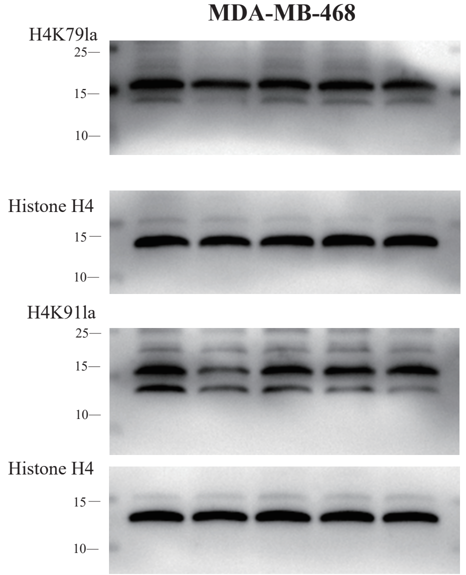

Figure S13d

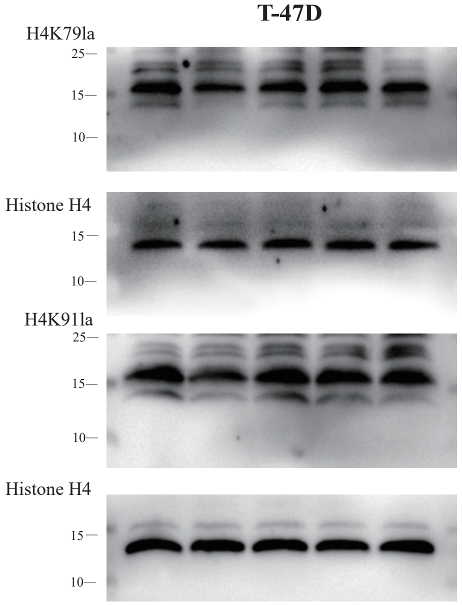

Supplement: Supplementary file 23 — Supplementary Material 23 [file 13046_2025_3512_MOESM23_ESM.pdf]
